# First Trimester Screening of Circulating C19MC microRNAs Can Predict Subsequent Onset of Gestational Hypertension

**DOI:** 10.1371/journal.pone.0113735

**Published:** 2014-12-15

**Authors:** Ilona Hromadnikova, Katerina Kotlabova, Lucie Hympanova, Jindrich Doucha, Ladislav Krofta

**Affiliations:** 1 Department of Molecular Biology and Cell Pathology, Third Faculty of Medicine, Charles University, Ruska 87, 100 00 Prague, Czech Republic; 2 Institute for the Care of the Mother and Child, Third Faculty of Medicine, Charles University, Podolske nabrezi 157/36, 147 00 Prague, Czech Republic; 3 Clinic of Obstetrics and Gynecology, Second Faculty of Medicine, Charles University, V Uvalu 84, 150 06 Prague, Czech Republic; Deutsches Krebsforschungszentrum, Germany

## Abstract

**Objective:**

The objective of the study was to evaluate risk assessment for gestational hypertension based on the profile of circulating placental specific C19MC microRNAs in early pregnancy.

**Study design:**

The prospective longitudinal cohort study of women enrolled at first trimester screening at 10 to 13 weeks was carried out (n = 267). Relative quantification of placental specific C19MC microRNAs (miR-516-5p, miR-517*, miR-518b, miR-520a*, miR-520h, miR-525 and miR-526a) was determined in 28 normal pregnancies and 18 pregnancies which developed gestational hypertension using real-time PCR and a comparative Ct method relative to synthetic C. elegans microRNA (cel-miR-39).

**Results:**

Increased extracellular C19MC microRNA plasmatic levels (miR-516-5p, p<0.001; miR-517*, p = 0.007; miR-520h, p<0.001; miR-518b, p = 0.002) were detected in patients destined to develop gestational hypertension. MiR-520h had the best predictive performance with a PPV of 84.6% at a 7.1% false positive rate. The combination of miR-520h and miR-518b was able to predict 82.6% of women at the same false positive rate. The overall predictive capacity of single miR-518b (73.3% at 14.3% FPR), miR-516-5p (70.6% at 17.9% FPR) and miR-517* (57.9% at 28.6% FPR) biomarkers was lower.

**Conclusion:**

The study brought interesting finding that the up-regulation of miR-516-5p, miR-517*, miR-520h and miR-518b is associated with a risk of later development of gestational hypertension. First trimester screening of extracellular miR-520h alone or in combination with miR-518b identified a significant proportion of women with subsequent gestational hypertension.

## Introduction

Since the placenta is being continuously remodelled during normal placental development, extracellular nucleic acids of both fetal and placental origin, packed into either trophoblast-derived apoptotic bodies or shedding syncytiotrophoblast microparticles, may be detected in maternal circulation during the course of normal gestation [1]–[5]. Latest findings revealed that microRNAs are also packed within exosomal nanoparticles released into the blood and extracellular compartment mediating the communication between diverse types of neighbouring or distant cells [6]–[9].

There has been a trend over the last 10 years to develop non-invasive methods utilizing quantification of cell-free nucleic acids inclusive of microRNAs in maternal circulation [5], [10]–[29]. The diagnostic potential of particular molecular biomarkers and their implementation in the current predictive and diagnostic algorithms for pregnancy related complications are subject of interest [5].

MicroRNAs belong to a family of small noncoding RNAs that regulate gene expression at the posttranscriptional level by degrading or blocking translation of messenger RNA (mRNA) targets [30], [31].

Recent studies have shown that clinically established preeclampsia is associated with alterations in extracellular microRNA expression [32]–[35]. Nevertheless, no differentiation between pregnancies with normal and fetal growth-restricted foetuses was observed when circulating microRNA expression levels were compared [36]–[38]. Nevertheless, the recent study of Whitehead et al. revealed up-regulation of several hypoxia-regulated microRNAs in pregnancies complicated by severe preterm fetal growth restriction compared to gestation-matched controls [39].

However, most of investigators focused on the study of those microRNAs, whose genes are located outside chromosome 19 miRNA clusters (C19MC and miR-371-3 cluster) or the chromosome 14 miRNA cluster (C14MC) that encode pregnancy-associated microRNAs [40]–[44].

We have previously identified C19MC microRNAs (miR-516-5p, miR-517*, miR-518b, miR-520a*, miR-520h, miR-525 and miR-526a) present in maternal plasma differentiating between normal pregnancies and non-pregnant individuals [45]. Significant increases in extracellular C19MC microRNAs levels (miR-516-5p, miR-517*, miR-518b, miR-520a*, miR-520h, miR-525 and miR-526a) over time in normally progressing pregnancies were observed [45], [46].

The results of our follow-up study indicated that the up-regulation of miR-516-5p, miR-517*, miR-520a*, miR-525 and miR-526a is a characteristic phenomenon of established preeclampsia [47].

The data resulting from our pilot study suggested the potential of extracellular C19MC microRNAs to differentiate, at the beginning of gestation (within 12^th^ to 16^th^ weeks), between patients at risk of later development of placental insufficiency related complications and normal pregnancies [46]. These data strongly supported the need for a more detailed exploration of extracellular microRNAs in maternal circulation with the view toward routine assessment into everyday practice, and recognition as potential biomarkers for placental insufficiency related pregnancy complications.

To our knowledge, no prospective study of women enrolled at first trimester screening to describe the profile of circulating C19MC microRNAs in the women at risk of subsequently developing gestational hypertension has been carried out. Here, we discuss for the first time the effectiveness of circulating C19MC microRNAs to predict the later occurrence of gestational hypertension.

Consequently, the function and functional relationship analysis of predicted targets of aberrantly expressed C19MC microRNAs in patients destined to develop gestational hypertension, was performed.

## Materials and Methods

### Patients

The study was designed in a prospective manner. The study cohort consisted of 267 consecutive Caucasian singleton pregnant women enrolled at first trimester screening at 10 to 13 weeks. The case cohort included 18 pregnancies which developed gestational hypertension and the control cohort that was chosen on the basis of equal times in storage and gestational age, included 28 normal pregnancies.

Gestational hypertension was defined as high blood pressure that developed after the twentieth week of pregnancy. Normal pregnancies were defined as those without complications who delivered full term, healthy infants weighting >2500 g after 37 completed weeks of gestation.

All patients provided written informed consent. The study was approved by the Ethics Committee of the Third Faculty of Medicine, Charles University in Prague. Gestational age was assessed using ultrasonography at 11 to 13 weeks and 6 days.

### Processing of samples

Nine millilitres of peripheral blood were collected into EDTA tubes and centrifuged twice at 1200 g for 10 min at room temperature. Plasma samples were stored at −80°C until subsequent processing.

Total RNA was extracted from 1 mL of plasma and 25 mg of normal placental tissue preserved in RNAlater (Ambion, Austin, USA) followed by an enrichment procedure for small RNAs using a mirVana microRNA Isolation kit (Ambion, Austin, USA). Trizol LS reagent was used in plasma samples for total RNA extraction from biological fluids (Invitrogen, Carlsbad, USA) and preceded the small RNAs enrichment procedure. To minimize DNA contamination, we treated the eluted RNA with 5 µL of DNase I (Fermentas International, Ontario, Canada) for 30 min at 37°C.

### Reverse transcriptase reaction

Each microRNA was reverse transcribed into complementary DNA using TaqMan MicroRNA Assay, containing microRNA-specific stem-loop RT primers, and TaqMan MicroRNA Reverse Transcription Kit (Applied Biosystems, Branchburg, USA) in a total reaction volume of 50 µL on a 7500 Real-Time PCR system (Applied Biosystems, Branchburg, USA) with following thermal cycling parameters: 30 minutes at 16°C, 30 minutes at 42°C, 5 minutes at 85°C, and then held at 4°C.

### Quantification of microRNAs

The characteristics of studied C19MC microRNAs are outlined in table 1.

**Table 1:** Characteristics of selected C19MC microRNAs.

Assay name	miRBase ID	NCBI Location Chromosome	microRNA sequence	Expression in placenta
hsa-miR-516-5p	hsa-miR-516b-5p	Chr.19: 58920508 - 58920592 [+]	5′-CAUCUGGAGGUAAGAAGCACUUU-3′	exclusively expressed
hsa-miR-517*	hsa-miR-517-5p	Chr.19: 54215522 - 54215608 [+]	5′-CCUCUAGAUGGAAGCACUGUCU-3′	high expression
hsa-miR-518b	hsa-miR-518b	Chr.19: 54205991 - 54206073 [+]	5′-CAAAGCGCUCCCCUUUAGAGGU-3′	exclusively expressed
hsa-miR-520a*	hsa-miR-520a-5p	Chr.19: 54194135 - 54194219 [+]	5′-CUCCAGAGGGAAGUACUUUCU-3′	high expression
hsa-miR-520h	hsa-miR-520h	Chr.19: 54245766 - 54245853 [+]	5′-ACAAAGUGCUUCCCUUUAGAGU-3′	exclusively expressed
hsa-miR-525	hsa-miR-525-5p	Chr.19: 54200787 - 54200871 [+]	5′-CUCCAGAGGGAUGCACUUUCU-3′	exclusively expressed
hsa-miR-526a	hsa-miR-526a	Chr.19: 54209506 - 54209590 [+]	5′-CUCUAGAGGGAAGCACUUUCU-3′	high expression

C19MC microRNAs were divided into two categories (microRNAs exclusively expressed in the placental tissue and those with high expression in the placental tissue) based on information in miRNAMap 2.0 database (http://mirnamap.mbc.nctu.edu.tw/index.php), where the Q-PCR experiments for monitoring the expression profiles of 224 human miRNAs in eighteen major normal tissues in humans are provided. For example, we indicated miR-516-5p and miR-518b as those to be exclusively expressed in the placental tissue, since according to the miRNAMap 2.0 database miR-516-5p was shown to be expressed only in the placental tissue and miR-518b to be highly expressed in the placental tissue and rarely expressed in testes. On the other hand, for instance miR-520a* showed besides high expression in the placental tissue also low expression in other human tissues involving adipose, bladder, brain, cervix, heart, kidney, liver, lung, muscle, ovary, prostate, small intestine, spleen, testes, thymus, thyroid and trachea.

15 µL of cDNA corresponding to each microRNA was mixed with components of TaqMan MicroRNA Assay, and the ingredients of the TaqMan Universal PCR Master Mix (Applied Biosystems, Branchburg, USA) in a total reaction volume of 35 µL. TaqMan PCR conditions were set as described in the TaqMan guidelines. The analysis was performed using a 7500 Real-Time PCR System. All PCRs were performed in duplicates. A sample was considered positive if the amplification signal occurred before the 40^th^ threshold cycle.

The expression of particular microRNA in maternal plasma was determined using the comparative Ct method [48] relative to the expression of the same microRNA in the reference sample. RNA fraction highly enriched for small RNA isolated from the fetal part of one randomly selected placenta derived from gestation with normal course (the part of the placenta derived from the chorionic sac that encloses the embryo, consisting of the chorionic plate and villi) was used as a reference sample for relative quantification throughout the study.

Synthetic C. elegans microRNA (cel-miR-39, Qiagen, Hilden, Germany) was used as an internal control for variations during the preparation of RNA, cDNA synthesis, and real-time PCR. Due to a lack of generally accepted standards, all experimental real-time qRT-PCR data were normalized to cel-miR-39, as it shows no sequence homology to any human microRNA. 1 µl of 0.1 nM cel-miR-39 was spiked in after incubation with Trizol LS reagent to human plasma and reference samples. The following form of equation was used to compare the gene expression between various samples:
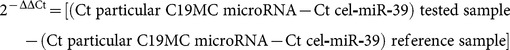



### Statistical analysis

Imprecision of the assays is indicated as percentage coefficients of variations (%CV). Normality of the data was assessed using Shapiro-Wilk test, which indicated that our data did not follow a normal distribution. Therefore, microRNA levels were compared between groups by non-parametric test (the Mann-Whitney U test) using Statistica software (version 9.0; StatSoft, Inc., USA). Since the Bonferroni correction was used to address the problem of multiple comparisons (altogether 6 placental specific microRNAs were analysed), the significance level was established at a *p-*value of p<0.0083 (α = 0.05/6).

Receivers operating characteristic (ROC) curves were constructed to calculate the area under the curve (AUC) and the best cut-off point for particular placental specific microRNA was used in order to calculate the respective sensitivity, specificity, predictive values and likelihood ratios in the prediction of gestational hypertension.

Comparison of ROC curves was done with the method of DeLong et al. [49] using MedCalc statistical software (MedCalc Software bvba, Ostend, Belgium). The software gave the difference between the areas under the ROC curves, with standard errors, 95% confidence intervals and p-values.

### Function and functional relationship analysis of target genes of studied C19MC microRNAs

The function and functional relationship analysis of predicted targets of studied C19MC microRNAs were performed. Mainly the predicted targets with close relation to gestation were subject of interest. The data were collected from miRDB database (http://mirdb.org). All the targets were predicted by a bioinformatics tool MirTarget2, which was developed by analysing thousands of genes impacted by miRNAs with an SVM learning machine.

The miRDB database is interconnected to the NCBI database (http://www.ncbi.nlm.nih.gov/gene/), where the description of proteins encoded by predicted genes is provided. Comprehensive and systematic search for each predicted target of particular mature C19MC microRNAs (miR-516-5p, miR-517^*^, miR-520h and miR-518b), that have been shown to be upregulated in patients with later onset of gestational hypertension, in relation to gestation was made using the PubMed database (http://www.ncbi.nlm.nih.gov/pubmed/).

## Results

Of 267 pregnant women enrolled at first trimester screening, 25 were lost for follow-up, 18 developed gestational hypertension (6.7%), 15 were diagnosed with other pregnancy-related complications (10 preeclampsia, 3 intrauterine growth restriction, 2 small for gestational age foetuses) and 209 had normal course of gestation (78.3%).

Unfortunately, miR-526a displayed late amplification curves (median Ct 39.61) in first trimester plasma samples, and therefore it was excluded from further analysis.

### Intra- and inter- assay variability for particular microRNA assays

Intra- and interassay reproducibility testing for particular microRNA assays using plasma samples derived from both pregnancies with normal and complicated course of gestation showed an imprecision of 3.01%–5.68% in within-assay comparisons (miR-516-5p: 3.67%, miR-517*: 3.01%, miR-518b: 3.55%, miR-520a*: 5.16%, miR-520h: 3.12%, and miR-525: 5.68%) and 12.61%–14.92% in between-assay comparisons (miR-516-5p: 14.92%, miR-517*: 13.53%, miR-518b: 14.23%, miR-520a*: 13.01%, miR-520h: 13.98%, and miR-525: 12.61%), respectively.

### Up-regulation of circulating C19MC microRNAs in pregnancies which subsequently developed gestational hypertension

**Figure 1:**
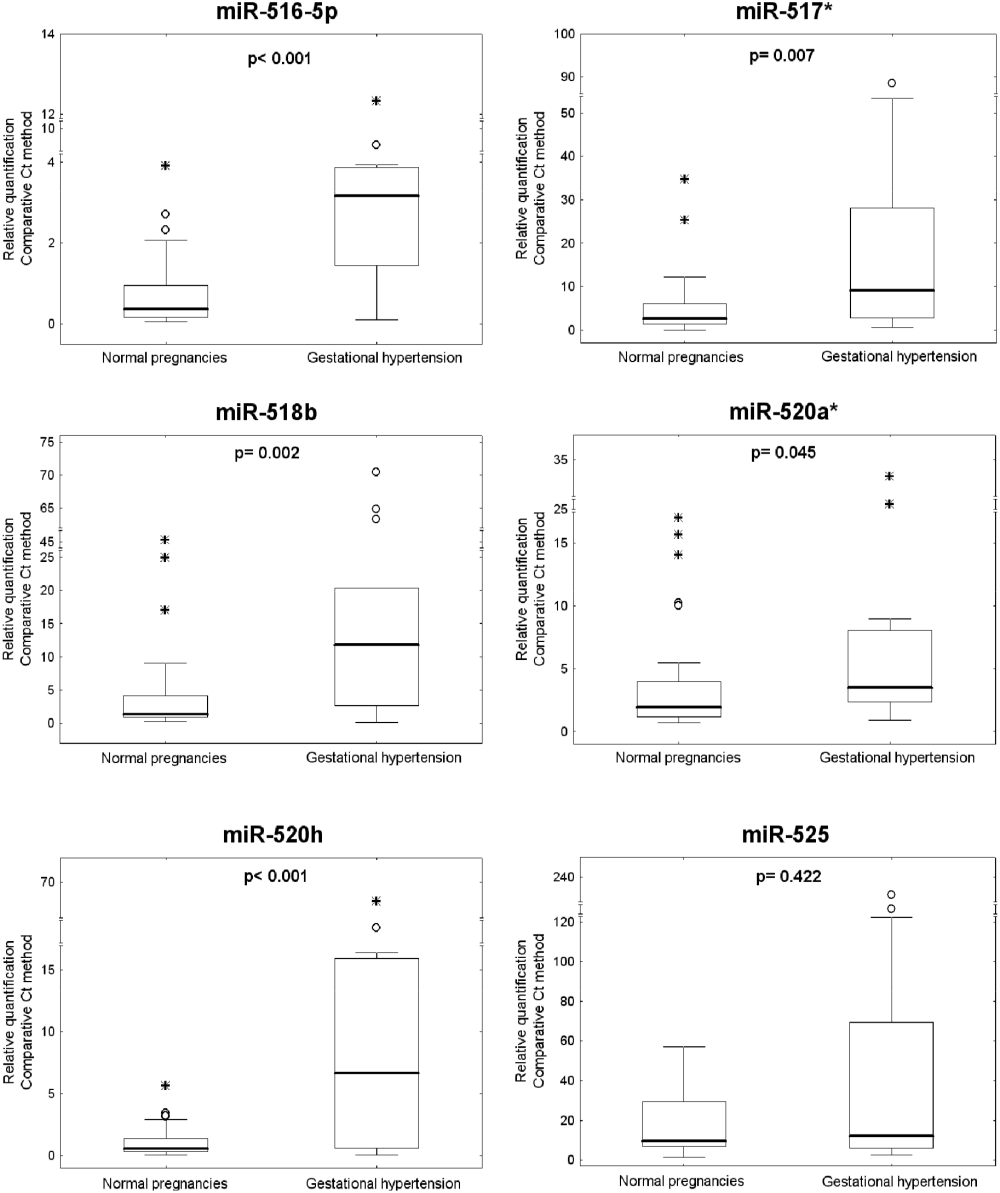
Up-regulation of circulating C19MC microRNAs in pregnancies which developed gestational hypertension. Relative quantification data were expressed as box plots of individual microRNAs in cohorts of normal and complicated pregnancies using Statistica software. The upper and lower limits of the boxes represent the 75^th^ and 25^th^ percentiles, respectively. The upper and lower whiskers represent the maximum and minimum values that are no more than 1.5 times the span of the interquartile range (range of the values between the 25^th^ and the 75^th^ percentiles). The median is indicated by the line in each box. Outliers are indicated by circles and extremes by asterisks.

Overall, increased plasmatic levels of **miR-516-5p** (p<0.001), miR-517*(p = 0.007), **miR-520h** (p<0.001) and **miR-518b** (p = 0.002) were observed in maternal plasma samples derived from first trimester screening of those women who subsequently developed gestational hypertension compared to normal pregnancies. No difference in plasmatic levels of miR-520a* (p = 0.044) and **miR-525** (p = 0.422) between the control cohort and the cohort of patients destined to develop gestational hypertension was identified (Fig. 1).

### First trimester screening of circulating C19MC microRNAs in the identification of pregnancies with later onset of gestational hypertension

**Figure 2:**
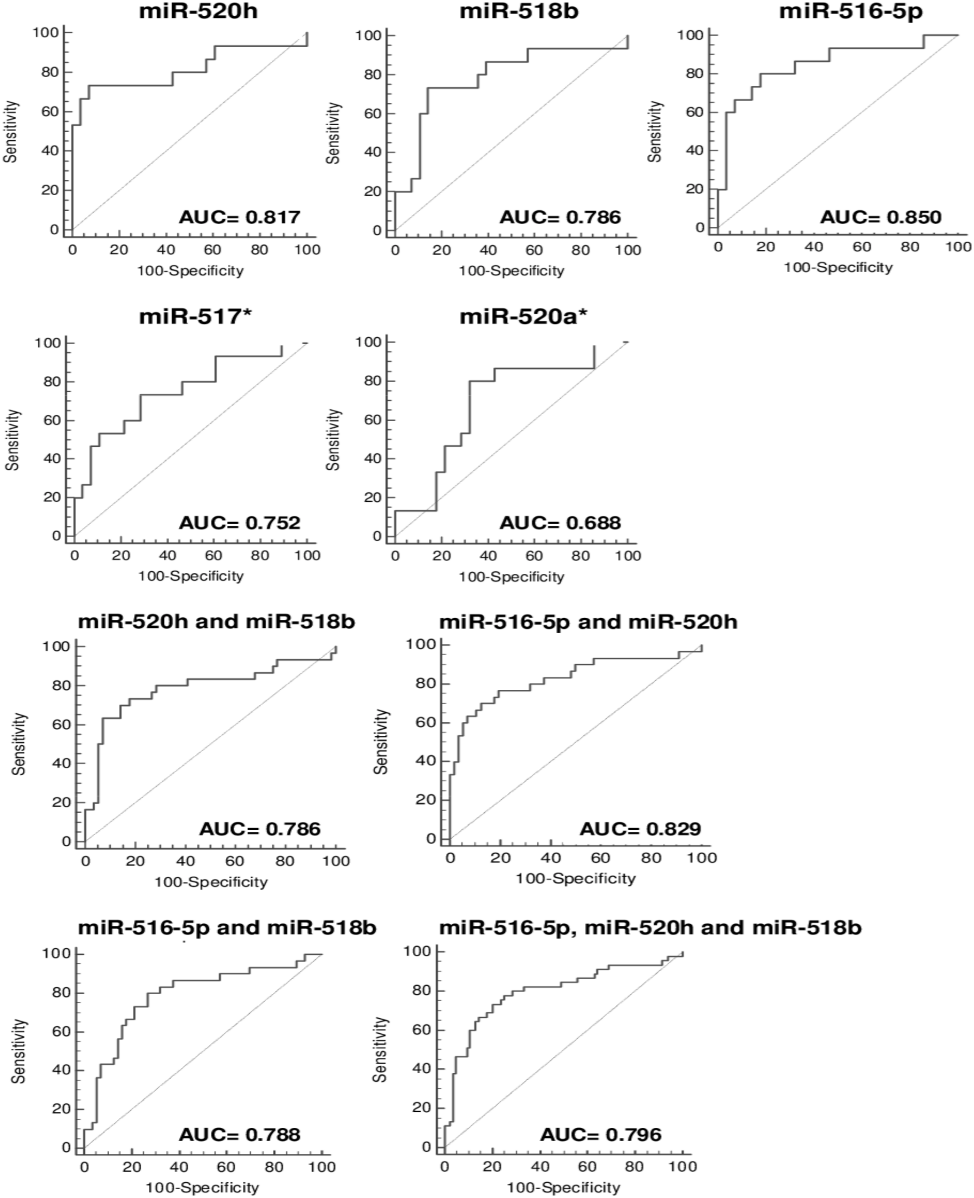
Receiver operating characteristic curves – evaluation of the effectiveness of circulating C19MC microRNAs to predict the development of gestational hypertension.

Table 2 and 3 display the predictive accuracy of maternal plasma concentrations of placental specific microRNAs in early pregnancy in the identification of gestational hypertension using cut-offs derived from the ROC curves. Firstly, the predictive accuracy of single first trimester plasmatic microRNA markers was assessed. The largest area under the curve was observed for miR-516-5p (0.850), miR-520h (0.817) and miR-518b (0.786). Using miR-517* and miR-520a* prediction rules for gestational hypertension had lower area under the curve of 0.752/0.688, respectively (Fig. 2, Table 2). The predictive performance of miR-525 was not reported since the AUC was not significant (0.576, p = 0.4685). Pairwise comparison between ROC curves revealed that the difference between AUCs of miR-516-5p and miR-518b was 0.064 and this difference was significant (95% CI, 0.012–0.116, p = 0.014), while the difference between AUCs of miR-516-5p and miR-520h (0.033), was not significant (95% CI, −0.021–0.088, p = 0.234). Similarly, no difference was observed between AUCs of miR-520h and miR-518b (0.031), (95% CI, −0.022–0.084, p = 0.253).

**Table 2:** Predictive accuracy of first trimester maternal plasma C19MC microRNA markers for the diagnosis of gestational hypertension.

*miRNA*	*AUC*	*ROC curve*	*Cutoff*	*Sensitivity*	*Specificity*	*PPV*	*NPV*	*Positive likelihood ratio*	*Negative likelihood ratio*
	*(95% CI)*	*p-value*						*(95% CI)*	*(95% CI)*
**miR-520h**	0.817	0.0002	>3.1986	73.30%	92.90%	84.60%	86.70%	10.27	0.29
	(0.669−0.918)							(2.6−40.4)	(0.1−0.7)
**miR-518b**	0.786	0.0004	>5.5955	73.30%	85.70%	73.30%	85.70%	5.13	0.31
	(0.634−0.896)							(2.0−13.4)	(0.1−0.7)
**miR-516-5p**	0.85	<0.0001	>1.2013	80.00%	82.10%	70.60%	88.50%	4.48	0.24
	(0.708−0.940)							(1.9−10.3)	(0.09−0.7)
miR-517*	0.752	0.0021	>3.761	73.30%	71.40%	57.90%	83.30%	2.57	0.37
	(0.597−0.871)							(1.3−5.0)	(0.2−0.9)
miR-520a*	0.688	0.031	>2.2471	80.00%	67.90%	57.10%	86.40%	2.49	0.29
	(0.529−0.820)							(1.4−4.5)	(0.1−0.8)

**Table 3:** Predictive accuracy of first trimester maternal plasma C19MC microRNA markers combination for the diagnosis of gestational hypertension.

*miRNA*	*AUC*	*ROC curve*	*Cutoff*	*Sensitivity*	*Specificity*	*PPV*	*NPV*	*Positive likelihood ratio*	*Negative likelihood ratio*
	*(95% CI)*	*p-value*						*(95% CI)*	*(95% CI)*
**miR-520h and**	0.786	<0.0001	>5.6161	63.30%	92.90%	82.60%	82.50%	8.87	0.39
**miR-518b**	(0.685−0.867)							(3.3−23.7)	(0.2−0.6)
**miR-516-5p and**	0.829	<0.0001	>0.0002	70.00%	87.50%	75.00%	84.50%	5.6	0.34
**miR-520h**	(0.733−0.902)							(2.7−11.6)	(0.2−0.6)
**miR-516-5p and**	0.788	<0.0001	>0.0002	80.00%	73.20%	61.50%	87.20%	2.99	0.27
**miR-518b**	(0.687−0.869)							(1.9−4.8)	(0.1−0.6)
**miR-516-5p,**	0.796	<0.0001	>0.0002	73.30%	79.80%	66.00%	84.80%	3.62	0.33
**miR-520h and miR-518b**	(0.716−0.862)							(2.3−5.7)	(0.2−0.5)

AUC: area under the curve, ROC: receiver operating characteristic; PPV: positive predictive value; NPV: negative predictive value.

The best positive predictive value (84.6%) and specificity (92.9%) was observed for miR-520h. Although miR-516-5p had significantly higher AUC than miR-518b, finally miR-518b showed better PPV (73.3%) and specificity (85.7) than miR-516-5p. MiR-516-5p predicted the subsequent occurrence of gestational hypertension with a sensitivity of 80.0%, a specificity of 82.1% and a PPV of 70.6%. Overall, the likelihood ratios for a positive test for these three best placental specific microRNA markers were large to small, ranging between 10.27 and 4.48.

However, whilst raised plasmatic levels of miR-517* have been observed in the first trimester, the overall predictive capacity for gestational hypertension was lower (sensitivity 73.3%, specificity 71.4%, and PPV 57.9%).

The areas under the curves were comparable between various combinations of three selected C19MC microRNA biomarkers (miR-516-5p, miR-520h and miR-518b). Pairwise comparison between ROC curves revealed no difference between AUCs of the following combinations of C19MC microRNAs (miR-516-5p and miR-518b vs. miR-516-5p and miR-520h: 0.788 vs. 0.828, 95% CI, −0.007–0.089, p = 0.097; miR-516-5p and miR-518b vs. miR-518b and miR-520h: 0.788 vs. 0.786, 95% CI, −0.090–0.093, p = 0.969; miR-516-5p and miR-520h vs. miR-518b and miR-520h: 0.829 vs. 0.786, 95% CI, −0.014–0.099, p = 0.137).

First trimester screening based on the combination of two placental specific microRNAs (miR-520h and miR-518b) showed the highest accuracy for the prediction of gestational hypertension; it was able to identify women at risk of subsequently developing complication with a PPV of 82.6% at a specificity of 92.9% (Fig. 2, Table 3). The combination of these two placental specific microRNAs showed the same specificity as the miR-520h biomarker, and higher a PPV (82.6%) compared to the single miR-518b biomarker. Other C19MC microRNA combinations (miR-516-5p and miR-520h or miR-516-5p and miR-518b) showed lower PPV than the miR-520h biomarker alone or in combination with miR-518b (Fig. 2, Table 3). That's why there was no additive effect of using of miR-516-5p in the combination with miR-520h and/or miR-518b, and the use of all three C19MC microRNA biomarkers had no advantage over using single miR-520h biomarker and in combination with miR-518b.

### Function and functional relationship analysis of target genes of differentially expressed extracellular C19MC microRNAs in early pregnancy in patients destined to develop gestational hypertension

The function and functional relationship analysis of predicted targets of up-regulated extracellular C19MC microRNAs in patients who subsequently developed gestational hypertension indicated an extensive group of pregnancy-related genes (miR-516-5p: 53 out of 349; miR-517^*^: 21 out of 179; miR-518b: 4 out of 42; miR-520h: 65 out of 509) (Table 4; S1–S4 Tables).

**Table 4:** Function and functional relationship analysis of target genes of differentially expressed extracellular C19MC microRNAs in patients developing gestational hypertension in relation to pregnancy.

microRNA	Total no. of predicted target genes/ no. of target genes with relation to pregnancy	Target genes with relation to pregnancy
**miR-516-5p**	349/53	GYS1, MAPK10, MSRB3, EGLN3, ITGA9, PMP22, CD177, PDGFRA, PSG5, LIN28B, TFCP2L1, PSG9, CHAF1B, IL17RE, PSG6, PSG2, DDAH1, KNG1, NOS1, FUT1, FLT1, UMPS, KCNQ3, SPRY2, PSG11, DAZ1, CHM, OBSL1, WARS, SPAST, SH3BGR, MEOX2, CD1A, PSG3, CCNG1, STC1, CCNA2, CCR2, EGR1, SOCS2, ICOSLG, KCNB1, SLC1A1, SLC9A1, MTHFR , IRAK1, TYRO3, CELF4, ANXA4, MS4A1, ADAMTS14, GRB2, SLC6A2
miR-517*	179/21	RND3, CUL4B, SFRP4, SDHC, MTDH, FUT1, LHCGR, SDC4, FUT9, MBD2, ZCCHC10, CCNG1, DGKE, LMNB2, FAS, SH3BGRL2, CASP10, NFIB, TWIST1, GCLC, PAPPA
**miR-518b**	42/4	TOLLIP, SLCO4C1, PRDX6, CXADR
**miR-520h**	509/65	NOX4, VAV3, KLF12, FBN1, JAZF1, CAPN2, TRPC5, ANXA4, LAMP2, HIF1A, RND3, EIF4E, GNA14, PAK2, NR4A2, GBP6, TLR5, GCH1, PHF14, PRCP, SENP1, MYCN, TFAM, CALU, OCLN, CCNB1, EDNRA, SLC41A1, F3, CNR1, ITGAV, SDC2, E2F1, GLRX, CGA, RGS2, ADAMTS5, PRIM2, CDKN1A, PCK1, TGFB2, LIN28B, ABCA1, UNG, AHRR, CDKN2B, SLK, OLR1, NOD2, PKHD1, LRP8, S1PR3, ACP1, BCOR, PAFAH1B2, IRS1, PAH, GRIP1, DMD, GPLD1, NTRK2, FURIN, ANGPT1, TBX1, PTPN1

All the targets were predicted by a bioinformatics tool MirTarget2 using miRDB online database.

Several target genes were previously described as aberrantly expressed in various biological samples derived from patients with clinical symptoms of pregnancy-related complications such as gestational hypertension, preeclampsia (with or without intrauterine growth restriction), HELLP syndrome, fetal growth restriction and/or small for gestational age, gestational diabetes mellitus, spontaneous abortions, miscarriages, recurrent pregnancy loss and ectopic pregnancy (miR-516-5p: GYS1, PDGFRA, SP1, DDAH1, KNG1, NOS1, FLT1, KCNQ3, STC1, SOCS2, SLC9A1, MTHFR, GRB2 and SLC6A2; miR-517^*^:-CUL4B, SFRP4, SDC4, FAS, SH3BGRL2, CASP10 and PAPPA; miR-518b: TOLLIP and PRDX6; miR-520h: NOX4, VAV3, FBN1, LAMP2, HIF1A, GNA14, TLR5, PRCP, CALU, OCLN, EDNRA, SLC41A1, F3, CB1, ITGAV, SDC2, E2F1, GLRX, CDKN1A, TGFB2, LIN28B, ABCA1, OLR1, ACP1, PAFAH1B2, NTRK2 and ANGPT1) (S1–S4 Tables).

Some of predicted targets such as PAPPA, SP1 (PSG2, PSG3, PSG5, PSG6, PSG9, PSG11), LHCGR, FLT1, ANGPT1 have been shown to be potential non-invasive early biomarkers for pregnancy-related complications such as gestational hypertension, preeclampsia, small for gestational age, miscarriage, preterm delivery, stillbirth and aneuploid foetuses (S1, S2, S4 Tables).

## Discussion

Although individual maternal plasma/serum markers have not usually performed well as a screening test for preeclampsia [50], [51], [53]–[69] and fetal growth restriction [52], [57], [58], combined screening tests to assess the risk of preeclampsia are currently used in practice [66]. In a proposed new approach to prenatal care, screening by a combination of maternal risk factors, mean arterial pressure, uterine artery Doppler and maternal serum biomarkers (pregnancy-associated plasma protein-A and placental growth factor) can identify about 95% of cases with early onset of preeclampsia for a false-positive rate of 10% [66].

Further research is needed to discover other biomarkers with better diagnostic performance in order to improve the prediction of placental-insufficiency related complications. Recent study of Luque et al. demonstrated no predictive value of first trimester maternal serum miRNA assessment for early preeclampsia analysing a total of 754 miRNAs [70]. Initially, miRNA profiling on high-throughput OpenArray™ system revealed differential abundance profile of 7 microRNAs in early preeclampsia (miR-192, miR-143 and miR-125b were overrepresented and miR-127, miR-942, miR-126 and miR-221 were underrepresented in preeclampsia). Consequently, validation by real-time quantitative stem-loop RT-PCR analysis revealed no significant differences between preeclampsia and controls.

On the other hand, Winger et al. were able to predict with great accuracy miscarriage and late preeclampsia during the first trimester of pregnancy via screening of 30 non-placental microRNAs (miR-340-5p, miR-424-5p, miR-33a-5p, miR-7-5p, miR-1229, miR-1267, miR-671-3p, miR-1, miR-133b, miR-144-3p, miR-582-5p, miR-30e-3p, miR-199a-5p, miR-199b-5p, miR-210, miR-221-5p, miR-575, miR-301a-3p, miR-148a-3p, miR-193a-3p, miR-219-5p, miR-132, miR-513a-5p, miR-1244, miR-16, miR-146a, miR-155, miR-181a, miR-196a and miR-223) in maternal peripheral blood mononuclear cells using quantitative RT-PCR [71]. Results for each microRNA were arranged from highest to lowest Ct value, scored using a devised scoring system giving points to each patient sample where the result of microRNA quantification fell within the topmost eight results. Finally, the results of all microRNAs were summed for each patient and individual pregnancy risk score was assessed. Four microRNAs (miR-33a-5p, miR-219-5p, miR-424-5p and miR-513-5p) demonstrated very low readings so were considered technically unsuitable for analysis and excluded from the scoring system.

Similarly, Ura et al. identified 19 differentially expressed mature miRNAs including 12 upregulated (miR-1233, miR-650, miR-520a, miR-215, miR-210, miR-25, miR-518b, miR-193a-3p, miR-32, miR-204, miR-296-5p and miR-152) and 7 dowregulated (miR-126, miR-335, miR-144, miR-204, miR-668, miR-376a and miR-15b) at early stages of gestation in the serum of pregnant women, who later developed severe preeclampsia using microarray analysis and subsequently validated the expression of 4 miRNAs (miR-1233, miR-520a, miR-210 and miR-144) using quantitative real-time PCR (72). Mir-1233 was the most overexpressed (5.6 fold change), mir-520a showed a 3.5 fold-increase, miR-210 a 3.3 fold-increase and miR-155 a 0.4 fold-decrease in the serum of women who later developed severe preeclampsia.

The results of our previous pilot study strongly supported the need for a more detailed exploration of extracellular placental specific C19MC microRNAs in maternal circulation with the view toward their recognition as potential biomarkers for placental insufficiency related complications (46, 47). Using both the absolute and relative quantification approaches, the ability of extracellular C19MC microRNAs (miR-516-5p, miR-517*, miR-518b, miR-520a*, miR-520h, miR-525 and miR-526a) to differentiate between normal pregnancies and all women destined to develop preeclampsia and/or intrauterine growth restriction in early pregnancy (between the 12^th^ and 16^th^ weeks of gestation) was outlined. This pilot study included 6 pregnant women altogether (1 early preeclampsia, 4 late preeclampsia and 1 early IUGR) [46]. Ura et al. significantly contributed to the confirmation of the results of our pilot study when reported up-regulation of placental specific miR-520a in sera from 12–14 week-gestation in the group of women who later developed severe preeclampsia [72]. Mir-520a (miR-520a-3p) is derived from miR-520a stem-loop together with miR-520a* (miR-520a-5p), which was a subject of interest in our study (http://mirdb.org). Similarly, as Ura et al. we observed in our pilot study upregulation of circulating miR-517* in early pregnancy destined to develop preeclampsia, but this finding was not confirmed in validation experiments performed by Ura et al. [72]. Concerning the selection of microRNAs that were evaluated as early biomarkers of pregnancy-related complications, there is no overlap between our pilot study and studies performed by Luque et al. and Winger et al. [46], [47], [70], [71].

Consecutive large scale studies are needed to assess sensitivity, specificity and positive predictive value of C19MC microRNAs for preeclampsia and/or intrauterine growth restriction. In addition, the diagnostic performance of placental specific microRNAs in relation to the severity of the disease with respect to clinical signs, requirements for the delivery and Doppler ultrasound parameters should be evaluated.

To our knowledge, this is the first longitudinal cohort study in an unselected population reported to date evaluated risk assessment for gestational hypertension, based on maternal plasma concentrations of placental specific C19MC microRNAs in early pregnancy. The study brought interesting finding that the up-regulation of circulating C19MC microRNAs (miR-520h, miR-518b, miR-516-5p, and miR-517*) is a characteristic phenomenon of early pregnancy destined to develop not only placenta-insufficiency related complications, but gestational hypertension as well. In addition, the presence of first trimester higher plasmatic levels of miR-520h, miR-518b and miR-516-5p alone certainly appears to be predictive of subsequent gestational hypertension. Effective screening for onset of gestational hypertension can be achieved in the first-trimester of pregnancy using a single C19MC placental specific microRNA biomarker (miR-520h). Alternatively, the combination of 2 placental specific C19MC microRNA biomarkers (miR-520h and miR-518b) may be used to predict the occurrence of gestational hypertension. Consecutive multi-centre large scale studies involving the patients from all populations are needed to verify that a single plasmatic miR-520h biomarker or a combination of miR-520h and miR-518b biomarkers represent promising tools in the risk assessment for gestational hypertension. The increased levels of extracellular C19MC microRNAs during the first trimester of gestation may be related to down-regulation of some proteins and hormones that have been studied as potential early markers for gestational hypertension, preeclampsia, SGA, preterm delivery, miscarriages, stillbirth or Dowńs syndrome (e.g. pregnancy-specific glycoproteins (SP1), PAPP-A, LHCGR (Table 4, S1, S2 Tables).

However, none has achieved sufficiently good discrimination to be used alone in a clinical context, although combinations of second trimester biochemical markers and biochemical and ultrasound markers have been proposed. On the other hand, some predicted targets of C19MC microRNAs, that were subject of interest in the current study, were shown previously to be upregulated in first trimester maternal plasma/serum samples derived from patients with preeclampsia, SGA, preterm delivery, miscarriage or stillbirth (e.g. soluble Flt-1, Ang-1/Ang-2 ratio) (Table 4, S1, S4 Tables).

Nevertheless, multiple miRNAs can regulate a single gene. Although methods to comprehensively identify miRNAs that regulate individual genes of interest are currently available, pathways involving miRNAs are often complex regulatory networks, whose regulation is difficult to understand and make the direct interpretation of experimental data elaborate. Many genes are targeted for repression by a high number of miRNAs, which seem to regulate those genes cooperatively [73].

In conclusion, microRNAs play a fundamental role in a variety of physiological and pathological processes involving pregnancy-related complications. Current study demonstrated for the first time that circulating C19MC microRNAs might play a role in early pregnancy in the inducement of not only preeclampsia and fetal growth restriction, but gestational hypertension as well.

## Supporting Information

S1 Table **Function of target genes of miR-516-5p (miR-516b-5p) in relation to pregnancy.**
(DOCX)

S2 Table Function of target genes of miR-517* (miR-517-5p) in relation to pregnancy.
(DOC)

S3 Table **Function of target genes of miR-518b in relation to pregnancy.**
(DOCX)

S4 Table **Function of target genes of miR-520h in relation to pregnancy.**
(DOCX)
