# Supplementary material for: First Trimester Screening of Circulating C19MC microRNAs Can Predict Subsequent Onset of Gestational Hypertension
Source: PLoS One. 2014 Dec 15;9(12):e113735. doi: 10.1371/journal.pone.0113735 (PMC4266496; doi:10.1371/journal.pone.0113735)
Supplement: S1 Table — Function of target genes of miR-516-5p (miR-516b-5p) in relation to pregnancy. (DOCX) [file pone.0113735.s001.docx]

**Table S1. Function of target genes of differentially expressed extracellular C19MC microRNAs in patients developing gestational hypertension in relation to pregnancy**

**miR-516-5p (miR-516b-5p)**

| **No.** | **GENE** | **GENE full name** | **Total number of references,** | **The role in gestation** |
| --- | --- | --- | --- | --- |
|  | **official symbol** |  | **list of references in PubMed (humans)** |  |
| 1 | GYS1 | glycogen synthase 1 (muscle) | 3 [1-3] | During an analysis of the oligosaccharide content of syncytiotrophoblast microvesicles purified from the placental chorionic villi of primigravid women with proteinuric pre-eclampsia, an excess of glycogen breakdown products was found [1]. |
|  |  |  |  | The glycogen content of placental tissue of normal pregnancy decreased significantly with increased gestational age. The decrease in placental glycogen content was accompanied by a corresponding decrease in the placental glycogensynthetase enzyme levels [2]. |
|  |  |  |  | In myometrium of pregnant women with gestational (treated and untreated) diabetes GS activity was decreased [3]. |
| 2 | MAPK10 | mitogen-activated protein kinase 10 | 1 [4] | Chromosome 4q deletion syndrome (4q- syndrome) is a rare condition, with an estimated incidence of 1 in 100,000. Significant genotype-phenotype correlations at a single gene level linking specific phenotypes to individual genes inclusive MAPK10 were found [4]. |
| 3 | C6orf174 | chromosome 6 open reading frame 174 | No results in PubMed | none |
| 4 | GABRB2 | gamma-aminobutyric acid (GABA) A receptor, beta 2 | No results in PubMed | none |
| 5 | FCER2 | Fc fragment of IgE, low affinity II, receptor for (CD23) | No results in PubMed | none |
| 6 | APOLD1 | apolipoprotein L domain containing 1 | No results in PubMed | none |
| 7 | AAK1 | AP2 associated kinase 1 | No results in PubMed | none |
| 8 | PPP1R12B | protein phosphatase 1, regulatory subunit 12B | No results in PubMed | none |
| 9 | LOC100294033 | protein FAM115A-like | No results in PubMed | none |
| 10 | MSRB3 | methionine sulfoxide reductase B3 | 1 [5] | Differentially methylated regions associated with gestational age at birth were identified in MSRB3 gene, which plays important role in the development of several organs, including skeletal muscle, brain and haematopoietic system. Therefore, MSRB3 gene may provide initial insight into the basis of preterm births negative health outcomes [5]. |
| 11 | FAM115A | family with sequence similarity 115, member A | No results in PubMed | none |
| 12 | MAP7D1 | MAP7 domain containing 1 | No results in PubMed | none |
| 13 | EGLN3 | egl nine homolog 3 (C. elegans) | 1 [6] | EGLN3 is temporally expressed in an oxygen-dependent fashion, with greatest mRNA expression at 10-12 week of gestation. Inhibition of EGLN activity increases HIF1A stability in villous explants and stimulates transforming growth factor beta 3 (TGFB3) expression consistent with promoter analyses showing that HIF1A transactivates TGFB3. These data demonstrate that during placental development, HIF1A is regulated by temporal and spatial changes in expression and association of molecules forming the multi-protein VHLCBC complex as well as prolyl hydroxylase activities [6]. |
| 14 | GXYLT1 | glucoside xylosyltransferase 1 | No results in PubMed | none |
| 15 | RMND5A | required for meiotic nuclear division 5 homolog A (S. cerevisiae) | No results in PubMed | none |
| 16 | NPEPPS | aminopeptidase puromycin sensitive | No results in PubMed | none |
| 17 | KIAA0408 | KIAA0408 | No results in PubMed | none |
| 18 | SPAG16 | sperm associated antigen 16 | No results in PubMed | none |
| 19 | FAM107A | family with sequence similarity 107, member A | No results in PubMed | none |
| 20 | SLC13A3 | solute carrier family 13 (sodium-dependent dicarboxylate transporter), member 3 | No results in PubMed | none |
| 21 | RUNDC3B | RUN domain containing 3B | No results in PubMed | none |
| 22 | CACNB2 | calcium channel, voltage-dependent, beta 2 subunit | No results in PubMed | none |
| 23 | SAMD12 | sterile alpha motif domain containing 12 | No results in PubMed | none |
| 24 | ATP2A2 | ATPase, Ca++ transporting, cardiac muscle, slow twitch 2 | No results in PubMed | none |
| 25 | ITGA9 | integrin, alpha 9 | 1 [7] | The possible link between ITGA9 and severe congenital chylothorax in human foetuses is discussed [7]. |
| 26 | PCDH7 | protocadherin 7 | No results in PubMed | none |
| 27 | TOX3 | TOX high mobility group box family member 3 | No results in PubMed | none |
| 28 | INO80D | INO80 complex subunit D | No results in PubMed | none |
| 29 | SYT9 | synaptotagmin IX | No results in PubMed | none |
| 30 | PMP22 | peripheral myelin protein 22 | 59 [selection 8,9] | Charcot-Marie-Tooth syndrome is the most common hereditary peripheral neuropathy, with an incidence of about 1 in 2500. The subtype 1A (CMT1A) is caused by a tandem duplication of a 1.5-Mb region encompassing the PMP22 gene [8, 9]. |
| 31 | DDHD1 | DDHD domain containing 1 | No results in PubMed | none |
| 32 | LOC100653123 | uncharacterized LOC100653123 | No results in PubMed | none |
| 33 | HP11026 | uncharacterized protein HP11026 | No results in PubMed | none |
| 34 | ADH5 | alcohol dehydrogenase 5 (class III), chi polypeptide | No results in PubMed | none |
| 35 | SNAPC3 | small nuclear RNA activating complex, polypeptide 3, 50kDa | No results in PubMed | none |
| 36 | SDK2 | sidekick homolog 2 (chicken) | No results in PubMed | none |
| 37 | WDR26 | WD repeat domain 26 | No results in PubMed | none |
| 38 | PPP1R3B | protein phosphatase 1, regulatory subunit 3B | No results in PubMed | none |
| 39 | C7orf10 | chromosome 7 open reading frame 10 | No results in PubMed | none |
| 40 | ADAL | adenosine deaminase-like | No results in PubMed | none |
| 41 | ILDR2 | immunoglobulin-like domain containing receptor 2 | No results in PubMed | none |
| 42 | ING3 | inhibitor of growth family, member 3 | No results in PubMed | none |
| 43 | OLFML1 | olfactomedin-like 1 | No results in PubMed | none |
| 44 | CSRNP3 | cysteine-serine-rich nuclear protein 3 | No results in PubMed | none |
| 45 | MON2 | MON2 homolog (S. cerevisiae) | No results in PubMed | none |
| 46 | MRPS35 | mitochondrial ribosomal protein S35 | No results in PubMed | none |
| 47 | TMEM183A | transmembrane protein 183A | No results in PubMed | none |
| 48 | CD177 | CD177 molecule, Neutrophil-specific antigen HNA-2a | 1 [10] | HNA-2a antigen expression increases in pregnancy. Some of the variations in neutrophil expression of HNA-2a among individuals are likely due to differences in gene regulation or differences in post-translational protein modifications rather than gene polymorphisms [10]. |
| 49 | ZNF548 | zinc finger protein 548 | No results in PubMed | none |
| 50 | ZFAND5 | zinc finger, AN1-type domain 5 | No results in PubMed | none |
| **51** | **PDGFRA** | **platelet-derived growth factor receptor, alpha polypeptide** | **3 [11-13]** | Altered expression of angiogenesis-related placental genes inclusive of PDGFRA gene in pre-eclampsia is associated with intrauterine growth restriction [11]. |
|  |  |  |  | The term placenta samples of the gestational hypertension group and gestational diabetes group displayed a significantly stronger PDGF-R mRNA signal [12]. |
|  |  |  |  | PDGF-AA and the alpha receptor were present in both normal and preeclamptic placentas. Immunoreactive staining revealed PDGF-AA and its receptor in the intimal/endothelial layer of fetal vessels and the trophoblastic layer. Staining intensity was greater in preeclamptic tissue when obliterative endarteritis was present [13]. |
| 79 | **PSG2** | **pregnancy specific beta-1-glycoprotein 2** | 14 [selection 14-18] | The human pregnancy-specific glycoproteins (PSGs) are a group of molecules that are mainly produced by the placental syncytiotrophoblasts during pregnancy. PSGs comprise a subgroup of the carcinoembryonic antigen (CEA) family, which belongs to the immunoglobulin superfamily. SP1 is a collective name for a group of pregnancy-specific glycoproteins (PSG) of varying size transcribed from a family of 11 genes [14]. |
| 212 | **PSG3** | **pregnancy specific beta-1-glycoprotein 3** |  |  |
| 52 | **PSG5** | **pregnancy specific beta-1-glycoprotein 5** |  |  |
| 73 | **PSG6** | **pregnancy specific beta-1-glycoprotein 6** |  |  |
| 63 | **PSG9** | **pregnancy specific beta-1-glycoprotein 9** |  | SP1 was identified as a first trimester maternal serum marker of SGA (30% DR for fetuses <2.5^th^ percentile at 10% FPR) and preterm delivery (24% DR at 10% FPR) [15]. |
| 168 | **PSG11** | **pregnancy specific beta-1-glycoprotein 11** |  |  |
|  |  |  |  | The mRNA expression of pregnancy-specific beta1 glycoprotein and trophoblast glycoprotein is up-regulated in cells circulating within blood from women with preeclampsia, and pregnancy-specific beta1 glycoprotein expression is positively correlated with the clinical severity of preeclampsia [16]. |
|  |  |  |  | Two additional PSG cDNAs, PSG9 and PSG10, whose transcripts are largely expressed in placental tissue and to a lesser extent in some other cell types, were isolated and characterized [17]. |
|  |  |  |  | Genome-wide association study identifies a maternal copy-number deletion in PSG11 enriched among preeclampsia patients [18]. |
| 53 | LIN28B | lin-28 homolog B (C. elegans) | 3 [selection 19,20] | LIN28B is a transcription factor associated with the fetal lymphoid lineages [19]. |
|  |  |  |  | Seven new imprinted genes expressed in the human placenta, including ZFAT, ZFAT-AS1, GLIS3, NTM, MAGI2, ZC3H12C and LIN28B were identified [20]. |
| 54 | TFCP2L1 | transcription factor CP2-like 1 (LBP-9) | 1 [21] | LBP-1b, LBP-9, and LBP-32/MGR were detected in syncytiotrophoblasts from first-trimester human placental tissue [21]. |
| 55 | LYPD5 | LY6/PLAUR domain containing 5 | No results in PubMed | none |
| 56 | TUBB | tubulin, beta class I | No results in PubMed | none |
| 57 | PIP5K1A | phosphatidylinositol-4-phosphate 5-kinase, type I, alpha | No results in PubMed | none |
| 58 | CEP350 | centrosomal protein 350kDa | No results in PubMed | none |
| 59 | DDX3X | DEAD (Asp-Glu-Ala-Asp) box polypeptide 3, X-linked | No results in PubMed | none |
| 60 | TATDN3 | TatD DNase domain containing 3 | No results in PubMed | none |
| 61 | PDK3 | pyruvate dehydrogenase kinase, isozyme 3 | No results in PubMed | none |
| 62 | ZFP14 | zinc finger protein 14 homolog (mouse) | No results in PubMed | none |
| 64 | GGPS1 | geranylgeranyl diphosphate synthase 1 | No results in PubMed | none |
| 65 | RIC3 | resistance to inhibitors of cholinesterase 3 homolog (C. elegans) | No results in PubMed | none |
| 66 | ZIC1 | Zic family member 1 | No results in PubMed | none |
| 67 | CHAF1B | chromatin assembly factor 1, subunit B (p60) | 1 [22] | The localisation of the CAF1P60 gene to human chromosome 21 and its fine mapping to 21q22.2 between D21S333 and D21S334 is demonstrated. This mapping position places CAF1P60 in a region of HSA21 which is strongly associated with the major features of Down syndrome [22]. |
| 68 | TMTC1 | transmembrane and tetratricopeptide repeat containing 1 | No results in PubMed | none |
| 69 | LARS2 | leucyl-tRNA synthetase 2, mitochondrial | No results in PubMed | none |
| 70 | IQSEC3 | IQ motif and Sec7 domain 3 | No results in PubMed | none |
| 71 | IL17RE | interleukin 17 receptor E | 1 [23] | The ratio of Foxp3(+) Treg to IL-17-expressing CD4(+) T cells was significantly increased in healthy but not in preeclamptic pregnancies [23]. |
| 72 | RBM18 | RNA binding motif protein 18 | No results in PubMed | none |
| 74 | HECTD2 | HECT domain containing 2 | No results in PubMed | none |
| 75 | CPOX | coproporphyrinogen oxidase | No results in PubMed | none |
| 76 | XIRP2 | xin actin-binding repeat containing 2 | No results in PubMed | none |
| 77 | NAP1L1 | nucleosome assembly protein 1-like 1 | No results in PubMed | none |
| 78 | HBS1L | HBS1-like (S. cerevisiae) | No results in PubMed | none |
| 80 | DDAH1 | dimethylarginine dimethylaminohydrolase 1 | 9 [selection 24-26] | Decreased DDAH activity in the preeclamptic placenta might contribute to elevated asymmetric dimethylarginine levels in these patients [24]. Haplotypic association of DDAH1 with susceptibility to pre-eclampsia was described [25, 26]. |
| 81 | GIPC3 | GIPC PDZ domain containing family, member 3 | No results in PubMed | none |
| 82 | TMOD1 | tropomodulin 1 | No results in PubMed | none |
| 83 | ZNF250 | zinc finger protein 250 | No results in PubMed | none |
| 84 | GRIA4 | glutamate receptor, ionotrophic, AMPA 4 | No results in PubMed | none |
| 85 | C7orf57 | chromosome 7 open reading frame 57 | No results in PubMed | none |
| 86 | PJA2 | praja ring finger 2 | No results in PubMed | none |
| 87 | HS6ST3 | heparan sulfate 6-O-sulfotransferase 3 | No results in PubMed | none |
| 88 | ITIH5 | inter-alpha-trypsin inhibitor heavy chain family, member 5 | No results in PubMed | none |
| 89 | RNF122 | ring finger protein 122 | No results in PubMed | none |
| 90 | LOC100653112 | heparan-sulfate 6-O-sulfotransferase 3-like | No results in PubMed | none |
| 91 | C20orf7 | chromosome 20 open reading frame 7 | No results in PubMed | none |
| 92 | PIK3R5 | phosphoinositide-3-kinase, regulatory subunit 5 | No results in PubMed | none |
| 93 | PAFAH1B2 | platelet-activating factor acetylhydrolase 1b, catalytic subunit 2 (30kDa) | No results in PubMed | none |
| 94 | SYNPR | synaptoporin | No results in PubMed | none |
| 95 | CD109 | CD109 molecule | No results in PubMed | none |
| 96 | KNG1 | kininogen 1 | 34 [selection 27,28] | Activation of the intrinsic coagulation pathway may be operative in the genesis of disseminated intravascular coagulation in pre-eclampsia [27]. |
|  |  |  |  | Vitronectin and high-molecular-weight kininogen may prove to be useful as early markers of fibrinolytic activity and neutrophil activation, which are known to be associated with preeclampsia [28]. |
| 97 | IQSEC1 | IQ motif and Sec7 domain 1 | No results in PubMed | none |
| 98 | ANGPTL1 | angiopoietin-like 1 | No results in PubMed | none |
| 99 | UNC80 | unc-80 homolog (C. elegans) | No results in PubMed | none |
| 100 | C9orf100 | chromosome 9 open reading frame 100 | No results in PubMed | none |
| 101 | PLAGL2 | pleiomorphic adenoma gene-like 2 | No results in PubMed | none |
| 102 | WDR52 | WD repeat domain 52 | No results in PubMed | none |
| 103 | TMPRSS11D | transmembrane protease, serine 11D | No results in PubMed | none |
| 104 | TSPAN12 | tetraspanin 12 | No results in PubMed | none |
| 105 | APOL6 | apolipoprotein L, 6 | No results in PubMed | none |
| 106 | HECW1 | HECT, C2 and WW domain containing E3 ubiquitin protein ligase 1 | No results in PubMed | none |
| 107 | ABCD4 | ATP-binding cassette, sub-family D (ALD), member 4 | No results in PubMed | none |
| 108 | ANKH | ankylosis, progressive homolog (mouse) | No results in PubMed | none |
| 109 | ZNF626 | zinc finger protein 626 | No results in PubMed | none |
| 110 | C20orf203 | chromosome 20 open reading frame 203 | No results in PubMed | none |
| 111 | ZNF471 | zinc finger protein 471 | No results in PubMed | none |
| 112 | TP53INP1 | tumor protein p53 inducible nuclear protein 1 | No results in PubMed | none |
| 113 | STOM | stomatin | No results in PubMed | none |
| 114 | PCDH9 | protocadherin 9 | No results in PubMed | none |
| 115 | OLA1 | Obg-like ATPase 1 | No results in PubMed | none |
| 116 | RANBP6 | RAN binding protein 6 | No results in PubMed | none |
| 117 | NOS1 | nitric oxide synthase 1 (neuronal) | 6 [selection 29-30] | Preeclampsia is associated with loss of neuronal nitric oxide synthase expression in vascular smooth muscle cells of the human umbilical cord [29]. |
|  |  |  |  | The significant increase of the eNOS mRNA expression, from the unripe to the favourable cervical state during labour, may indicate a role of eNOS and supports the role of NO in the cervical ripening process [30]. |
| 118 | STK17A | serine/threonine kinase 17a | No results in PubMed | none |
| 119 | TMEM25 | transmembrane protein 25 | No results in PubMed | none |
| 120 | GRIA2 | glutamate receptor, ionotropic, AMPA 2 | No results in PubMed | none |
| 121 | FUT1 | fucosyltransferase 1 (galactoside 2-alpha-L-fucosyltransferase, H blood group) | 2 [31,32] | Macrophage-derived factors including LIF might facilitate development of an implantation-receptive endometrium by regulating surface glycan structures in epithelial cells [31]. |
|  |  |  |  | In trisomy 21 cultured trophoblasts, transcripts of sialyltransferase-1 and fucosyltransferase-1 were abnormally high. Trisomy 21 trophoblast cells produced hCG that was weakly bioactive and abnormally glycosylated but whose maternal clearance was unaltered [32]. |
| 122 | PNPT1 | polyribonucleotide nucleotidyltransferase 1 | No results in PubMed | none |
| 123 | EXTL2 | exostoses (multiple)-like 2 | No results in PubMed | none |
| 124 | FAM86A | family with sequence similarity 86, member A | No results in PubMed | none |
| 125 | LMBR1 | limb region 1 homolog (mouse) | No results in PubMed | none |
| 126 | SEL1L | sel-1 suppressor of lin-12-like (C. elegans) | No results in PubMed | none |
| 127 | COX18 | COX18 cytochrome c oxidase assembly homolog (S. cerevisiae) | No results in PubMed | none |
| 128 | GPR107 | G protein-coupled receptor 107 | No results in PubMed | none |
| 129 | ZNFX1 | zinc finger, NFX1-type containing 1 | No results in PubMed | none |
| 130 | UBN2 | ubinuclein 2 | No results in PubMed | none |
| 131 | POLR3F | polymerase (RNA) III (DNA directed) polypeptide F, 39 kDa | No results in PubMed | none |
| 132 | ARHGAP5 | Rho GTPase activating protein 5 | No results in PubMed | none |
| 133 | TRIM2 | tripartite motif containing 2 | No results in PubMed | none |
| 134 | VGLL2 | vestigial like 2 (Drosophila) | No results in PubMed | none |
| 135 | PERP | PERP, TP53 apoptosis effector | No results in PubMed | none |
| 136 | WDR93 | WD repeat domain 93 | No results in PubMed | none |
| 137 | GALNT10 | UDP-N-acetyl-alpha-D-galactosamine:polypeptide N-acetylgalactosaminyltransferase 10 (GalNAc-T10) | No results in PubMed | none |
| 138 | PDCD6IP | programmed cell death 6 interacting protein | No results in PubMed | none |
| 139 | OSBPL3 | oxysterol binding protein-like 3 | No results in PubMed | none |
| 140 | ANKRD43 | ankyrin repeat domain 43 | No results in PubMed | none |
| 141 | C15orf23 | chromosome 15 open reading frame 23 | No results in PubMed | none |
| 142 | RALY | RNA binding protein, autoantigenic (hnRNP-associated with lethal yellow homolog (mouse)) | No results in PubMed | none |
| 143 | SP7 | Sp7 transcription factor | No results in PubMed | none |
| 144 | SLC36A3 | solute carrier family 36 (proton/amino acid symporter), member 3 | No results in PubMed | none |
| 145 | COPS2 | COP9 constitutive photomorphogenic homolog subunit 2 (Arabidopsis) | No results in PubMed | none |
| 146 | TPCN1 | two pore segment channel 1 | No results in PubMed | none |
| 147 | TMEM39A | transmembrane protein 39A | No results in PubMed | none |
| **148** | **FLT1** | **fms-related tyrosine kinase 1 (vascular endothelial growth factor/vascular permeability factor receptor)** | **383 [selection 33,34]** | A member of vascular endothelial growth factor receptor (VEGFR-1) playing an important role in angiogenesis and vasculogenesis [33]. **Soluble Flt-1 has a 93.5% predictive capacity for preeclampsia at 6-15 weeks when combined with PIGF and soluble endoglin (PIGF/sVEGFR-1 + sEng screening), although the specificity and positive predictive value is low (31% and 5.1%) [34].** |
| 149 | DAZL | deleted in azoospermia-like | No results in PubMed | none |
| 150 | LOC100506255 | uncharacterized LOC100506255 | No results in PubMed | none |
| 151 | IKZF3 | IKAROS family zinc finger 3 (Aiolos) | No results in PubMed | none |
| 152 | SYT14 | synaptotagmin XIV | No results in PubMed | none |
| 153 | HSDL2 | hydroxysteroid dehydrogenase like 2 | No results in PubMed | none |
| 154 | HIPK2 | homeodomain interacting protein kinase 2 | No results in PubMed | none |
| 155 | PDHA1 | pyruvate dehydrogenase (lipoamide) alpha 1 | No results in PubMed | none |
| 156 | UMPS | uridine monophosphate synthetase | 1 [35] | HPLC assay of uridine monophosphate synthase confirmed its presence in chorionic villus samples and erythrocytes [35]. |
| 157 | NSL1 | NSL1, MIND kinetochore complex component, homolog (S. cerevisiae) | No results in PubMed | none |
| 158 | CSAG1 | chondrosarcoma associated gene 1 | No results in PubMed | none |
| 159 | DERL2 | Der1-like domain family, member 2 | No results in PubMed | none |
| 160 | C15orf32 | chromosome 15 open reading frame 32 | No results in PubMed | none |
| 161 | KCNQ3 | potassium voltage-gated channel, KQT-like subfamily, member 3 | 1 [36] | KCNQ3 and KCNE5 mRNA placental expressions were significantly upregulated in preeclampsia versus controls and exhibited a strong positive correlation with each other suggesting a novel heterodimer [36]. |
| 162 | RBAK | RB-associated KRAB zinc finger | No results in PubMed | none |
| 163 | THAP6 | THAP domain containing 6 | No results in PubMed | none |
| 164 | SPRY2 | sprouty homolog 2 (Drosophila) | 2 [37,38] | Human placental Hofbauer cells express sprouty proteins: a possible modulating mechanism of villous branching [37]. |
|  |  |  |  | FGF 10 and Sprouty 2 modulate trophoblast invasion and branching morphogenesis [38]. |
| 165 | GPR26 | G protein-coupled receptor 26 | No results in PubMed | none |
| 166 | ABHD5 | abhydrolase domain containing 5 | No results in PubMed | none |
| 167 | ZNF778 | zinc finger protein 778 | No results in PubMed | none |
| 169 | SMC2 | structural maintenance of chromosomes 2 | No results in PubMed | none |
| 170 | DAZ1 | deleted in azoospermia 1 | 3 [selection 39] | A new Y chromosome marker for noninvasive fetal gender determination [39]. |
| 171 | CHM | choroideremia (Rab escort protein 1) | 1 [40] | Choroideremia is a slowly progressive X-linked retinal degeneration that results in a loss of photoreceptors, retinal pigment epithelium and choroid. Mutations in the CHM gene are known to be associated with choroideremia, The prenatal diagnosis of duplication on the long arm of chromosome X from chromosomal band Xq13.2 to q21.31 in a male fetus with increased nuchal translucency in the first trimester and polyhydramnios at 22 weeks of gestation is reported [40]. |
| 172 | EVI5 | ecotropic viral integration site 5 | No results in PubMed | none |
| 173 | B3GAT3 | beta-1,3-glucuronyltransferase 3 (glucuronosyltransferase I) | No results in PubMed | none |
| 174 | RAP1B | RAP1B, member of RAS oncogene family | No results in PubMed | none |
| 175 | OBSL1 | obscurin-like 1 | 1 [41] | 3-M syndrome is an autosomal recessive primordial growth disorder characterized by small birth size and post-natal growth restriction associated with a spectrum of minor anomalies (including a triangular-shaped face, flat cheeks, full lips, short chest and prominent fleshy heels). Mutations in three genes CUL7, OBSL1 and CCDC8 have been shown to cause 3-M. Potentially damaging sequence variants in CUL7 and OBSL1 have been identified in idiopathic short stature, including those born small with failure of catch-up growth, signifying that the 3-M pathway could play a wider role in disordered growth [41]. |
| 176 | SIAH3 | seven in absentia homolog 3 (Drosophila) | No results in PubMed | none |
| 177 | DAZ2 | deleted in azoospermia 2 | No results in PubMed | none |
| 178 | DAZ3 | deleted in azoospermia 3 | No results in PubMed | none |
| 179 | WARS | tryptophanyl-tRNA synthetase | 2 [42,43] | Human placental tryptophanyl transfer ribonucleic acid synthetase was purified and characterized [42, 43]. |
| 180 | DAZ4 | deleted in azoospermia 4 | No results in PubMed | none |
| 181 | MTRF1L | mitochondrial translational release factor 1-like | No results in PubMed | none |
| 182 | CAB39 | calcium binding protein 39 | No results in PubMed | none |
| 183 | NIPAL1 | NIPA-like domain containing 1 | No results in PubMed | none |
| 184 | FBXO10 | F-box protein 10 | No results in PubMed | none |
| 185 | UPK1B | uroplakin 1B | No results in PubMed | none |
| 186 | NSF | N-ethylmaleimide-sensitive factor | No results in PubMed | none |
| 187 | ZNF594 | zinc finger protein 594 | No results in PubMed | none |
| 188 | RYBP | RING1 and YY1 binding protein | No results in PubMed | none |
| 189 | WDR33 | WD repeat domain 33 | No results in PubMed | none |
| 190 | SPAST | spastin | 1 [44] | Spastic paraplegia type 4 (SPG4) is the most common autosomal dominant hereditary SPG caused by mutations in the SPAST gene. Prenatal diagnosis of autosomal dominant hereditary spastic paraplegia (SPG4) using direct mutation detection is reported [44]. |
| 191 | ARAP3 | ArfGAP with RhoGAP domain, ankyrin repeat and PH domain 3 | No results in PubMed | none |
| 192 | C9orf72 | chromosome 9 open reading frame 72 | No results in PubMed | none |
| 193 | A1CF | APOBEC1 complementation factor | No results in PubMed | none |
| 194 | SH3BGR | SH3 domain binding glutamic acid-rich protein | 2 [45,46] | The 5' promoter region of the human SH3BGR (SH3-Binding Glutamine Rich) gene located in the Down syndrome region-2, between markers D21S55 and MX1 of human chromosome 21, was isolated, mapped and sequenced [45, 46]. |
| 195 | MEOX2 | mesenchyme homeobox 2 (MOX2) | 5 [selection 47,48] | The homeobox genes MSX2 and MOX2 are candidates for regulating epithelial-mesenchymal cell interactions in the human placenta [47, 48]. |
| 196 | FAM123C | family with sequence similarity 123C | No results in PubMed | none |
| 197 | ZNF280D | zinc finger protein 280D | No results in PubMed | none |
| 198 | ATCAY | ataxia, cerebellar, Cayman type | No results in PubMed | none |
| 199 | KIAA1755 | KIAA1755 | No results in PubMed | none |
| 200 | SLC44A1 | solute carrier family 44, member 1 | No results in PubMed | none |
| 201 | FAM115C | family with sequence similarity 115, member C | No results in PubMed | none |
| 202 | CLIC4 | chloride intracellular channel 4 | No results in PubMed | none |
| 203 | TMEM213 | transmembrane protein 213 | No results in PubMed | none |
| 204 | TANC1 | tetratricopeptide repeat, ankyrin repeat and coiled-coil containing 1 | No results in PubMed | none |
| 205 | CBL | Cas-Br-M (murine) ecotropic retroviral transforming sequence | No results in PubMed | none |
| 206 | CD1A | CD1a molecule | 1 [49] | Decidual CD1a+ cells stimulated with TAG-72 decreased CD83 expression and diminished IL-15 and IFN-γ intracellular production [49]. |
| 207 | PLIN5 | perilipin 5 | No results in PubMed | none |
| 208 | DDB2 | damage-specific DNA binding protein 2, 48kDa | No results in PubMed | none |
| 209 | TMEM183B | transmembrane protein 183B | No results in PubMed | none |
| 210 | ZSWIM4 | zinc finger, SWIM-type containing 4 | No results in PubMed | none |
| 211 | RASSF8 | Ras association (RalGDS/AF-6) domain family (N-terminal) member 8 | No results in PubMed | none |
| 213 | ZC4H2 | zinc finger, C4H2 domain containing | No results in PubMed | none |
| 214 | TNMD | tenomodulin | No results in PubMed | none |
| 215 | CTC1 | CTS telomere maintenance complex component 1 | No results in PubMed | none |
| 216 | CCNG1 | cyclin G1 | 41 [selection 50] | Cyclins are proteins that support the progression of cell-cycle stages in proliferating cells. The presence and cellular localization of four G1 cyclins (D1, D2, D3 and E) were determined by immunohistochemistry. Cyclins D3 and E are important cell cycle regulatory proteins, and further, that cyclin E may function in trophoblast terminal differentiation as well [50]. |
| 217 | C2CD4A | C2 calcium-dependent domain containing 4A | No results in PubMed | none |
| 218 | RAPGEF6 | Rap guanine nucleotide exchange factor (GEF) 6 | No results in PubMed | none |
| 219 | BGLAP | bone gamma-carboxyglutamate (gla) protein | No results in PubMed | none |
| 220 | HNRNPH2 | heterogeneous nuclear ribonucleoprotein H2 (H') | No results in PubMed | none |
| 221 | MAP4K4 | mitogen-activated protein kinase kinase kinase kinase 4 | No results in PubMed | none |
| 222 | OSBPL11 | oxysterol binding protein-like 11 | No results in PubMed | none |
| 223 | FAM13B | family with sequence similarity 13, member B | No results in PubMed | none |
| 224 | KCTD6 | potassium channel tetramerisation domain containing 6 | No results in PubMed | none |
| 225 | GPR113 | G protein-coupled receptor 113 | No results in PubMed | none |
| 226 | NAALAD2 | N-acetylated alpha-linked acidic dipeptidase 2 | No results in PubMed | none |
| 227 | SULT1A2 | sulfotransferase family, cytosolic, 1A, phenol-preferring, member 2 | No results in PubMed | none |
| 228 | GRAMD1C | GRAM domain containing 1C | No results in PubMed | none |
| 229 | CMKLR1 | chemokine-like receptor 1 | No results in PubMed | none |
| 230 | MLLT11 | myeloid/lymphoid or mixed-lineage leukemia (trithorax homolog, Drosophila); translocated to, 11 | No results in PubMed | none |
| 231 | NHLRC3 | NHL repeat containing 3 | No results in PubMed | none |
| 232 | AMZ1 | archaelysin family metallopeptidase 1 | No results in PubMed | none |
| 233 | RELL1 | RELT-like 1 | No results in PubMed | none |
| 234 | ALDOB | aldolase B, fructose-bisphosphate | No results in PubMed | none |
| 235 | STC1 | stanniocalcin 1 | 4 [selection 51-53] | Only six genes including STC1 gene showed a homogeneous expression, and are probably involved in embryo implantation mechanisms. [51, 52]. |
|  |  |  |  | The discovery of elevated maternal plasma STC1 in pregnancy complications warrants further investigations of its potential as a biomarker [53]. |
| 236 | ST6GAL2 | ST6 beta-galactosamide alpha-2,6-sialyltranferase 2 | No results in PubMed | none |
| 237 | EXT2 | exostosin 2 | No results in PubMed | none |
| 238 | PM20D1 | peptidase M20 domain containing 1 | No results in PubMed | none |
| 239 | CCNA2 | cyclin A2 | 3 [54-56] | Cyclin A1 is not essential for early embryonic development and cyclinA2 only becomes essential for development beyond the stage of implantation [54]. |
|  |  |  |  | An important role of c-Src in the pathogenesis of gestational trophoblastic disease is documented. Inhibition of c-Src phosphorylation induced cell cycle arrest and reduced expressions of cyclin A2, cyclin B1, cyclin E1, FOXD3 and NANOG [55]. |
|  |  |  |  | 17beta-estradiol induced terminal differentiation in human trophoblastic cells, and that this event was estrogen-receptor-mediated. This process involved a loss in expression of Cyclins A2 and E, and a coincident increase in p27 (Kip1) [56]. |
| 240 | MCCC2 | methylcrotonoyl-CoA carboxylase 2 (beta) | No results in PubMed | none |
| 241 | KRT5 | keratin 5 | No results in PubMed | none |
| 242 | NRG4 | neuregulin 4 | No results in PubMed | none |
| 243 | NT5C2 | 5'-nucleotidase, cytosolic II | No results in PubMed | none |
| 244 | RBPMS | RNA binding protein with multiple splicing | No results in PubMed | none |
| 245 | FOXC1 | forkhead box C1 | No results in PubMed | none |
| 246 | RBM47 | RNA binding motif protein 47 | No results in PubMed | none |
| 247 | ZNF780B | zinc finger protein 780B | No results in PubMed | none |
| 248 | CCR2 | chemokine (C-C motif) receptor 2 | 23 [selection 57-59] | Thus, chemokines are expressed by maternal and embryonic cells during implantation, whereas corresponding receptors are on trophoblast cells. Promotion of trophoblast migration by chemokines and endometrial cell conditioned medium indicates an important involvement of chemokines in maternal-fetal communication [57]. |
|  |  |  |  | CCR2 gene variants might be associated with preeclampsia [58]. |
|  |  |  |  | Monocyte chemokine receptor expression and the chemokine milieu during pregnancy are tightly regulated to support pregnancy. The women with SLE responded similar to pregnancy as did healthy women with lower percentages of CCR2+, CCR5+ and CXCR3+ monocytes [59]. |
| 249 | CHST6 | carbohydrate (N-acetylglucosamine 6-O) sulfotransferase 6 | No results in PubMed | none |
| 250 | KIAA0196 | KIAA0196 | No results in PubMed | none |
| 251 | EGR1 | early growth response 1 | 18 [selection 60-62] | Early pregnancy peripheral blood global gene expression and preterm delivery risk was investigated. A set of genes including EGR1 achieved accurate prediagnostic separation of cases and controls [60]. |
|  |  |  |  | Resveratrol prevented the up-regulation of Egr-1 protein, a transcription factor necessary for induction of the vascular endothelial growth factor receptor-1 gene and caused up-regulation of heme oxygenase-1, a cytoprotective enzyme found to be dysfunctional in preeclampsia [61]. |
|  |  |  |  | Term placenta from obese women had both increased JNK and p38 signaling and greater EGR-1 protein relative to lean women. Lipotoxic insults induce inflammation in placental cells via activation of JNK/EGR-1 signaling [62]. |
| 252 | C15orf42 | chromosome 15 open reading frame 42 | No results in PubMed | none |
| 253 | SYF2 | SYF2 homolog, RNA splicing factor (S. cerevisiae) | No results in PubMed | none |
| 254 | EPHB1 | EPH receptor B1 | No results in PubMed | none |
| 255 | FCRLA | Fc receptor-like A | No results in PubMed | none |
| 256 | SOCS2 | suppressor of cytokine signaling 2 | 12 [selection 63-65] | The messenger RNA expression of suppressor of cytokine signaling-2 is up-regulated significantly in placental vascular disease [63]. |
|  |  |  |  | The up-regulation of SOCS2 and SOCS3 indicates these are the major negative regulators in umbilical placental microvessel endothelial cell activation pathways [64]. Total SOCS2 was also increased in IUGR placentas [65]. |
| 257 | ICOSLG | inducible T-cell co-stimulator ligand (B7H2) | 2 [66,67] | The immunomodulatory proteins B7-DC, B7-H2, and B7-H3 are differentially expressed across gestation in the human placenta [66]. |
|  |  |  |  | T-cell cytokine modulation by ICOS-B7H2 interactions is important in the delicate immune balance at the fetomaternal interface [67]. |
| 258 | CYP4V2 | cytochrome P450, family 4, subfamily V, polypeptide 2 | No results in PubMed | none |
| 259 | MKL2 | MKL/myocardin-like 2 | No results in PubMed | none |
| 260 | TRIB1 | tribbles homolog 1 (Drosophila) | No results in PubMed | none |
| 261 | FAT2 | FAT tumor suppressor homolog 2 (Drosophila) | No results in PubMed | none |
| 262 | ZNF124 | zinc finger protein 124 | No results in PubMed | none |
| 263 | GDAP2 | ganglioside induced differentiation associated protein 2 | No results in PubMed | none |
| 264 | KBTBD13 | kelch repeat and BTB (POZ) domain containing 13 | No results in PubMed | none |
| 265 | KCNB1 | potassium voltage-gated channel, Shab-related subfamily, member 1 | 1 [68] | Potassium K channels play an important role in controlling placental vascular function [68]. |
| 266 | SORBS1 | sorbin and SH3 domain containing 1 | No results in PubMed | none |
| 267 | DPAGT1 | dolichyl-phosphate (UDP-N-acetylglucosamine) N-acetylglucosaminephosphotransferase 1 (GlcNAc-1-P transferase) | No results in PubMed | none |
| 268 | ANP32E | acidic (leucine-rich) nuclear phosphoprotein 32 family, member E | No results in PubMed | none |
| 269 | HRH1 | histamine receptor H1 | No results in PubMed | none |
| 270 | STOML3 | stomatin (EPB72)-like 3 | No results in PubMed | none |
| 271 | PPL | periplakin | No results in PubMed | none |
| 272 | ACTN2 | actinin, alpha 2 | No results in PubMed | none |
| 273 | SLC1A1 | solute carrier family 1 (neuronal/epithelial high affinity glutamate transporter, system Xag), member 1; (EAAT3) | 1 [69] | The glutamate transporters EAAT1, EAAT2 and EAAT3 are key components of the glutamate-glutamine cycle and responsible for active transport of glutamate over the cell membrane. Specific localization of EAAT1, EAAT2 and EAAT3 in the human placenta during development is reported [69]. |
| 274 | ABLIM2 | actin binding LIM protein family, member 2 | No results in PubMed | none |
| 275 | CSAG2 | CSAG family, member 2 | No results in PubMed | none |
| 276 | CSAG3 | CSAG family, member 3 | No results in PubMed | none |
| 277 | C9orf47 | chromosome 9 open reading frame 47 | No results in PubMed | none |
| 278 | STK17B | serine/threonine kinase 17b | No results in PubMed | none |
| 279 | C15orf2 | chromosome 15 open reading frame 2 | No results in PubMed | none |
| 280 | SH2D5 | SH2 domain containing 5 | No results in PubMed | none |
| 281 | SLC9A1 | solute carrier family 9 (sodium/hydrogen exchanger), member 1; (NHE-1) | 8 [selection 70-72] | Suppression of NHE-1 might render the placenta with impaired uptake of water and electrolytes and therefore may be involved in the pathogenesis of preeclampsia [70]. |
|  |  |  |  | There is differential regulation of the activity and expression of Na(+)/H(+) exchanger isoforms in the microvillous plasma membrane over the course of gestation in normal pregnancy; this is not affected in pregnancies resulting in SGA babies at term [71]. |
|  |  |  |  | The reduced activity and expression of NHE in the microvillous plasma membrane of preterm IUGR placentas may compromise placental function and may contribute to the development of fetal acidosis in preterm IUGR foetuses [72]. |
| 282 | MXI1 | MAX interactor 1 | No results in PubMed | none |
| 283 | PTPRT | protein tyrosine phosphatase, receptor type, T | No results in PubMed | none |
| 284 | MTHFR | methylenetetrahydrofolate reductase (NAD(P)H) | 655 [selection 73] | Folate metabolism affects ovarian function, implantation, embryogenesis and the entire process of pregnancy. In addition to its well-established effect on the incidence of neural tube defects, associations have been found between reduced folic acid levels and increased homocysteine concentrations on the one hand, and recurrent spontaneous abortions and other complications of pregnancy on the other [73]. |
| 285 | RNF214 | ring finger protein 214 | No results in PubMed | none |
| 286 | AGAP1 | ArfGAP with GTPase domain, ankyrin repeat and PH domain 1 | No results in PubMed | none |
| 287 | SHQ1 | SHQ1 homolog (S. cerevisiae) | No results in PubMed | none |
| 288 | SLC39A14 | solute carrier family 39 (zinc transporter), member 14 | No results in PubMed | none |
| 289 | KLHL24 | kelch-like 24 (Drosophila) | No results in PubMed | none |
| 290 | WHSC1L1 | Wolf-Hirschhorn syndrome candidate 1-like 1 | No results in PubMed | none |
| 291 | HMGCS2 | 3-hydroxy-3-methylglutaryl-CoA synthase 2 (mitochondrial) | No results in PubMed | none |
| 292 | IRAK1 | interleukin-1 receptor-associated kinase 1 | 1 [74] | In maternal spleens, IRAK-3 and IRAK-1 are increased in response to intrauterine inflammation. Negative regulators of the maternal immune response may play an important role in protecting pregnancies from an exaggerated inflammatory response [74]. |
| 293 | BCDIN3D | BCDIN3 domain containing | No results in PubMed | none |
| 294 | TTI2 | TELO2 interacting protein 2 | No results in PubMed | none |
| 295 | SH2D4A | SH2 domain containing 4A | No results in PubMed | none |
| 296 | MS4A4A | membrane-spanning 4-domains, subfamily A, member 4 | No results in PubMed | none |
| 297 | KLHL31 | kelch-like 31 (Drosophila) | No results in PubMed | none |
| 298 | TYRO3 | TYRO3 protein tyrosine kinase | 1 [75] | Axl and Tyro3 modulate female reproduction by influencing gonadotropin-releasing hormone neuron survival and migration [75]. |
| 299 | CACNA2D4 | calcium channel, voltage-dependent, alpha 2/delta subunit 4 | No results in PubMed | none |
| 300 | CELF4 | CUGBP, Elav-like family member 4 | 1 [76] | The genotype-phenotype correlation and the consequence of haploinsufficiency of FBN2, DTNA and CELF4 in a pregnancy with fetal interrupted aortic arch and atrial septal defect are discussed [76]. |
| 301 | ZNF324 | zinc finger protein 324 | No results in PubMed | none |
| 302 | SRSF8 | serine/arginine-rich splicing factor 8 | No results in PubMed | none |
| 303 | ZNF434 | zinc finger protein 434 | No results in PubMed | none |
| 304 | NUP133 | nucleoporin 133kDa | No results in PubMed | none |
| 305 | SUMF2 | sulfatase modifying factor 2 | No results in PubMed | none |
| 306 | MAN1B1 | mannosidase, alpha, class 1B, member 1 | No results in PubMed | none |
| 307 | ANXA4 | annexin A4 | 3 [77-79] | An increased expression of annexin IV during the implantation window plays an important role in the morphological differentiation of the uterus to the receptive state [77]. |
|  |  |  |  | There were no significant differences in plasma annexin IV levels between women with and without antiphospholipid antibodies [78]. |
|  |  |  |  | These results suggest that Anx IV enters the maternal bloodstream just after delivery and might play a role in preventing disseminated intravascular coagulopathy, and that Anx V helps to prevent clotting in the placenta during pregnancy [79]. |
| 308 | FHL3 | four and a half LIM domains 3 | No results in PubMed | none |
| 309 | PVRL3 | poliovirus receptor-related 3 | No results in PubMed | none |
| 310 | BBOX1 | butyrobetaine (gamma), 2-oxoglutarate dioxygenase (gamma-butyrobetaine hydroxylase) 1 | No results in PubMed | none |
| 311 | ZNF606 | zinc finger protein 606 | No results in PubMed | none |
| 312 | C6orf10 | chromosome 6 open reading frame 10 | No results in PubMed | none |
| 313 | UBE2D4 | ubiquitin-conjugating enzyme E2D 4 (putative) | No results in PubMed | none |
| 314 | LRRC20 | leucine rich repeat containing 20 | No results in PubMed | none |
| 315 | C21orf2 | chromosome 21 open reading frame 2 | No results in PubMed | none |
| 316 | MAGI3 | membrane associated guanylate kinase, WW and PDZ domain containing 3 | No results in PubMed | none |
| 317 | LOC100653194 | signal-regulatory protein beta-1 isoform 3-like | No results in PubMed | none |
| 318 | SRGAP3 | SLIT-ROBO Rho GTPase activating protein 3 | No results in PubMed | none |
| 319 | SIRPB1 | signal-regulatory protein beta 1 | No results in PubMed | none |
| 320 | MS4A1 | membrane-spanning 4-domains, subfamily A, member 1 (CD20) | 110 [selection 80] | B-lymphocyte antigen CD20 is an activated-glycosylated [phosphoprotein](http://en.wikipedia.org/wiki/Phosphoprotein) expressed on the surface of all [B-cells](http://en.wikipedia.org/wiki/B-cells) beginning at the pro-B phase ([CD45](http://en.wikipedia.org/wiki/CD45)R+, [CD117](http://en.wikipedia.org/wiki/CD117)+) and progressively increasing in concentration until maturity [80]. |
| 321 | C16orf57 | chromosome 16 open reading frame 57 | No results in PubMed | none |
| 322 | PRDM15 | PR domain containing 15 | No results in PubMed | none |
| 323 | ADAMDEC1 | ADAM-like, decysin 1 | No results in PubMed | none |
| 324 | FZD4 | frizzled family receptor 4 | No results in PubMed | none |
| 325 | PRSS12 | protease, serine, 12 (neurotrypsin, motopsin) | No results in PubMed | none |
| 326 | ADAMTS14 | ADAM metallopeptidase with thrombospondin type 1 motif, 14 | 2 [81-82] | A disintegrin and metalloproteinase with thrombospondin motifs (ADAMTS) is a novel family of secreted metalloproteinases. The ADAMTS-1, -4, -5, and -14 subtypes are known to be expressed in human placenta [81]. |
|  |  |  |  | The neural tube defect cells showed, compared with both healthy amniotic fluid cells and fetal fibroblasts, much lower mRNA expression levels of ADAMTS14 gene [82]. |
| 327 | PRKAR2B | protein kinase, cAMP-dependent, regulatory, type II, beta | No results in PubMed | none |
| 328 | MCM3 | minichromosome maintenance complex component 3 | No results in PubMed | none |
| 329 | RWDD2A | RWD domain containing 2A | No results in PubMed | none |
| 330 | VPS26A | vacuolar protein sorting 26 homolog A (S. pombe) | No results in PubMed | none |
| 331 | GRB2 | growth factor receptor-bound protein 2 | 1 [83] | Growth factor receptor-protein bound 2 (GRB2) upregulation in the placenta in preeclampsia implies a possible role for ras signalling [83]. |
| 332 | CENPB | centromere protein B, 80kDa | No results in PubMed | none |
| 333 | KITLG | KIT ligand | No results in PubMed | none |
| 334 | ZNF396 | zinc finger protein 396 | No results in PubMed | none |
| 335 | LILRB5 | leukocyte immunoglobulin-like receptor, subfamily B (with TM and ITIM domains), member 5 | No results in PubMed | none |
| 336 | DAK | dihydroxyacetone kinase 2 homolog (S. cerevisiae) | No results in PubMed | none |
| 337 | SPCS3 | signal peptidase complex subunit 3 homolog (S. cerevisiae) | No results in PubMed | none |
| 338 | MYO1B | myosin IB | No results in PubMed | none |
| 339 | ANO6 | anoctamin 6 | No results in PubMed | none |
| 340 | SPINK8 | serine peptidase inhibitor, Kazal type 8 (putative) | No results in PubMed | none |
| 341 | TMPRSS11BNL | TMPRSS11B N terminal-like | No results in PubMed | none |
| 342 | TMEM65 | transmembrane protein 65 | No results in PubMed | none |
| 343 | KCNJ14 | potassium inwardly-rectifying channel, subfamily J, member 14 | No results in PubMed | none |
| 344 | TGIF2 | TGFB-induced factor homeobox 2 | No results in PubMed | none |
| 345 | DNAJC25 | DnaJ (Hsp40) homolog, subfamily C , member 25 | No results in PubMed | none |
| 346 | ZNF335 | zinc finger protein 335 | No results in PubMed | none |
| 347 | SLC6A2 | solute carrier family 6 (neurotransmitter transporter, noradrenalin), member 2; (NET) | 14 [selection 84-86] | Quantitative analysis showed a significant lower expression of NET and EMT mRNAs in preeclamptic placentae as compared to the control group [84]. |
|  |  |  |  | Uncomplicated pregnancies had a higher level of placental norepinephrine transporter mRNA than complicated pregnancies. An inverse relationship between umbilical cord norepinephrine level and transporter expression was demonstrated [85]. |
|  |  |  |  | Highly significant associations were observed between tag SNPs within SLC6A2 and Attention Deficit with Hyperactivity Disorder (ADHD) [86]. |
| 348 | MAP3K13 | mitogen-activated protein kinase kinase kinase 13 | No results in PubMed | none |
| 349 | KLHL15 | kelch-like 15 (Drosophila) | No results in PubMed | none |

**References**

1. Arkwright PD, Rademacher TW, Dwek RA, Redman CW (1993) Pre-eclampsia is associated with an increase in trophoblast glycogen content and glycogen synthase activity, similar to that found in hydatidiform moles. J Clin Invest 91: 2744-53.
2. Maeyama M, Matsuo I, Nakahara K (1977) Glycogen metabolism in vesicles of hydatidiform mole in vitro. Fertil Steril 28: 851-5.
3. Kuznetsova LA, Chistiakova OV (2009) The regulation of glucose-6-phosphate dehydrogenase and glycogen synthase activities by insulin superfamily peptides in myometrium of pregnant women and its impairments under different types of diabetes mellitus. Biomed Khim 55: 663-72.
4. Strehle EM, Yu L, Rosenfeld JA, Donkervoort S, Zhou Y, et al. (2012) Genotype-phenotype analysis of 4q deletion syndrome: proposal of a critical region. Am J Med Genet A 158A: 2139-51.
5. Lee H, Jaffe AE, Feinberg JI, Tryggvadottir R, Brown S, et al. (2012) DNA methylation shows genome-wide association of NFIX, RAPGEF2 and MSRB3 with gestational age at birth. Int J Epidemiol 41: 188-99.
6. Ietta F, Wu Y, Winter J, Xu J, Wang J, et al. (2006) Dynamic HIF1A regulation during human placental development. Biol Reprod 75: 112-21.
7. Ma GC, Liu CS, Chang SP, Yeh KT, Ke YY, et al. (2008) A recurrent ITGA9 missense mutation in human fetuses with severe chylothorax: possible correlation with poor response to fetal therapy. Prenat Diagn 28: 1057-63.
8. De Toffol S, Bellone E, Dulcetti F, Ruggeri AM, Maggio PP, et al. (2010) Quantitative fluorescence-polymerase chain reaction assay for the detection of the duplication of the Charcot Marie Tooth disease type 1A critical region. Genet Test Mol Biomarkers 14: 225-31.
9. Navon R, Timmerman V, Löfgren A, Liang P, Nelis E, et al. (1995) Prenatal diagnosis of Charcot-Marie-Tooth disease type 1A (CMT1A) using molecular genetic techniques. Prenat Diagn 15: 633-40.
10. Caruccio L, Bettinotti M, Matsuo K, Sharon V, Stroncek D (2003) Expression of human neutrophil antigen-2a (NB1) is increased in pregnancy. Transfusion 43: 357-63.
11. Jarvenpaa J, Vuoristo JT, Savolainen ER, Ukkola O, Vaskivuo T, Ryynanen M (2007) Altered expression of angiogenesis-related placental genes in pre-eclampsia associated with intrauterine growth restriction. Gynecol Endocrinol 23: 351-5.
12. Heinig J, Wilhelm S, Müller H, Briese V, Bittorf T, Brock J (2000) Determination of cytokine mRNA-expression in term human placenta of patients with gestational hypertension, intrauterine growth retardation and gestational diabetes mellitus using polymerase chain reaction. Zentralbl Gynakol 122: 413-8.
13. Gurski MR, Gonzalez E, Brown EG (1999) Immunochemical localization of platelet-derived growth factor in placenta and its possible role in pre-eclampsia. J Investig Med 47: 128-33.
14. Teglund S, Olsen A, Khan WN, Frangsmyr L, Hammarstrom S (1994) The pregnancy-specific glycoprotein (PSG) gene cluster on human chromosome 19: fine structure of the 11 PSG genes and identification of 6 new genes forming a third subgroup within the carcinoembryonic antigen (CEA) family. Genomics 23: 669-684.
15. [Pihl K](http://www.ncbi.nlm.nih.gov/pubmed?term=Pihl%20K%5BAuthor%5D&cauthor=true&cauthor_uid=19911417), [Larsen T](http://www.ncbi.nlm.nih.gov/pubmed?term=Larsen%20T%5BAuthor%5D&cauthor=true&cauthor_uid=19911417), [Laursen I](http://www.ncbi.nlm.nih.gov/pubmed?term=Laursen%20I%5BAuthor%5D&cauthor=true&cauthor_uid=19911417), [Krebs L](http://www.ncbi.nlm.nih.gov/pubmed?term=Krebs%20L%5BAuthor%5D&cauthor=true&cauthor_uid=19911417), [Christiansen M](http://www.ncbi.nlm.nih.gov/pubmed?term=Christiansen%20M%5BAuthor%5D&cauthor=true&cauthor_uid=19911417) (2009) First trimester maternal serum pregnancy-specific beta-1-glycoprotein (SP1) as a marker of adverse pregnancy outcome. [Prenat Diagn](http://www.ncbi.nlm.nih.gov/pubmed/19911417) 29: 1256-61.
16. Okazaki S, Sekizawa A, Purwosunu Y, Farina A, Wibowo N, Okai T (2007) Placenta-derived, cellular messenger RNA expression in the maternal blood of preeclamptic women. Obstet Gynecol 110: 1130-6.
17. Barnett TR, Pickle W 2nd, Elting JJ (1990) Characterization of two new members of the pregnancy-specific beta 1-glycoprotein family from themyeloid cell line KG-1 and suggestion of two distinct classes of transcription unit. Biochemistry 29: 10213-8.
18. Zhao L, Triche EW, Walsh KM, Bracken MB, Saftlas AF, et al. (2012) Genome-wide association study identifies a maternal copy-number deletion in PSG11 enriched among preeclampsia patients. BMC Pregnancy Childbirth 12: 61.
19. McWilliams L, Su KY, Liang X, Liao D, Floyd S, et al. (2013) The human fetal lymphocyte lineage: identification by CD27 and LIN28B expression in B cell progenitors. J Leukoc Biol 94: 991-1001.
20. Barbaux S, Gascoin-Lachambre G, Buffat C, Monnier P, Mondon F, et al. (2012) A genome-wide approach reveals novel imprinted genes expressed in the human placenta. Epigenetics 7: 1079-90.
21. Henderson YC, Frederick MJ, Wang MT, Hollier LM, Clayman GL (2008) LBP-1b, LBP-9, and LBP-32/MGR detected in syncytiotrophoblasts from first-trimester human placental tissue and their transcriptional regulation. DNA Cell Biol 27: 71-9.
22. Katsanis N, Fisher EM (1996) The gene encoding the p60 subunit of chromatin assembly factor I (CAF1P60) maps to human chromosome 21q22.2, a region associated with some of the major features of Down syndrome. Hum Genet 98: 497-9.
23. Santner-Nanan B, Peek MJ, Khanam R, Richarts L, Zhu E, et al. (2009) Systemic increase in the ratio between Foxp3+ and IL-17-producing CD4+ T cells in healthy pregnancy but not in preeclampsia. J Immunol 183: 7023-30.
24. Anderssohn M, Maass LM, Diemert A, Lüneburg N, Atzler D, et al. (2012) Severely decreased activity of placental dimethylarginine dimethylaminohydrolase in pre-eclampsia. Eur J Obstet Gynecol Reprod Biol 161: 152-6.
25. Akbar F, Heinonen S, Pirskanen M, Uimari P, Tuomainen TP, Salonen JT (2005) Haplotypic association of DDAH1 with susceptibility to pre-eclampsia. Mol Hum Reprod 11: 73-7.
26. Best LG, Nadeau M, Bercier S, Dauphinais S, Davis J, et al. (2012) Genetic variants, endothelial function, and risk of preeclampsia among American Indians. Hypertens Pregnancy 31: 1-10.
27. Vaziri ND, Toohey J, Powers D, Keegan K, Gupta A, et al. (1986) Activation of intrinsic coagulation pathway in pre-eclampsia. Am J Med 80: 103-7.
28. Blumenstein M, Prakash R, Cooper GJ, North RA; SCOPE Consortium (2009) Aberrant processing of plasma vitronectin and high-molecular-weight kininogen precedes the onset of preeclampsia. Reprod Sci 16: 1144-52.
29. Schönfelder G, Fuhr N, Hadzidiakos D, John M, Hopp H, Paul M (2004) Preeclampsia is associated with loss of neuronal nitric oxide synthase expression in vascular smooth muscle cells of the human umbilical cord. Histopathology 44: 116-28.
30. Törnblom SA, Maul H, Klimaviciute A, Garfield RE, Byström B, et al. (2005) mRNA expression and localization of bNOS, eNOS and iNOS in human cervix at preterm and term labour. Reprod Biol Endocrinol 3: 33.
31. Nakamura H, Jasper MJ, Hull ML, Aplin JD, Robertson SA (2012) Macrophages regulate expression of α1,2-fucosyltransferase genes in human endometrial epithelial cells. Mol Hum Reprod 18: 204-15.
32. Frendo JL, Guibourdenche J, Pidoux G, Vidaud M, Luton D, et al. (2004) Trophoblast production of a weakly bioactive human chorionic gonadotropin in trisomy 21-affected pregnancy. J Clin Endocrinol Metab 89: 727-32.
33. Rana S, Karumanchi SA, Lindheimer MD (2014) Angiogenic factors in diagnosis, management, and research in preeclampsia. Hypertension 63: 198-202.
34. [Kusanovic JP](http://www.ncbi.nlm.nih.gov/pubmed?term=Kusanovic%20JP%5BAuthor%5D&cauthor=true&cauthor_uid=19900040) , [Romero R](http://www.ncbi.nlm.nih.gov/pubmed?term=Romero%20R%5BAuthor%5D&cauthor=true&cauthor_uid=19900040), [Chaiworapongsa T](http://www.ncbi.nlm.nih.gov/pubmed?term=Chaiworapongsa%20T%5BAuthor%5D&cauthor=true&cauthor_uid=19900040), [Erez O](http://www.ncbi.nlm.nih.gov/pubmed?term=Erez%20O%5BAuthor%5D&cauthor=true&cauthor_uid=19900040), [Mittal P](http://www.ncbi.nlm.nih.gov/pubmed?term=Mittal%20P%5BAuthor%5D&cauthor=true&cauthor_uid=19900040), et al. (2009) A prospective cohort study of the value of maternal plasma concentrations of angiogenic and anti-angiogenic factors in early pregnancy and midtrimester in the identification of patients destined to develop preeclampsia. [J Matern Fetal Neonatal Med](http://www.ncbi.nlm.nih.gov/pubmed/19900040) 22:1021-38.
35. Fairbanks LD, Duley JA, Shores AJ, Simmonds HA (1991) HPLC assay of uridine monophosphate synthase (UMPS) in chorionic villus samples (CVS) and erythrocytes (RBC). Adv Exp Med Biol 309B: 35-8.
36. Mistry HD, McCallum LA, Kurlak LO, Greenwood IA, Broughton Pipkin F, Tribe RM (2011) Novel expression and regulation of voltage-dependent potassium channels in placentas from women with preeclampsia. Hypertension 58: 497-504.
37. Anteby EY, Natanson-Yaron S, Greenfield C, Goldman-Wohl D, Haimov-Kochman R, et al. (2005) Human placental Hofbauer cells express sprouty proteins: a possible modulating mechanism of villous branching. Placenta 26: 476-83.
38. Natanson-Yaron S, Anteby EY, Greenfield C, Goldman-Wohl D, Hamani Y, et al. (2007) FGF 10 and Sprouty 2 modulate trophoblast invasion and branching morphogenesis. Mol Hum Reprod 13: 511-9.
39. Vainer OB, Katokhin AV, Kustov SM, Vlassov VV, Laktionov PP (2008) A new Y chromosome marker for noninvasive fetal gender determination. Ann N Y Acad Sci 1137: 157-61.
40. Sismani C, Donoghue J, Alexandrou A, Karkaletsi M, Christopoulou S, et al. (2013) A prenatally ascertained, maternally inherited 14.8 Mb duplication of chromosomal bands Xq13.2-q21.31 associated with multiple congenital abnormalities in a male fetus. Gene 530: 138-42.
41. Clayton PE, Hanson D, Magee L, Murray PG, Saunders E, et al. (2012) Exploring the spectrum of 3-M syndrome, a primordial short stature disorder of disrupted ubiquitination. Clin Endocrinol (Oxf) 77: 335-42.
42. Penneys NS, Muench KH (1974) Human placental tryptophanyl transfer ribonucleic acid synthetase. Purification and subunit structure. Biochemistry 13: 560-5.
43. Penneys NS, Muench KH (1974) Human tryptophanyl transfer ribonucleic acid synthetase. Comparison of the kinetic mechanism to that of the Escherichia coli tryptophanyl transfer ribonucleic acid synthetase. Biochemistry 13: 566-71.
44. Nielsen JE, Koefoed P, Kjaergaard S, Jensen LN, Nørremølle A, Hasholt L (2004) Prenatal diagnosis of autosomal dominant hereditary spastic paraplegia (SPG4) using direct mutation detection. Prenat Diagn 24: 363-6.
45. Vidal-Taboada JM, Bergoñon S, Scartezzini P, Egeo A, Nizetic D, Oliva R (1997) High-resolution physical map and identification of potentially regulatory sequences of the human SH3BGR located in the Down syndrome chromosomal region. Biochem Biophys Res Commun 241: 321-6.
46. Scartezzini P, Egeo A, Colella S, Fumagalli P, Arrigo P, et al. (1997) Cloning a new human gene from chromosome 21q22.3 encoding a glutamic acid-rich protein expressedin heart and skeletal muscle. Hum Genet 99: 387-92.
47. Quinn LM, Latham SE, Kalionis B (2000) The homeobox genes MSX2 and MOX2 are candidates for regulating epithelial-mesenchymal cell interactions in the human placenta. Placenta 21 Suppl A: S50-4.
48. Morrish DW, Dakour J, Li H (2001) Life and death in the placenta: new peptides and genes regulating human syncytiotrophoblast and extravillous cytotrophoblast lineage formation and renewal. Curr Protein Pept Sci 2: 245-59.
49. Laskarin G, Redzovic A, Vlastelic I, Haller H, Medancic SS, et al. (2011) Tumor-associated glycoprotein (TAG-72) is a natural ligand for the C-type lectin-like domain that induces anti-inflammatory orientation of early pregnancy decidual CD1a+ dendritic cells. J Reprod Immunol 88: 12-23.
50. DeLoia JA, Burlingame JM, Krasnow JS (1997) Differential expression of G1 cyclins during human placentogenesis. Placenta 18: 9-16.
51. Wathlet S, Adriaenssens T, Segers I, Verheyen G, Janssens R, et al. (2012) New candidate genes to predict pregnancy outcome in single embryo transfer cycles when using cumulus cell gene expression. Fertil Steril 98: 432-9.e1-4.
52. Allegra A, Marino A, Coffaro F, Lama A, Rizza G, et al. (2009) Is there a uniform basal endometrial gene expression profile during the implantation window in women who became pregnant in a subsequent ICSI cycle? Hum Reprod 24: 2549-57.
53. Uusküla L, Männik J, Rull K, Minajeva A, Kõks S, et al. (2012) Mid-gestational gene expression profile in placenta and link to pregnancy complications. PLoS One 7: e49248.
54. Winston N (2001) Regulation of early embryo development: functional redundancy between cyclin subtypes. Reprod Fertil Dev 13: 59-67.
55. Wu W, Wang Y, Xu Y, Liu Y, Wang Y, Zhang H (2014) Dysregulated activation of c-Src in gestational trophoblastic disease contributes to its aggressive progression. Placenta 35: 824-30.
56. Rama S, Petrusz P, Rao AJ (2004) Hormonal regulation of human trophoblast differentiation: a possible role for 17beta-estradiol and GnRH. Mol Cell Endocrinol 218: 79-94.
57. Hannan NJ, Jones RL, White CA, Salamonsen LA (2006) The chemokines, CX3CL1, CCL14, and CCL4, promote human trophoblast migration at the feto-maternal interface. Biol Reprod 74: 896-904.
58. Agachan B, Attar R, Isbilen E, Aydogan HY, Sozen S, et al. (2010) Association of monocyte chemotactic protein-1 and CC chemokine receptor 2 gene variants with preeclampsia. J Interferon Cytokine Res 30: 673-6.
59. Björkander S, Heidari-Hamedani G, Bremme K, Gunnarsson I, Holmlund U (2013) Peripheral monocyte expression of the chemokine receptors CCR2, CCR5 and CXCR3 is altered at parturition in healthy women and in women with systemic lupus erythematosus. Scand J Immunol 77: 200-12.
60. Enquobahrie DA, Williams MA, Qiu C, Muhie SY, Slentz-Kesler K, et al. (2009) Early pregnancy peripheral blood gene expression and risk of preterm delivery: a nested case control study. BMC Pregnancy Childbirth 9: 56.
61. Cudmore MJ, Ramma W, Cai M, Fujisawa T, Ahmad S, et al. (2012) Resveratrol inhibits the release of soluble fms-like tyrosine kinase (sFlt-1) from human placenta. Am J Obstet Gynecol 206: 253.e10-5.
62. Saben J, Zhong Y, Gomez-Acevedo H, Thakali KM, Borengasser SJ, et al. (2013) Early growth response protein-1 mediates lipotoxicity-associated placental inflammation: role in maternal obesity. Am J Physiol Endocrinol Metab 305: E1-14.
63. Wang X, Athayde N, Trudinger B (2003) Fetal plasma stimulates endothelial cell production of cytokines and the family of suppressor of cytokine signaling in umbilical placental vascular disease. Am J Obstet Gynecol 188: 510-6.
64. Wang X, Athayde N, Trudinger B (2003) A proinflammatory cytokine response is present in the fetal placental vasculature in placental insufficiency. Am J Obstet Gynecol 189: 1445-51.
65. Street ME, Viani I, Ziveri MA, Volta C, Smerieri A, Bernasconi S (2011) Impairment of insulin receptor signal transduction in placentas of intra-uterine growth-restricted newborns and its relationship with fetal growth. Eur J Endocrinol 164: 45-52.
66. Petroff MG, Kharatyan E, Torry DS, Holets L (2005) The immunomodulatory proteins B7-DC, B7-H2, and B7-H3 are differentially expressed across gestation in the human placenta. Am J Pathol 167: 465-73.
67. Nagamatsu T, Barrier BF, Schust DJ (2011) The regulation of T-cell cytokine production by ICOS-B7H2 interactions at the human fetomaternal interface. Immunol Cell Biol 89: 417-25.
68. Wareing M, Bai X, Seghier F, Turner CM, Greenwood SL, et al. (2006) Expression and function of potassium channels in the human placental vasculature. Am J Physiol Regul Integr Comp Physiol 291: R437-46.
69. Noorlander CW, de Graan PN, Nikkels PG, Schrama LH, Visser GH (2004) Distribution of glutamate transporters in the human placenta. Placenta 25: 489-95.
70. Khan I, al-Yatama M, Nandakumaran M (1999) Expression of the Na(+)-H+ exchanger isoform-1 and cyclooxygenases in human placentas: their implications in preeclampsia. Biochem Mol Biol Int 47: 715-22.
71. Hughes JL, Doughty IM, Glazier JD, Powell TL, Jansson T, et al. (2000) Activity and expression of the Na(+)/H(+) exchanger in the microvillous plasma membrane of the syncytiotrophoblast in relation to gestation and small for gestational age birth. Pediatr Res 48: 652-9.
72. Johansson M, Glazier JD, Sibley CP, Jansson T, Powell TL (2002) Activity and protein expression of the Na+/H+ exchanger is reduced in syncytiotrophoblast microvillous plasma membranes isolated from preterm intrauterine growth restriction pregnancies. J Clin Endocrinol Metab 87: 5686-94.
73. Thaler CJ (2014) Folate Metabolism and Human Reproduction. Geburtshilfe Frauenheilkd 74: 845-51.
74. Lyttle B, Chai J, Gonzalez JM, Xu H, Sammel M, Elovitz MA (2009) The negative regulators of the host immune response: an unexplored pathway in preterm birth. Am J Obstet Gynecol 201: 284.e1-7.
75. Pierce A, Bliesner B, Xu M, Nielsen-Preiss S, Lemke G, et al. (2008) Axl and Tyro3 modulate female reproduction by influencing gonadotropin-releasing hormone neuron survival and migration. Mol Endocrinol 22: 2481-95.
76. Chen CP, Huang MC, Chen YY, Chern SR, Wu PS, et al. (2013) Prenatal diagnosis of de novo interstitial deletions involving 5q23.1-q23.3 and 18q12.1-q12.3 by array CGH using uncultured amniocytes in a pregnancy with fetal interrupted aortic arch and atrial septal defect. Gene 531: 496-501.
77. Li J, Tan Z, Li MT, Liu YL, Liu Q, et al. (2006) Study of altered expression of annexin IV and human endometrial receptivity. Zhonghua Fu Chan Ke Za Zhi 41: 803-5.
78. Ulander VM, Stefanovic V, Masuda J, Suzuki K, Hiilesmaa V, Kaaja R (2007) Plasma levels of annexins IV and V in relation to antiphospholipid antibody status in women with a history of recurrent miscarriage. Thromb Res 120: 865-70.
79. Masuda J, Takayama E, Satoh A, Ida M, Shinohara T, et al. (2004) Levels of annexin IV and V in the plasma of pregnant and postpartum women. Thromb Haemost 91: 1129-36.
80. Hardy R (2008) Chapter 7: B Lymphocyte Development and Biology. In: Paul WE. Fundamental Immunology (Book) (6th ed.). Philadelphia: Lippincott Williams & Wilkins. pp. 237–269.
81. Lee SY, Lee HS, Gil M, Kim CJ, Lee YH, et al. (2014) Differential expression patterns of a disintegrin and metalloproteinase with thrombospondin motifs (ADAMTS) -1, -4, -5, and -14 in human placenta and gestational trophoblastic diseases. Arch Pathol Lab Med 138: 643-50.
82. Hosper NA, Bank RA, van den Berg PP (2014) Human amniotic fluid-derived mesenchymal cells from fetuses with a neural tube defect do not deposit collagen type i protein after TGF-β1 stimulation in vitro. Stem Cells Dev 23: 555-62.
83. Anteby EY, Ayesh S, Shochina M, Hamani Y, Schneider T, et al. (2005) Growth factor receptor-protein bound 2 (GRB2) upregulation in the placenta in preeclampsia implies a possible role for ras signalling. Eur J Obstet Gynecol Reprod Biol 118: 174-81.
84. Bottalico B, Larsson I, Brodszki J, Hernandez-Andrade E, Casslén B, et al. (2004) Norepinephrine transporter (NET), serotonin transporter (SERT), vesicular monoamine transporter (VMAT2) and organic cation transporters (OCT1, 2 and EMT) in human placenta from pre-eclamptic and normotensive pregnancies. Placenta 25: 518-29.
85. Bzoskie L, Yen J, Tseng YT, Blount L, Kashiwai K, Padbury JF (1997) Human placental norepinephrine transporter mRNA: expression and correlation with fetal condition at birth. Placenta 18: 205-10.
86. Thakur GA, Sengupta SM, Grizenko N, Choudhry Z, Joober R (2012) Comprehensive phenotype/genotype analyses of the norepinephrine transporter gene (SLC6A2) in ADHD: relation to maternal smoking during pregnancy. PLoS One 7: e49616.
