# Supplementary material for: First Trimester Screening of Circulating C19MC microRNAs Can Predict Subsequent Onset of Gestational Hypertension
Source: PLoS One. 2014 Dec 15;9(12):e113735. doi: 10.1371/journal.pone.0113735 (PMC4266496; doi:10.1371/journal.pone.0113735)
Supplement: S2 Table — Function of target genes of miR-517* (miR-517-5p) in relation to pregnancy. (DOC) [file pone.0113735.s002.doc]

**Table S2. Function of target genes of differentially expressed extracellular C19MC microRNAs in patients developing gestational hypertension in relation to pregnancy**

**miR-517^*^ (miR-517-5p)**

| **No.** | **GENE** | **GENE full name** | **Total number of references,** | **The role in gestation** |
| --- | --- | --- | --- | --- |
|  | **official symbol** |  | **list of references in PubMed (humans)** |  |
| 1 | [CYLD](http://www.ncbi.nlm.nih.gov/entrez/query.fcgi?db=gene&cmd=Retrieve&dopt=full_report&list_uids=1540) | cylindromatosis (turban tumor syndrome) | No results in PubMed | none |
| 2 | RND3 | Rho family GTPase 3 | 3 [1-3] | Universal Rnd family up-regulation during pregnancy may have an important role for negative-feedback control of uterine contraction during gestation by inhibiting RhoA-mediated increase in Ca(2+) sensitivity of contractile elements [1, 2]. |
|  |  |  |  | UTR-3  RND3  SNP  is associated with preeclampsia susceptibility and risk for cardiovascular disease in an extended Australian and New Zealand familial cohort [3]. |
| 3 | SLC9A3R1 | solute carrier family 9 (sodium/hydrogen exchanger), member 3 regulator 1 | No results in PubMed | none |
| 4 | CUL4B | cullin 4B | 2 [4-5] | Cullin family, including CUL4B, represent a new set of markers of IUGR [4, 5]. No significant difference could be seen between isolated PE and control placental samples but a significant increase was observed in placentas when IUGR was present [4]. |
| 5 | QRSL1 | glutaminyl-tRNA synthase (glutamine-hydrolyzing)-like 1 | No results in PubMed | none |
| 6 | RAP2C | RAP2C, member of RAS oncogene family | No results in PubMed | none |
| 7 | SPICE1 | spindle and centriole associated protein 1 | No results in PubMed | none |
| 8 | C2orf63 | chromosome 2 open reading frame 63 | No results in PubMed | none |
| 9 | TTC33 | tetratricopeptide repeat domain 33 | No results in PubMed | none |
| 10 | SFRP4 | secreted frizzled-related protein 4 | 6 [6-11] | Changes (down-regulation) in SFRP4 gene expression occur as the myometrium differentiates from non-pregnant to pregnant status [6]. Decidualization is associated with decreased expression of SFRP4 gene [7]. |
|  |  |  |  | SFRP4 has a role in placental development and implantation, and may be an important factor in the development of the decidual fibrinoid zone, and in trophoblast apoptosis and a band of apoptosis in the underlying decidua deep into the trophoblast [8]. |
|  |  |  |  | In euploid fetuses, underexpressed SFRP4 gene is possibly involved in mechanisms associated with the abnormal NT thickness [9]. |
|  |  |  |  | Increased placental expression of SFRP4 may be associated with the pathogenesis of severe PE [10]. |
|  |  |  |  | SFRP4 gene is among the set of genes identified to be differentially expressed in polycystic ovary syndrome patient-derived cumulus cells at different stages (MII or MI) of oocyte nuclear maturation [11]. |
| 11 | LCP1 | lymphocyte cytosolic protein 1 (L-plastin) | No results in PubMed | none |
| 12 | FAM103A1 | family with sequence similarity 103, member A1 | No results in PubMed | none |
| 13 | PLEKHA3 | pleckstrin homology domain containing, family A (phosphoinositide binding specific) member 3 | No results in PubMed | none |
| 14 | FAM199X | family with sequence similarity 199, X-linked | No results in PubMed | none |
| 15 | CDC20B | cell division cycle 20 homolog B (S. cerevisiae) | No results in PubMed | none |
| 16 | MCM4 | minichromosome maintenance complex component 4 | No results in PubMed | none |
| 17 | MITF | microphthalmia-associated transcription factor | No results in PubMed | none |
| 18 | SSPN | sarcospan (Kras oncogene-associated gene) | No results in PubMed | none |
| 19 | RNF14 | ring finger protein 14 | No results in PubMed | none |
| 20 | CDH26 | cadherin 26 | No results in PubMed | none |
| 21 | SDHC | succinate dehydrogenase complex, subunit C, integral membrane protein, 15kDa | 1 [12] | SDHC mutations cause increased O2(.-) production, metabolic oxidative stress, and genomic instability and that mutations in genes coding for mitochondrial electron transport chain proteins can contribute to phenotypic changes associated with cancer cells [12]. |
| 22 | ZNF454 | zinc finger protein 454 | No results in PubMed | none |
| 23 | ACPP | acid phosphatase, prostate | No results in PubMed | none |
| 24 | MAPK9 | mitogen-activated protein kinase 9 | No results in PubMed | none |
| 25 | SLC25A33 | solute carrier family 25, member 33 | No results in PubMed | none |
| 26 | CBX8 | chromobox homolog 8 | No results in PubMed | none |
| 27 | RABEP1 | rabaptin, RAB GTPase binding effector protein 1 | No results in PubMed | none |
| 28 | ATAT1 | alpha tubulin acetyltransferase 1 | No results in PubMed | none |
| 29 | FAM122A | family with sequence similarity 122A | No results in PubMed | none |
| 30 | TNPO2 | transportin 2 | No results in PubMed | none |
| 31 | OR51E1 | olfactory receptor, family 51, subfamily E, member 1 | No results in PubMed | none |
| 32 | GEN1 | Gen homolog 1, endonuclease (Drosophila) | No results in PubMed | none |
| 33 | CALN1 | calneuron 1 | No results in PubMed | none |
| 34 | DCBLD1 | discoidin, CUB and LCCL domain containing 1 | No results in PubMed | none |
| 35 | WDR33 | WD repeat domain 33 | No results in PubMed | none |
| 36 | MAGED1 | melanoma antigen family D, 1 | No results in PubMed | none |
| 37 | KCTD5 | potassium channel tetramerisation domain containing 5 | No results in PubMed | none |
| 38 | PIK3R4 | phosphoinositide-3-kinase, regulatory subunit 4 | No results in PubMed | none |
| 39 | SLC7A11 | solute carrier family 7 (anionic amino acid transporter light chain, xc- system), member 11 | No results in PubMed | none |
| 40 | LARP7 | La ribonucleoprotein domain family, member 7 | No results in PubMed | none |
| 41 | MAGEF1 | melanoma antigen family F, 1 | No results in PubMed | none |
| 42 | TOR1AIP2 | torsin A interacting protein 2 | No results in PubMed | none |
| 43 | SLC39A10 | solute carrier family 39 (zinc transporter), member 10 | No results in PubMed | none |
| 44 | BHLHE22 | basic helix-loop-helix family, member e22 | No results in PubMed | none |
| 45 | UFC1 | ubiquitin-fold modifier conjugating enzyme 1 | No results in PubMed | none |
| 46 | SFXN1 | sideroflexin 1 | No results in PubMed | none |
| 47 | DNAJC27 | DnaJ (Hsp40) homolog, subfamily C, member 27 | No results in PubMed | none |
| 48 | PLAA | phospholipase A2-activating protein | No results in PubMed | none |
| 49 | PKD2L2 | polycystic kidney disease 2-like 2 | No results in PubMed | none |
| 50 | HLTF | helicase-like transcription factor | No results in PubMed | none |
| 51 | MTDH | metadherin | 2 [13,14] | No pregnancy-related studies are available, but involvement in HIF-1 alpha mediated angiogenesis and RNA-induced silencing complex and miRNA functions [13]. |
|  |  |  |  | MTDH could be a key factor inducing inflammatory responses for progression of various diseases. A recent study indicating that AEG-1/MTDH/LYRIC can induce protective autophagy supports a potential role in promoting cell survival [14]. |
| 52 | DIP2C | DIP2 disco-interacting protein 2 homolog C (Drosophila) | No results in PubMed | none |
| 53 | PHIP | pleckstrin homology domain interacting protein | No results in PubMed | none |
| 54 | FUT1 | fucosyltransferase 1 (galactoside 2-alpha-L-fucosyltransferase, H blood group) | 1 [15] | **Macrophage-derived factors including LIF facilitate development of an implantation-receptive endometrium by regulating surface glycan structures in epithelial cells.** **Expression of FUT1 mRNA was associated with increased fucosylation of cell surface glycoproteins** in**human endometrial epithelial cells** [15]. |
| 55 | USP46 | ubiquitin specific peptidase 46 | No results in PubMed | none |
| 56 | CDC7 | cell division cycle 7 homolog (S. cerevisiae) | No results in PubMed | none |
| 57 | TBCCD1 | TBCC domain containing 1 | No results in PubMed | none |
| 58 | CUL3 | cullin 3 | No results in PubMed | none |
| 59 | TTC37 | tetratricopeptide repeat domain 37 | No results in PubMed | none |
| 60 | **LHCGR** | **luteinizing hormone/choriogonadotropin receptor** | **20 [selection 16-18]** | This gene encodes the receptor for both luteinizing hormone and choriogonadotropin. |
|  |  |  |  | High human chorionic gonadotropin concentrations in the first trimester were associated with reduced risk for preterm preeclampsia compared with low human chorionic gonadotropin [16, 17]. |
|  |  |  |  | **Serum soluble LHCGR and hCG-sLHCGR complex have significant potential as first trimester screening markers for predicting pathological outcomes in pregnancy (stillbirth, Down's syndrome, preterm delivery and preeclampsia) [18].** |
| 61 | TMPO | thymopoietin | No results in PubMed | none |
| 62 | ALDH9A1 | aldehyde dehydrogenase 9 family, member A1 | No results in PubMed | none |
| 63 | SPINLW1-WFDC6 | SPINLW1-WFDC6 readthrough | No results in PubMed | none |
| 64 | GCNT1 | glucosaminyl (N-acetyl) transferase 1, core 2 | No results in PubMed | none |
| 65 | ZNF136 | zinc finger protein 136 | No results in PubMed | none |
| 66 | OAS2 | 2'-5'-oligoadenylate synthetase 2, 69/71kDa | No results in PubMed | none |
| 67 | VPS13B | vacuolar protein sorting 13 homolog B (yeast) | No results in PubMed | none |
| 68 | NPFFR2 | neuropeptide FF receptor 2 | No results in PubMed | none |
| 69 | BLVRB | biliverdin reductase B (flavin reductase (NADPH)) | No results in PubMed | none |
| 70 | BAG2 | BCL2-associated athanogene 2 | No results in PubMed | none |
| 71 | IL6ST | interleukin 6 signal transducer (gp130, oncostatin M receptor) | No results in PubMed | none |
| 72 | MTMR2 | myotubularin related protein 2 | No results in PubMed | none |
| 73 | ZNF124 | zinc finger protein 124 | No results in PubMed | none |
| 74 | EIF2AK3 | eukaryotic translation initiation factor 2-alpha kinase 3 | No results in PubMed | none |
| 75 | GPR161 | G protein-coupled receptor 161 | No results in PubMed | none |
| 76 | LOC100294341 | ADP-ribosylation factor-like protein 17-like | No results in PubMed | none |
| 77 | SDC4 | syndecan 4 | 7 [19-25] | Syndecans are important players in the placenta for the establishment of the fetal-maternal inter-communication [19]. |
|  |  |  |  | Syndecan-4 is expressed in the fetal vessels in the placental labyrinth and syndecan-4 deficiency impairs the fetal vessels in the placenta [20]. |
|  |  |  |  | Altered expression patterns of syndecans were observed in gestational trophoblastic disease such as hydatidiform mole, invasive mole, and choriocarcinoma [21]. |
|  |  |  |  | Differential expression of syndecan 4 was detected in idiopathic FGR placentas compared with controls. Reduced levels of syndecan expression may result in increased placental thrombosis in the uteroplacental circulation and may therefore contribute to the pathogenesis of FGR [22]. |
|  |  |  |  | Cumulus cell SDC4 gene expression predicts better cleavage-stage embryo or blastocyst development and**pregnancy** for ICSI patients [23]. |
|  |  |  |  | Monolayer of immortalized human trophoblast cells derived from term placenta (TCL-1 cells) do not express syndecan 4 [24]. |
|  |  |  |  | Syndecan-4 is a potential receptor for CXCL12 in human cytotrophoblasts [25]. |
| 78 | PRR18 | proline rich 18 | No results in PubMed | none |
| 79 | ARL17A | ADP-ribosylation factor-like 17A | No results in PubMed | none |
| 80 | ACO2 | aconitase 2, mitochondrial | No results in PubMed | none |
| 81 | MORC1 | MORC family CW-type zinc finger 1 | No results in PubMed | none |
| 82 | TRPA1 | transient receptor potential cation channel, subfamily A, member 1 | No results in PubMed | none |
| 83 | FOXK1 | forkhead box K1 | No results in PubMed | none |
| 84 | CKAP2 | cytoskeleton associated protein 2 | No results in PubMed | none |
| 85 | GTF3C4 | general transcription factor IIIC, polypeptide 4, 90kDa | No results in PubMed | none |
| 86 | RAD23B | RAD23 homolog B (S. cerevisiae) | No results in PubMed | none |
| 87 | MAPKAPK5 | mitogen-activated protein kinase-activated protein kinase 5 | No results in PubMed | none |
| 88 | ITM2B | integral membrane protein 2B | No results in PubMed | none |
| 89 | USP31 | ubiquitin specific peptidase 31 | No results in PubMed | none |
| 90 | YPEL1 | yippee-like 1 (Drosophila) | No results in PubMed | none |
| 91 | KIAA1530 | KIAA1530 | No results in PubMed | none |
| 92 | FUT9 | fucosyltransferase 9 (alpha (1,3) fucosyltransferase) | 1 [26] | FUT9 codes for a fucosyl-transferase that is catalyzing the last step in the biosynthesis of the Lewis-x antigen, which forms part of the Lewis blood group-related antigens. These results therefore suggest an involvement of this antigen in the pathogenesis of placental malaria infection [26]. |
| 93 | RCOR3 | REST corepressor 3 | No results in PubMed | none |
| 94 | FAM177A1 | family with sequence similarity 177, member A1 | No results in PubMed | none |
| 95 | SENP8 | SUMO/sentrin specific peptidase family member 8 | No results in PubMed | none |
| 96 | NCBP1 | nuclear cap binding protein subunit 1, 80kDa | No results in PubMed | none |
| 97 | ATG14 | ATG14 autophagy related 14 homolog (S. cerevisiae) | No results in PubMed | none |
| 98 | CUL4A | cullin 4A | No results in PubMed | none |
| 99 | MBD2 | methyl-CpG binding domain protein 2 | 2 [27, 28] | MBD2 is upregulated during the prenatal development of the human mammary gland, deregulation of MBD2 occurs in human breast cancers [27]. |
|  |  |  |  | In both male and female fetal gonads expression of MBD2 gene which may be implicated in chromatin remodelling of methylated genomic DNA sequences, is tightly linked to DNMT expression. The sex-specific time windows for concomitant upregulation of MBD2 are associated with prenatal remethylation of the human male and female germ line [28]. |
| 100 | ZCCHC10 | zinc finger, CCHC domain containing 10 | 1 [29] | Pre-pregnancy maternal BMI might lead to alterations in offspring DNA methylation in genes relevant to the development of a range of complex chronic diseases, providing evidence of trans-generational influence on disease susceptibility via epigenetic mechanism [29]. |
| 101 | PRMT2 | protein arginine methyltransferase 2 | No results in PubMed | none |
| 102 | MKL2 | MKL/myocardin-like 2 | No results in PubMed | none |
| 103 | ZNF620 | zinc finger protein 620 | No results in PubMed | none |
| 104 | IQSEC1 | IQ motif and Sec7 domain 1 | No results in PubMed | none |
| 105 | CLMP | CXADR-like membrane protein | No results in PubMed | none |
| 106 | FEZ2 | fasciculation and elongation protein zeta 2 (zygin II) | No results in PubMed | none |
| 107 | DDX46 | DEAD (Asp-Glu-Ala-Asp) box polypeptide 46 | No results in PubMed | none |
| 108 | TIGD3 | tigger transposable element derived 3 | No results in PubMed | none |
| 109 | CCNG1 | cyclin G1 | 1 [30] | G1 cyclins D3 and E are important cell cycle regulatory proteins, and further, that G1 cyclin E may function in trophoblast terminal differentiation as well [30]. |
| 110 | MCM9 | minichromosome maintenance complex component 9 | No results in PubMed | none |
| 111 | DGKE | diacylglycerol kinase, epsilon 64kDa | 1 [31] | Most thrombotic microangiopathies are thus characterized by misdirected complement activation affecting endothelial cell and platelet integrity. The attacks can be triggered by infections, pregnancy, drugs or trauma [31]. |
| 112 | HMGN3 | high mobility group nucleosomal binding domain 3 | No results in PubMed | none |
| 113 | ADARB2 | adenosine deaminase, RNA-specific, B2 | No results in PubMed | none |
| 114 | LOC100652926 | putative uncharacterized protein FLJ46541-like | No results in PubMed | none |
| 115 | AEBP2 | AE binding protein 2 | No results in PubMed | none |
| 116 | ZFP62 | zinc finger protein 62 homolog (mouse) | No results in PubMed | none |
| 117 | CTAGE1 | cutaneous T-cell lymphoma-associated antigen 1 | No results in PubMed | none |
| 118 | PLEKHA2 | pleckstrin homology domain containing, family A (phosphoinositide binding specific) member 2 | No results in PubMed | none |
| 119 | LMNB2 | lamin B2 | 1 [32] | **No mutation was found in LMNB2 gene known to be associated with congenital lipodystrophy in a patient with** Marfan syndrome with neonatal progeroid syndrome-like lipodystrophy **[32].** |
| 120 | SNAP25 | synaptosomal-associated protein, 25kDa | No results in PubMed | none |
| 121 | GIMAP4 | GTPase, IMAP family member 4 | No results in PubMed | none |
| 122 | ZNF527 | zinc finger protein 527 | No results in PubMed | none |
| 123 | FAS | Fas (TNF receptor superfamily, member 6) | 171 [selection 33-37] | Play a central role in regulation of programmed cell death. There are evidences about the increased apoptosis in extravillous trophoblasts of placentas from PE pregnancies and intrauterine growth restriction (IUGR) [33, 34]. |
|  |  |  |  | The FAS and FASL polymorphisms have been reported to be associated with increased risk of PE [35-37]. |
| 124 | DNA2 | DNA replication helicase 2 homolog (yeast) | No results in PubMed | none |
| 125 | PTPN9 | protein tyrosine phosphatase, non-receptor type 9 | No results in PubMed | none |
| 126 | SNX27 | sorting nexin family member 27 | No results in PubMed | none |
| 127 | SH3BGRL2 | SH3 domain binding glutamic acid-rich protein like 2 | 1 [38] | The meta-analysis of PBMC transcription profiles characterized each type of diabetes revealing that gestational and type 1 diabetes were transcriptionally related [38]. 19 known genes were shared by type 1, type 2 and gestational diabetes, highlighting EGF, FAM46C, HBEGF, ID1, SH3BGRL2, VEPH1, and TMEM158 genes [38]. |
| 128 | PARP11 | poly (ADP-ribose) polymerase family, member 11 | No results in PubMed | none |
| 129 | IRAK3 | interleukin-1 receptor-associated kinase 3 | No results in PubMed | none |
| 130 | DLGAP4 | discs, large (Drosophila) homolog-associated protein 4 | No results in PubMed | none |
| 131 | SEPSECS | Sep (O-phosphoserine) tRNA:Sec (selenocysteine) tRNA synthase | No results in PubMed | none |
| 132 | OTUD4 | OTU domain containing 4 | No results in PubMed | none |
| 133 | FKBP9 | FK506 binding protein 9, 63 kDa | No results in PubMed | none |
| 134 | ZBTB8A | zinc finger and BTB domain containing 8A | No results in PubMed | none |
| 135 | PPP1R14C | protein phosphatase 1, regulatory (inhibitor) subunit 14C | No results in PubMed | none |
| 136 | ZNF549 | zinc finger protein 549 | No results in PubMed | none |
| 137 | RPRD2 | regulation of nuclear pre-mRNA domain containing 2 | No results in PubMed | none |
| 138 | ZNF716 | zinc finger protein 716 | No results in PubMed | none |
| 139 | GOLPH3 | golgi phosphoprotein 3 (coat-protein) | No results in PubMed | none |
| 140 | PTPN14 | protein tyrosine phosphatase, non-receptor type 14 | No results in PubMed | none |
| 141 | C19orf12 | chromosome 19 open reading frame 12 | No results in PubMed | none |
| 142 | ARHGAP11A | Rho GTPase activating protein 11A | No results in PubMed | none |
| 143 | CASP10 | caspase 10, apoptosis-related cysteine peptidase | 3 [39-41] | Caspase-10 is involved in apoptosis in the preeclamptic placenta. The expression of caspase-10 was significantly increased in full-term preeclamptic placentas [39]. |
|  |  |  |  | The downregulation of caspase 10 might contribute to the pathogenesis of choriocarcinoma [40]. |
|  |  |  |  | The comparative analysis showed a significant different expression in CASP10 gene between patients who achieved a successful pregnancy spontaneously or after subsequent intracytoplasmic sperm injection cycles and patients who did not achieve a pregnancy after at least two failed ICSI cycles [41]. |
| 144 | TERF2IP | telomeric repeat binding factor 2, interacting protein | No results in PubMed | none |
| 145 | MYSM1 | Myb-like, SWIRM and MPN domains 1 | No results in PubMed | none |
| 146 | FAM200B | family with sequence similarity 200, member B | No results in PubMed | none |
| 147 | NFIB | nuclear factor I/B | 2 [42, 43] | NFIB gene is proposed as a non-invasive biomarker for embryo potential and a prognostic indicator of successful pregnancy. New potential strategy for competent embryo selection is proposed [42, 43]. |
| 148 | UBE2QL1 | ubiquitin-conjugating enzyme E2Q family-like 1 | No results in PubMed | none |
| 149 | CRYM | crystallin, mu | No results in PubMed | none |
| 150 | HIP1 | huntingtin interacting protein 1 | No results in PubMed | none |
| 151 | FAM60A | family with sequence similarity 60, member A | No results in PubMed | none |
| 152 | TWIST1 | twist homolog 1 (Drosophila) | 6 [44-49] | Essential role for TWIST1 in the initiation of human decidualization was described [44]. |
|  |  |  |  | Upregulation of TWIST1 in human myometrium at labor was observed [45]. |
|  |  |  |  | Twist is involved in placental maturation; the expression of twist was disturbed in those placentas with chromosomal aberrations [46]. |
|  |  |  |  | Mutations in TWIST have been identified in certain syndromic craniosynostosis [47, 48]. |
|  |  |  |  | Twist is an upstream regulator of N-cadherin-mediated invasion of human trophoblastic cells [49]. |
| 153 | PID1 | phosphotyrosine interaction domain containing 1 | No results in PubMed | none |
| 154 | OSBPL8 | oxysterol binding protein-like 8 | No results in PubMed | none |
| 155 | MUDENG | MU-2/AP1M2 domain containing, death-inducing | No results in PubMed | none |
| 156 | HHLA1 | HERV-H LTR-associating 1 | No results in PubMed | none |
| 157 | GCLC | glutamate-cysteine ligase, catalytic subunit | 1 [50] | Maternal-fetal genotype interactions between haplotypes in three of seven genes, GCLC, GSTM3, and RFC1, are associated with nonsyndromicconotruncal heart defects [50]. |
| 158 | SLC16A1 | solute carrier family 16, member 1 (monocarboxylic acid transporter 1) | No results in PubMed | none |
| 159 | KIAA1486 | KIAA1486 | No results in PubMed | none |
| 160 | BMPER | BMP binding endothelial regulator | No results in PubMed | none |
| 161 | C14orf166 | chromosome 14 open reading frame 166 | No results in PubMed | none |
| 162 | ZFP30 | zinc finger protein 30 homolog (mouse) | No results in PubMed | none |
| 163 | PLK2 | polo-like kinase 2 |  |  |
| 164 | F9 | coagulation factor IX | No results in PubMed | none |
| 165 | ZNF304 | zinc finger protein 304 | No results in PubMed | none |
| 166 | GPC6 | glypican 6 | No results in PubMed | none |
| 167 | C16orf42 | chromosome 16 open reading frame 42 | No results in PubMed | none |
| 168 | LRRC55 | leucine rich repeat containing 55 | No results in PubMed | none |
| 169 | IMPACT | Impact homolog (mouse) | No results in PubMed | none |
| 170 | ARSK | arylsulfatase family, member K | No results in PubMed | none |
| 171 | SNTB1 | syntrophin, beta 1 (dystrophin-associated protein A1, 59kDa, basic component 1) | No results in PubMed | none |
| 172 | LCE6A | late cornified envelope 6A | No results in PubMed | none |
| **173** | **PAPPA** | **pregnancy-associated plasma protein A, pappalysin 1** | **1712 [selection 51, 52]** | This gene encodes a secreted metalloproteinase which cleaves insulin-like growth factor binding proteins (IGFBPs). It is thought to be involved in local proliferative processes such as wound healing and bone remodeling. **Low plasma levels of this protein have been suggested as a biochemical marker for pregnancies with aneuploid fetuses. PAPP-A is currently also a part of combination screening to predict preeclampsia. Nevertheless, PAPP-A as an individual serum marker has not high enough detection rate to be utilized clinically (24% sensitivity at 80% specificity) [51].** |
|  |  |  |  | **Screening performance of serum PAPP-A is poor for IUGR [52].** |
| 174 | FMNL3 | formin-like 3 | No results in PubMed | none |
| 175 | LILRA2 | leukocyte immunoglobulin-like receptor, subfamily A (with TM domain), member 2 | No results in PubMed | none |
| 176 | SCAMP1 | secretory carrier membrane protein 1 | No results in PubMed | none |
| 177 | A2ML1 | alpha-2-macroglobulin-like 1 | No results in PubMed | none |
| 178 | PKNOX1 | PBX/knotted 1 homeobox 1 | No results in PubMed | none |
| 179 | IL9R | interleukin 9 receptor | No results in PubMed | none |

References

1. Kim YS, Hori M, Yasuda K, Ozaki H (2005) Differences in the gestational pattern of mRNA expression of the Rnd family in rat and human myometria. Comp Biochem Physiol A Mol Integr Physiol 142: 410-5.
2. Lartey J, Gampel A, Pawade J, Mellor H, Bernal AL (2006) Expression of RND proteins in human myometrium. Biol Reprod 75: 452-61.
3. Johnson MP, Brennecke SP, East CE, Dyer TD, Roten LT, et al. (2013) Genetic dissection of the pre-eclampsia susceptibility locus on chromosome 2q22 reveals shared novel risk factors for cardiovascular disease. Mol Hum Reprod 19: 423-37.
4. Gascoin-Lachambre G, Buffat C, Rebourcet R, Chelbi ST, Rigourd V, et al. (2010) Cullins in human intra-uterine growth restriction: expressional and epigenetic alterations. Placenta 31: 151-7.
5. Vaiman D, Mondon F, Garcès-Duran A, Mignot TM, Robert B, et al. (2005) Hypoxia-activated genes from early placenta are elevated in preeclampsia, but not in Intra-Uterine Growth Retardation. BMC genomics 6: 111.
6. Rehman KS, Yin S, Mayhew BA, Word RA, Rainey WE (2003) Human myometrial adaptation to pregnancy: cDNA

microarray gene expression profiling of myometrium from non-pregnant and pregnant women. Mol Hum Reprod 9: 681-700.

# Duncan WC, Shaw JL, Burgess S, McDonald SE, Critchley HO, Horne AW (2011) Ectopic pregnancy as a model to identify endometrial genes and signaling pathways important in decidualization and regulated by local trophoblast. PloS one 6: e23595.

# White L, Suganthini G, Friis R, Dharmarajan A, Charles A (2009) Expression of secreted frizzled-related protein 4 in the primate placenta. Reprod Biomed Online 18: 104-10.

# Farina A, Volinia S, Arcelli D, Francioso F, Desanctis P, et al. (2006) Evidence of genetic underexpression in chorionic villi samples of euploid fetuses with increased nuchal translucency at 10–11 weeks' gestation. Prenat Diagn 26: 128-33.

1. Zhang Z, Zhang L, Zhang L, Jia L, Wang P, Gao Y (2013) Association of Wnt2 and sFRP4 expression in the third trimester placenta in women with severe preeclampsia. Reprod Sci 20: 981-9.
2. Huang X, Hao C, Shen X, Liu X, Shan Y, et al. (2013) Differences in the transcriptional profiles of human cumulus cells isolated from MI and MII oocytes of patients with polycystic ovary syndrome. Reproduction 145: 597-608.
3. Slane BG, Aykin-Burns N, Smith BJ, Kalen AL, Goswami PC, et al. (2006) Mutation of succinate dehydrogenase subunit C results in increased O2·−, oxidative stress, and genomic instability. Cancer res 66: 7615-20.
4. Emdad L, Das SK, Dasgupta S, Hu B, Sarkar D, Fisher PB (2013) AEG-1/MTDH/LYRIC: signaling pathways, downstream genes, interacting proteins, and regulation of tumor angiogenesis. Adv Cancer Res 120: 75-111.
5. [Lee SG](http://www.ncbi.nlm.nih.gov/pubmed?term=Lee SG%5BAuthor%5D&cauthor=true&cauthor_uid=23889986), [Kang DC](http://www.ncbi.nlm.nih.gov/pubmed?term=Kang DC%5BAuthor%5D&cauthor=true&cauthor_uid=23889986), [DeSalle R](http://www.ncbi.nlm.nih.gov/pubmed?term=DeSalle R%5BAuthor%5D&cauthor=true&cauthor_uid=23889986), [Sarkar D](http://www.ncbi.nlm.nih.gov/pubmed?term=Sarkar D%5BAuthor%5D&cauthor=true&cauthor_uid=23889986), [Fisher PB](http://www.ncbi.nlm.nih.gov/pubmed?term=Fisher PB%5BAuthor%5D&cauthor=true&cauthor_uid=23889986) (2013) AEG-1/MTDH/LYRIC, the beginning: initial cloning, structure, expression profile, and regulation of expression. [Adv Cancer Res](http://www.ncbi.nlm.nih.gov/pubmed/23889986) 120: 1-38.
6. Nakamura H, Jasper MJ, Hull ML, Aplin JD, Robertson SA (2012) Macrophages regulate expression of α1, 2-fucosyltransferase genes in human endometrial epithelial cells. Mol Hum Reprod 18: 204-15.
7. [Asvold BO](http://www.ncbi.nlm.nih.gov/pubmed?term=Asvold BO%5BAuthor%5D&cauthor=true&cauthor_uid=24575980), [Eskild A](http://www.ncbi.nlm.nih.gov/pubmed?term=Eskild A%5BAuthor%5D&cauthor=true&cauthor_uid=24575980), [Vatten LJ](http://www.ncbi.nlm.nih.gov/pubmed?term=Vatten LJ%5BAuthor%5D&cauthor=true&cauthor_uid=24575980) (2014) Human chorionic gonadotropin, angiogenic factors, and preeclampsia risk: a nested case-control study. [Acta Obstet Gynecol Scand](http://www.ncbi.nlm.nih.gov/pubmed/24575980) 93: 454-62.
8. [Karahasanovic A](http://www.ncbi.nlm.nih.gov/pubmed?term=Karahasanovic A%5BAuthor%5D&cauthor=true&cauthor_uid=24176962), [Sørensen S](http://www.ncbi.nlm.nih.gov/pubmed?term=Sørensen S%5BAuthor%5D&cauthor=true&cauthor_uid=24176962), [Nilas L](http://www.ncbi.nlm.nih.gov/pubmed?term=Nilas L%5BAuthor%5D&cauthor=true&cauthor_uid=24176962) (2014) First trimester pregnancy-associated plasma protein A and human chorionic gonadotropin-beta in early and late pre-eclampsia. [Clin Chem Lab Med](http://www.ncbi.nlm.nih.gov/pubmed/24176962) 52: 521-5.
9. [Chambers AE](http://www.ncbi.nlm.nih.gov/pubmed?term=Chambers AE%5BAuthor%5D&cauthor=true&cauthor_uid=23245345), [Griffin C](http://www.ncbi.nlm.nih.gov/pubmed?term=Griffin C%5BAuthor%5D&cauthor=true&cauthor_uid=23245345), [Naif SA](http://www.ncbi.nlm.nih.gov/pubmed?term=Naif SA%5BAuthor%5D&cauthor=true&cauthor_uid=23245345), [Mills I](http://www.ncbi.nlm.nih.gov/pubmed?term=Mills I%5BAuthor%5D&cauthor=true&cauthor_uid=23245345), [Mills WE](http://www.ncbi.nlm.nih.gov/pubmed?term=Mills WE%5BAuthor%5D&cauthor=true&cauthor_uid=23245345), et al**.** (2012) Quantitative ELISAs for serum soluble LHCGR and hCG-LHCGR complex: potential diagnostics in first trimester pregnancy screening for stillbirth, Down's syndrome, preterm delivery and preeclampsia. [Reprod Biol Endocrinol](http://www.ncbi.nlm.nih.gov/pubmed/23245345) 10: 113.
10. Lorenzi T, Turi A, Crescimanno C, Morroni M, Castellucci M, et al. (2010). Syndecan expressions in the human amnion and chorionic plate. Eur J Histochem 54: e42.
11. Ishiguro K, Kadomatsu K, Kojima T, Muramatsu H, Nakamura E, et al. (2000) Syndecan-4 deficiency impairs the fetal vessels in the placental labyrinth. Dev Dyn 219: 539-44.
12. Crescimanno C, Marzioni D, Paradinas FJ, Schrurs B, Mühlhauser J, et al. (1999) Expression pattern alterations of syndecans

and glypican-1 in normal and pathological trophoblast. J Pathol 189: 600-8.

1. Chui A, Zainuddin N, Rajaraman G, Murthi P, Brennecke SP, et al. (2012) Placental syndecan expression is altered in human idiopathic fetal growth restriction. [Am J Pathol](http://www.amjpathol.org/) 180: 693-702.
2. Wathlet S, Adriaenssens T, Segers I, Verheyen G, Van de Velde H, et al. (2011) Cumulus cell gene expression predicts better cleavage-stage embryo or blastocyst development and pregnancy for ICSI patients. [Hum Reprod](http://www.oxfordjournals.org/humrep/about.html) 26: 1035-51.
3. Suga N, Sugimura M, Koshiishi T, Yorifuji T, Makino S, Takeda S (2012) Heparin/heparan sulfate/CD44-v3 enhances cell migration in term placenta-derived immortalized human trophoblast cells. Biol Reprod 86: 134.
4. Schanz A, Baston-Bust D, Krussel JS, Heiss C, Janni W, Hess AP (2011) CXCR7 and syndecan-4 are potential receptors for CXCL12 in human cytotrophoblasts. [J Reprod Immunol](http://www.elsevier.com/) 89: 18-25.
5. Sikora M, Ferrer-Admetlla A, Laayouni H, Menendez C, Mayor A, et al. (2009) A variant in the gene FUT9 is associated with susceptibility to placental malaria infection. Hum Mol Genet 18:3136-44.
6. Billard LM, Magdinier F, Lenoir GM, Frappart L, Dante R (2002) MeCP2 and MBD2 expression during normal and pathological growth of the human mammary gland. Oncogene 21: 2704-12.
7. Galetzka D, Weis E, Tralau T, Seidmann L, Haaf T (2007) Sex-specific windows for high mRNA expression of DNA methyltransferases 1 and 3A and methyl-CpG-binding domain proteins 2 and 4 in human fetal gonads. Mol Reprod Dev 74: 233-41.
8. Liu X, Chen Q, Tsai HJ, Wang G, Hong X, et al. (2014) Maternal preconception body mass index and offspring cord blood DNA methylation: Exploration of early life origins of disease. Environ Mol Mutagen 55: 223-30.
9. DeLoia JA, Burlingame JM, Krasnow JS (1997) Differential expression of G1 cyclins during human placentogenesis. Placenta 18: 9-16.
10. Meri S (2013) Complement activation in diseases presenting with thrombotic microangiopathy. Eur J Intern Med 24: 496-502.
11. Graul-Neumann LM, Kienitz T, Robinson PN, Baasanjav S, Karow B, et al. (2010) Marfan syndrome with neonatal progeroid syndrome-like lipodystrophy associated with a novel frameshift mutation at the 3´terminus of the FBN1-gene. Am J Med Genet A 152A: 2749-55.
12. Neale DM, Mor G (2005) The role of FAS mediated apoptosis in preeclampsia. J Perinat Med 33: 471-7.
13. Whitley GS, Dash PR, Ayling LJ, Prefumo F, Thilaganathan B, et al. (2007) Increased apoptosis in first trimester extravillous trophoblasts from pregnancies at higher risk of developing preeclampsia. Am J Pathol 170: 1903-9.
14. Sziller I, Nguyen D, Halmos A, Hupuczi P, Papp Z, et al. (2005) An A > G polymorphism at position -670 in the FAS (TNFRSF6) gene in pregnant women with pre-eclampsia and intrauterine growth restriction. Mol Hum Reprod 11: 207-10.
15. [Robinson R](http://www.ncbi.nlm.nih.gov/pubmed?term=Robinson R%5BAuthor%5D&cauthor=true&cauthor_uid=19716115), [Hsu CD](http://www.ncbi.nlm.nih.gov/pubmed?term=Hsu CD%5BAuthor%5D&cauthor=true&cauthor_uid=19716115), [Chesebro AL](http://www.ncbi.nlm.nih.gov/pubmed?term=Chesebro AL%5BAuthor%5D&cauthor=true&cauthor_uid=19716115), [Nguyen J](http://www.ncbi.nlm.nih.gov/pubmed?term=Nguyen J%5BAuthor%5D&cauthor=true&cauthor_uid=19716115), [Ali N](http://www.ncbi.nlm.nih.gov/pubmed?term=Ali N%5BAuthor%5D&cauthor=true&cauthor_uid=19716115), et al.  (2009) A single-nucleotide polymorphism (-670) of the maternal FAS gene is associated with intrauterine growth restriction. Am J Obstet Gynecol 201: 620.
16. Ciarmela P, Boschi S, Bloise E, Marozio L, Benedetto C, et al. (2010) Polymorphisms of FAS and FAS ligand genes in preeclamptic women. Eur J Obstet Gynecol Reprod Biol 148: 144-6.
17. Collares CV, Evangelista AF, Xavier DJ, Takahashi P, Almeida R, et al. (2013) Transcriptome meta-analysis of peripheral lymphomononuclear cells indicates that gestational diabetes is closer to type 1 diabetes than to type 2 diabetes mellitus. Mol Biol Rep 40: 5351-8.
18. Han JY, Kim YS, Cho GJ, Roh GS, Kim HJ, et al. (2006) Altered gene expression of caspase-10, death receptor-3 and IGFBP-3 in preeclamptic placentas. Mol Cells 22: 168-74.
19. Fong PY, Xue WC, Ngan HY, Chiu PM, Chan KY, et al. (2006) Caspase activity is downregulated in choriocarcinoma: a cDNA array differential expression study. J Clin Pathol 59: 179-83.
20. Allegra A, Marino A, Peregrin PC, Lama A, García-Segovia Á, et al. (2012) Endometrial expression of selected genes in patients achieving pregnancy spontaneously or after ICSI and patients failing at least two ICSI cycles. Reprod Biomed Online 25: 481-91.
21. Assou S, Haouzi D, Mahmoud K, Aouacheria A, Guillemin Y, et al. (2008) A non-invasive test for assessing embryo potential by gene expression profiles of human cumulus cells: a proof of concept study. [Mol Hum Reprod](http://molehr.oxfordjournals.org/) 14: 711-19.
22. Hamamah S, Fallet C (2010) Gene expression profile of human cumulus cells: clinical applications for IVF. J Gynecol Obstet Biol Reprod 39: 5-7.
23. Schroeder JK, Kessler CA, Handwerger S (2011) Critical role for TWIST1 in the induction of human uterine decidualization. Endocrinology 152: 4368-76.
24. O'Brien M, Morrison JJ, Smith TJ (2008) Upregulation of PSCDBP, TLR2, TWIST1, FLJ35382, EDNRB, and RGS12 gene expression in human myometrium at labor. Reprod Sci 15: 382-93.
25. Pirinen E, Soini Y (2014) A survey of zeb1, twist and claudin 1 and 4 expressions during placental development and disease. APMIS 122: 530-8.
26. Ciurea AV, Toader C (2009) Genetics of craniosynostosis: review of the literature. J Med Life, 2: 5-17.
27. Spaggiari E, Aboura A, Sinico M, Mabboux P, Dupont C, et al. (2012) Prenatal diagnosis of a 7p15-p21 deletion encompassing the TWIST1 gene involved in Saethre–Chotzen syndrome. Eur J Med Genet 55: 498-501.
28. Ng YH, Zhu H, Leung PC (2011) Twist modulates human trophoblastic cell invasion via regulation of N-cadherin. Endocrinology 153: 925-36.
29. Li M, Erickson SW, Hobbs CA, Li J, Tang X, et al. (2014) Detecting Maternal-Fetal Genotype Interactions Associated With Conotruncal Heart Defects: A Haplotype-Based Analysis With Penalized Logistic Regression. Genet Epidemiol 38: 198-208.
30. [Spencer K](http://www.ncbi.nlm.nih.gov/pubmed?term=Spencer K%5BAuthor%5D&cauthor=true&cauthor_uid=17149788), [Cowans NJ](http://www.ncbi.nlm.nih.gov/pubmed?term=Cowans NJ%5BAuthor%5D&cauthor=true&cauthor_uid=17149788), [Chefetz I](http://www.ncbi.nlm.nih.gov/pubmed?term=Chefetz I%5BAuthor%5D&cauthor=true&cauthor_uid=17149788), [Tal J](http://www.ncbi.nlm.nih.gov/pubmed?term=Tal J%5BAuthor%5D&cauthor=true&cauthor_uid=17149788), [Meiri H](http://www.ncbi.nlm.nih.gov/pubmed?term=Meiri H%5BAuthor%5D&cauthor=true&cauthor_uid=17149788) (2007) First-trimester maternal serum PP-13, PAPP-A and second-trimester uterine artery Doppler pulsatility index as markers of pre-eclampsia. [Ultrasound Obstet Gynecol](http://www.ncbi.nlm.nih.gov/pubmed/17149788) 29: 128-34.
31. Vandenberghe G, Mensink I, Twisk JWR, Blankenstein MA, Heijboer AC, van Vugt JMG (2011) First trimester screening

for intra-uterine growth restriction and early-onset pre-eclampsia. Prenat Diagn 31: 955-61.
