# Supplementary material for: First Trimester Screening of Circulating C19MC microRNAs Can Predict Subsequent Onset of Gestational Hypertension
Source: PLoS One. 2014 Dec 15;9(12):e113735. doi: 10.1371/journal.pone.0113735 (PMC4266496; doi:10.1371/journal.pone.0113735)
Supplement: S3 Table — Function of target genes of miR-518b in relation to pregnancy. (DOCX) [file pone.0113735.s003.docx]

**Table S3. Function of target genes of differentially expressed extracellular C19MC microRNAs in patients developing gestational hypertension in relation to pregnancy**

**miR-518b**

| **No.** | **GENE** | **GENE full name** | **Total number of references,** | **The role in gestation** |
| --- | --- | --- | --- | --- |
|  | **official symbol** |  | **list of references in PubMed (humans)** |  |
| 1 | RAP1B | RAP1B, member of RAS oncogene family | No results in PubMed | none |
| 2 | TOLLIP | toll interacting protein | 2 [1,2] | The spatial expression of Tollip (negative regulator Toll-interacting protein) in human placental tissues at different stages of gestation may play an important role in the pathophysiology of preeclampsia [1]. |
|  |  |  |  | Upregulation of TOLLIP in vitamin D-treated human myometrial smooth muscle cells in relation to infection during pregnancy that triggers inflammation, which can increase myometrial contractions and the risk of premature labor and delivery [2]. |
| 3 | SLCO4C1 | solute carrier organic anion transporter family, member 4C1 | 1 [3] | SNPs and haplotypes in SLCO4C1 in blacks are significantly associated with preeclampsia [3]. |
| 4 | PRDX6 | peroxiredoxin 6 | 5 [4-8] | Differential expression of PRDX6 protein in placenta of Down's syndrome was observed [4, 5]. PRDX6 has been suggested as a new screening marker for Down syndrome [4]. |
|  |  |  |  | PRDX6 decreased in highly purified cytotrophoblasts in pre-eclampsia [6]. |
|  |  |  |  | Differential expression of PRDX6 between gestational diabetes mellitus patients and normal glucose tolerance pregnant women in maternal adipose tissue was demonstrated [7]. |
|  |  |  |  | Alterations in PRDX6 protein expression in placentas from women with intrahepatic cholestasis of pregnancy, which usually occurs in the third trimester and is associated with increased risks in fetal complications, were presented [8]. |
| 5 | QKI | QKI, KH domain containing, RNA binding | No results in PubMed | none |
| 6 | TSN | translin | No results in PubMed | none |
| 7 | PARP11 | poly(ADP-ribose) polymerase family, member 11 | No results in PubMed | none |
| 8 | ZNF282 | zinc finger protein 282 | No results in PubMed | none |
| 9 | VAPB | VAMP (vesicle-associated membrane protein)-associated protein B and C | No results in PubMed | none |
| 10 | MYO10 | myosin X | No results in PubMed | none |
| 11 | CPEB1 | cytoplasmic polyadenylation element binding protein 1 | No results in PubMed | none |
| 12 | RNF150 | ring finger protein 150 | No results in PubMed | none |
| 13 | C1QTNF2 | C1q and tumor necrosis factor related protein 2 | No results in PubMed | none |
| 14 | HMP19 | HMP19 protein | No results in PubMed | none |
| 15 | ZNF608 | zinc finger protein 608 | No results in PubMed | none |
| 16 | FBXO3 | F-box protein 3 | No results in PubMed | none |
| 17 | PLEKHH2 | pleckstrin homology domain containing, family H (with MyTH4 domain) member 2 | No results in PubMed | none |
| 18 | NCAPH | non-SMC condensin I complex, subunit H | No results in PubMed | none |
| 19 | HNRNPUL1 | heterogeneous nuclear ribonucleoprotein U-like 1 | No results in PubMed | none |
| 20 | ZNF185 | zinc finger protein 185 (LIM domain) | No results in PubMed | none |
| 21 | CTNNBIP1 | catenin, beta interacting protein 1 | No results in PubMed | none |
| 22 | PRUNE | prune homolog (Drosophila) | No results in PubMed | none |
| 23 | PKNOX1 | PBX/knotted 1 homeobox 1 | No results in PubMed | none |
| 24 | C15orf54 | chromosome 15 open reading frame 54 | No results in PubMed | none |
| 25 | ZNF827 | zinc finger protein 827 | No results in PubMed | none |
| 26 | MYOD1 | myogenic differentiation 1 | No results in PubMed | none |
| 27 | FRMD6 | FERM domain containing 6 | No results in PubMed | none |
| 28 | WNK1 | WNK lysine deficient protein kinase 1 | No results in PubMed | none |
| 29 | ORC5 | origin recognition complex, subunit 5 | No results in PubMed | none |
| 30 | ZNF554 | zinc finger protein 554 | No results in PubMed | none |
| 31 | OTP | orthopedia homeobox | No results in PubMed | none |
| 32 | ZNF264 | zinc finger protein 264 | No results in PubMed | none |
| 33 | C1orf43 | chromosome 1 open reading frame 43 | No results in PubMed | none |
| 34 | DGCR8 | DiGeorge syndrome critical region gene 8 | No results in PubMed | none |
| 35 | SPATS2L | spermatogenesis associated, serine-rich 2-like | No results in PubMed | none |
| 36 | LPIN1 | lipin 1 | No results in PubMed | none |
| 37 | CCDC69 | coiled-coil domain containing 69 | No results in PubMed | none |
| 38 | PTPRU | protein tyrosine phosphatase, receptor type, U | No results in PubMed | none |
| 39 | C5orf46 | chromosome 5 open reading frame 46 | No results in PubMed | none |
| 40 | CXADR | coxsackie virus and adenovirus receptor | 1 [9] | CXADR protein showed no overexpression in DS compared to controls suggesting that this protein is not involved in abnormal development of fetal DS brain and that DS phenotype can not be simply explained by the gene dosage effect hypothesis [9]. |
| 41 | MR1 | major histocompatibility complex, class I-related | No results in PubMed | none |
| 42 | CD5 | CD5 molecule | No results in PubMed | none |

References

1. Zhang L, Yang H (2012) Expression and localization of TLR4 and its negative regulator Tollip in the placenta of early-onset and

late-onset preeclampsia. Hypertens Pregnancy 31: 218-27.

2. [Thota C](http://www.ncbi.nlm.nih.gov/pubmed?term=Thota%20C%5BAuthor%5D&cauthor=true&cauthor_uid=23012315), [Farmer T](http://www.ncbi.nlm.nih.gov/pubmed?term=Farmer%20T%5BAuthor%5D&cauthor=true&cauthor_uid=23012315), [Garfield RE](http://www.ncbi.nlm.nih.gov/pubmed?term=Garfield%20RE%5BAuthor%5D&cauthor=true&cauthor_uid=23012315), [Menon R](http://www.ncbi.nlm.nih.gov/pubmed?term=Menon%20R%5BAuthor%5D&cauthor=true&cauthor_uid=23012315), [Al-Hendy A](http://www.ncbi.nlm.nih.gov/pubmed?term=Al-Hendy%20A%5BAuthor%5D&cauthor=true&cauthor_uid=23012315) (2012) Vitamin D elicits anti-inflammatory response, inhibits

contractile-associated proteins, and modulates Toll-like receptors in human myometrial cells. Reprod Sci 20(4): 463-75.

3. Morrison AC, Srinivas SK, Elovitz MA, Puschett JB (2010) Genetic variation in solute carrier genes is associated with

preeclampsia. Am J Obstet Gynecol 203(5): 491.e1-491.e13.

4. Yan LY, Sun CJ, Wang X, Chen Y, Zhang WY (2011) Screen and identify of differential proteins expressed in the placenta of

Down's syndrome. Zhonghua Fu Chan Ke Za Zhi 46: 161-6.

5. Sun CJ, Yan LY, Wang W, Yu S, Wang X, Zhang WY (2011) Proteomic analysis of the alteration of protein expression in the

placenta of Down syndrome. Chin Med J (Engl) 124: 3738.

6. Johnstone ED, Sawicki G, Guilbert L, Winkler-Lowen B, Cadete VJ, Morrish DW (2011) Differential proteomic analysis of

highly purified placental cytotrophoblasts in pre-eclampsia demonstrates a state of increased oxidative stress and reduced

cytotrophoblast antioxidant defense. Proteomics 11: 4077-84.

7. Oliva K, Barker G, Rice GE, Bailey MJ, Lappas M (2013) 2D-DIGE to identify proteins associated with gestational diabetes in

omental adipose tissue. J Endocrinol 218: 165-78.

8. Zhang T, Guo Y, Guo X, Zhou T, Chen D, et al. (2013) Comparative Proteomics Analysis of Placenta from Pregnant Women

with Intrahepatic Cholestasis of Pregnancy. PloS one 8: e83281.

9. [Cheon MS](http://www.ncbi.nlm.nih.gov/pubmed?term=Cheon%20MS%5BAuthor%5D&cauthor=true&cauthor_uid=12836057), [Shim KS](http://www.ncbi.nlm.nih.gov/pubmed?term=Shim%20KS%5BAuthor%5D&cauthor=true&cauthor_uid=12836057), [Kim SH](http://www.ncbi.nlm.nih.gov/pubmed?term=Kim%20SH%5BAuthor%5D&cauthor=true&cauthor_uid=12836057), [Hara A](http://www.ncbi.nlm.nih.gov/pubmed?term=Hara%20A%5BAuthor%5D&cauthor=true&cauthor_uid=12836057), [Lubec G](http://www.ncbi.nlm.nih.gov/pubmed?term=Lubec%20G%5BAuthor%5D&cauthor=true&cauthor_uid=12836057) (2003) Protein levels of genes encoded on chromosome 21 in fetal Down

syndrome brain: Challenging the genedosage effect hypothesis (Part IV). [Amino Acids](http://www.ncbi.nlm.nih.gov/pubmed/?term=Protein+levels+of+genes+encoded+on+chromosome+21+in+fetal+Down+syndrome+brain%3A+challenging+the+gene+dosage+effect+hypothesis+(Part+IV)) 25: 41-7.
