# Supplementary material for: First Trimester Screening of Circulating C19MC microRNAs Can Predict Subsequent Onset of Gestational Hypertension
Source: PLoS One. 2014 Dec 15;9(12):e113735. doi: 10.1371/journal.pone.0113735 (PMC4266496; doi:10.1371/journal.pone.0113735)
Supplement: S4 Table — Function of target genes of miR-520h in relation to pregnancy. (DOCX) [file pone.0113735.s004.docx]

**Table S4. Function of target genes of differentially expressed extracellular C19MC microRNAs in patients developing gestational hypertension in relation to pregnancy**

**miR-520h**

| **No.** | **GENE** | **GENE full name** | **Total number of references,** | **The role in gestation** |
| --- | --- | --- | --- | --- |
|  | **official symbol** |  | **list of references in PubMed (humans)** |  |
| 1 | TBC1D15 | TBC1 domain family, member 15 | No results in PubMed | none |
| 2 | AAK1 | AP2 associated kinase 1 | No results in PubMed | none |
| 3 | RRAGD | Ras-related GTP binding D | No results in PubMed | none |
| 4 | SMOC2 | SPARC related modular calcium binding 2 | No results in PubMed | none |
| 5 | SORL1 | sortilin-related receptor, L(DLR class) A repeats containing | No results in PubMed | none |
| 6 | DNM1L | dynamin 1-like | No results in PubMed | none |
| 7 | TNRC6B | trinucleotide repeat containing 6B | No results in PubMed | none |
| 8 | CPSF6 | cleavage and polyadenylation specific factor 6, 68kDa | No results in PubMed | none |
| 9 | SESTD1 | SEC14 and spectrin domains 1 | No results in PubMed | none |
| 10 | NOX4 | NADPH oxidase 4 | 2 [1, 2] | NOX4 is a potential new biomarker candidate for preeclampsia. NOX4 is down-regulated in placental villous tissue from patients with severe preeclampsia in comparison to normal healthy pregnancies [1]. |
|  |  |  |  | Several genes involved in vasoregulation and endothelial homeostasis showed modified expression, including EDN1, APLN, NOX4, and CBS when the effects of plasma from women with preeclamptic pregnancies on the transcriptome of an immortalized human umbilical vein endothelial cell line were studied [2]. |
| 11 | ELK4 | ELK4, ETS-domain protein (SRF accessory protein 1) | No results in PubMed | none |
| 12 | VAV3 | vav 3 guanine nucleotide exchange factor | 1 [3] | VAV3 gene had expression pattern that was significantly different between gestational diabetes mellitus and control samples in Chinese women, as determined by both microarray and Q-RT-PCR assays [3]. |
| 13 | TNRC6A | trinucleotide repeat containing 6A | No results in PubMed | none |
| 14 | ETF1 | eukaryotic translation termination factor 1 | No results in PubMed | none |
| 15 | KPNA3 | karyopherin alpha 3 (importin alpha 4) | No results in PubMed | none |
| 16 | VLDLR | very low density lipoprotein receptor | No results in PubMed | none |
| 17 | PIAS2 | protein inhibitor of activated STAT, 2 | No results in PubMed | none |
| 18 | BET1 | blocked early in transport 1 homolog (S. cerevisiae) | No results in PubMed | none |
| 19 | ZFYVE20 | zinc finger, FYVE domain containing 20 | No results in PubMed | none |
| 20 | SPRY4 | sprouty homolog 4 (Drosophila) | No results in PubMed | none |
| 21 | FLRT3 | fibronectin leucine rich transmembrane protein 3 | No results in PubMed | none |
| 22 | CYB5B | cytochrome b5 type B (outer mitochondrial membrane) | No results in PubMed | none |
| 23 | SEMA5A | sema domain, seven thrombospondin repeats (type 1 and type 1-like), transmembrane domain (TM) and short cytoplasmic domain, (semaphorin) 5A | No results in PubMed | none |
| 24 | KLF12 | Kruppel-like factor 12 | 1 [4] | KLF12 is a negative regulator of human endometrial stromal cell decidualization [4]. |
| 25 | BAGE5 | B melanoma antigen family, member 5 | No results in PubMed | none |
| 26 | FBN1 | fibrillin 1 | 65 [selection 5-7] | Marfan syndrome, an inherited disorders of connective tissue with major cardiovascular complications, results from mutations in the FBN1 gene, which encodes fibrillin-1, an extracellular matrix component found in structures called microfibrils [5]. |
|  |  |  |  | Marfan syndrome, caused by FBN1 mutations, and polymorphisms in the COL1A1 and TGFB1 genes have been associated with cervical incompetence [6]. |
|  |  |  |  | Stroma in terminal villi exhibits intense labelling for fibrillin-1. The fibrillin-1 villi surface fraction is greater in PE and SLE placentae than in controls [7]. |
| 27 | TCERG1 | transcription elongation regulator 1 | No results in PubMed | none |
| 28 | RNF125 | ring finger protein 125 | No results in PubMed | none |
| 29 | JAZF1 | JAZF zinc finger 1 | 3 [8-10] | Low birthweight might affect the strength of the association of some common variants (HHEX, CDKN2A/2B and JAZF1) with type 2 diabetes [8]. |
|  |  |  |  | Variants in genes HHEX, CDKN2A/2B, JAZF1, and IGF2BP2 were found to interact with prenatal nutrition in relation to type 2 diabetes risk and glucose levels in later life [9]. |
|  |  |  |  | A reduced risk for gestational diabetes was identified among carriers of the JAZF1 rs864745 T allele [10]. |
| 30 | KIAA1826 | KIAA1826 | No results in PubMed | none |
| 31 | CYP20A1 | cytochrome P450, family 20, subfamily A, polypeptide 1 | No results in PubMed | none |
| 32 | EBF3 | early B-cell factor 3 | No results in PubMed | none |
| 33 | KPNA1 | karyopherin alpha 1 (importin alpha 5) | No results in PubMed | none |
| 34 | CAPN2 | calpain 2, (m/II) large subunit | 1 [11] | Significantly aberrant DNA methylation in CAPN2 gene in severe preeclamptic placentas was identified [11]. |
| 35 | TRPC5 | transient receptor potential cation channel, subfamily C, member 5 | 1 [12] | Messenger RNAs encoding TRPC1, TRPC3, TRPC4, TRPC5 and TRPC6 were identified in both first trimester and term placentas [12]. |
| 36 | ANXA4 | annexin A4 | 4 [13-16] | Increased expression of annexin IV during the implantation window plays an important role in the morphological differentiation of the uterus to the receptive state [13]. |
|  |  |  |  | Annexin IV protein is expressed on the basal surface of syncytiotrophoblasts; plasma levels of annexin IV suddenly increase after delivery. Since annexin IV enters the maternal bloodstream just after delivery it might play a role in preventing disseminated intravascular coagulopathy [14]. |
|  |  |  |  | There were no significant differences in plasma annexin IV levels between women with and without antiphospholipid antibodies [15]. |
|  |  |  |  | Significantly different endometrial expression of ANXA4 gene during the window of implantation in natural cycles between patients who achieved a successful pregnancy spontaneously or after subsequent intracytoplasmic sperm injection (ICSI) cycles and patients who did not achieve a pregnancy after at least two failed ICSI cycles [16]. |
| 37 | DIDO1 | death inducer-obliterator 1 | No results in PubMed | none |
| 38 | STK17A | serine/threonine kinase 17a | No results in PubMed | none |
| 39 | KLHL29 | kelch-like 29 (Drosophila) | No results in PubMed | none |
| 40 | PBK | PDZ binding kinase | No results in PubMed | none |
| 41 | TOX3 | TOX high mobility group box family member 3 | No results in PubMed | none |
| 42 | WDR35 | WD repeat domain 35 | No results in PubMed | none |
| 43 | IRAK4 | interleukin-1 receptor-associated kinase 4 | No results in PubMed | none |
| 44 | ARCN1 | archain 1 | No results in PubMed | none |
| 45 | MAP3K2 | mitogen-activated protein kinase kinase kinase 2 | No results in PubMed | none |
| 46 | FLRT2 | fibronectin leucine rich transmembrane protein 2 | No results in PubMed | none |
| 47 | KBTBD3 | kelch repeat and BTB (POZ) domain containing 3 | No results in PubMed | none |
| 48 | ZNF618 | zinc finger protein 618 | No results in PubMed | none |
| 49 | FBXW11 | F-box and WD repeat domain containing 11 | No results in PubMed | none |
| 50 | KCTD18 | potassium channel tetramerisation domain containing 18 | No results in PubMed | none |
| 51 | SCML2 | sex comb on midleg-like 2 (Drosophila) | No results in PubMed | none |
| 52 | CYP7B1 | cytochrome P450, family 7, subfamily B, polypeptide 1 | No results in PubMed | none |
| 53 | ADRBK2 | adrenergic, beta, receptor kinase 2 | No results in PubMed | none |
| 54 | ATPBD4 | ATP binding domain 4 | No results in PubMed | none |
| 55 | DCLK2 | doublecortin-like kinase 2 | No results in PubMed | none |
| 56 | LAMP2 | lysosomal-associated membrane protein 2 | 1 [17] | Autophagy predominantly localized to the syncytiotrophoblast layer and autophagosomes were more frequent in FGR. The regulators LAMP-2, LC3B, Beclin-1, ATG 5, ATG9 and ATG16L1 were all present in villous trophoblast [17]. |
| 57 | MTCH2 | mitochondrial carrier 2 | No results in PubMed | none |
| 58 | JAK1 | Janus kinase 1 | No results in PubMed | none |
| 59 | ZNF318 | zinc finger protein 318 | No results in PubMed | none |
| 60 | SLC31A1 | solute carrier family 31 (copper transporters), member 1 | No results in PubMed | none |
| 61 | HIF1A | hypoxia inducible factor 1, alpha subunit (basic helix-loop-helix transcription factor) | 133 [selection 18,19] | HIF-1 functions as a master regulator of cellular and systemic homeostatic response to hypoxia by activating transcription of many genes, including those involved in energy metabolism, angiogenesis, apoptosis, and other genes whose protein products increase oxygen delivery or facilitate metabolic adaptation to hypoxia. HIF-1 thus plays an essential role in embryonic vascularization, tumor angiogenesis and pathophysiology of ischemic disease. Levels of HIF-1α in the placentas of pre-eclamptic patients were found to be higher than the levels in the placentas of healthy pregnant women [18, 19]. |
| 62 | RGPD6 | RANBP2-like and GRIP domain containing 6 | No results in PubMed | none |
| 63 | RGPD8 | RANBP2-like and GRIP domain containing 8 | No results in PubMed | none |
| 64 | RGPD5 | RANBP2-like and GRIP domain containing 5 | No results in PubMed | none |
| 65 | ACAD8 | acyl-CoA dehydrogenase family, member 8 | No results in PubMed | none |
| 66 | KLB | klotho beta | No results in PubMed | none |
| 67 | CHUK | conserved helix-loop-helix ubiquitous kinase | No results in PubMed | none |
| 68 | PRICKLE2 | prickle homolog 2 (Drosophila) | No results in PubMed | none |
| 69 | RND3 | Rho family GTPase 3 | 3 [20-22] | RND1, RND2, RND3 mRNAs were expressed in nonpregnant human myometrial tissues in the study of myometrial contractility in humans [20]. |
|  |  |  |  | A significant increase of RND2 and RND3 protein expression was observed in pregnant relative to nonpregnant myometrium associated with a loss of PPP1R12A phosphorylation [21]. |
|  |  |  |  | The association between UTR-3 RND3 SNP (rs115015150) and risk for preeclampsia and later life cardiovascular disease was revealed in an extended Australian and New Zealand familial cohort [22]. |
| 70 | RFX4 | regulatory factor X, 4 (influences HLA class II expression) | No results in PubMed | none |
| 71 | PFN2 | profilin 2 | No results in PubMed | none |
| 72 | CAPN7 | calpain 7 | No results in PubMed | none |
| 73 | PPP6C | protein phosphatase 6, catalytic subunit | No results in PubMed | none |
| 74 | BAGE4 | B melanoma antigen family, member 4 | No results in PubMed | none |
| 75 | MAP3K9 | mitogen-activated protein kinase kinase kinase 9 | No results in PubMed | none |
| 76 | GABRG1 | gamma-aminobutyric acid (GABA) A receptor, gamma 1 | No results in PubMed | none |
| 77 | EIF4G2 | eukaryotic translation initiation factor 4 gamma, 2 | No results in PubMed | none |
| 78 | SALL1 | sal-like 1 (Drosophila) | No results in PubMed | none |
| 79 | ZNF280B | zinc finger protein 280B | No results in PubMed | none |
| 80 | C3orf55 | chromosome 3 open reading frame 55 | No results in PubMed | none |
| 81 | EIF4E | eukaryotic translation initiation factor 4E | 2 [23-24] | EIF4E prevents final extravillous trophoblast cell differentiation and supports placental cell proliferation and survival [23]. |
|  |  |  |  | Leptin dose-dependently stimulates in human trophoblastic cells the phosphorylation and activation of the translation initiation factor EIF4E as well as the phosphorylation of the EIF4E binding protein EIF4EBP1 (PHAS-I), which releases EIF4E to form active complexes [24]. |
| 82 | CNTLN | centlein, centrosomal protein | No results in PubMed | none |
| 83 | MGEA5 | meningioma expressed antigen 5 (hyaluronidase) | No results in PubMed | none |
| 84 | GNA14 | guanine nucleotide binding protein (G protein), alpha 14 | 1 [25] | The high expression of GNA14 in severe preeclamptic placentas may imply its importance in severe preeclampsia pregnancies as in the other hypertension-related disorders [25]. |
| 85 | PLEKHA8 | pleckstrin homology domain containing, family A (phosphoinositide binding specific) member 8 | No results in PubMed | none |
| 86 | VAMP4 | vesicle-associated membrane protein 4 | No results in PubMed | none |
| 87 | EPB41L2 | erythrocyte membrane protein band 4.1-like 2 | No results in PubMed | none |
| 88 | PAK2 | p21 protein (Cdc42/Rac)-activated kinase 2 | 2 [26-27] | PAK isoforms (PAK1, PAK2, and PAK3) have the potential to regulate uterine contractility and/or load-bearing during human pregnancy [26]. |
|  |  |  |  | PAK2 mRNA and p-PAK2 immunoreactivity showed a similar expression pattern in normal first trimester placentas and gestational trophoblastic neoplasia [27]. |
| 89 | MAP9 | microtubule-associated protein 9 | No results in PubMed | none |
| 90 | SCML1 | sex comb on midleg-like 1 (Drosophila) | No results in PubMed | none |
| 91 | DLG2 | discs, large homolog 2 (Drosophila) | No results in PubMed | none |
| 92 | GNS | glucosamine (N-acetyl)-6-sulfatase | No results in PubMed | none |
| 93 | ABHD2 | abhydrolase domain containing 2 | No results in PubMed | none |
| 94 | THAP2 | THAP domain containing, apoptosis associated protein 2 | No results in PubMed | none |
| 95 | USP9X | ubiquitin specific peptidase 9, X-linked | No results in PubMed | none |
| 96 | SERINC1 | serine incorporator 1 | No results in PubMed | none |
| 97 | MOSPD1 | motile sperm domain containing 1 | No results in PubMed | none |
| 98 | SH3TC2 | SH3 domain and tetratricopeptide repeats 2 | No results in PubMed | none |
| 99 | NR4A2 | nuclear receptor subfamily 4, group A, member 2 | 1 [28] | Altered expression of NR4A2 transcript in non-pregnant multiple sclerosis patients with respect to non-pregnant healthy controls was observed [28]. |
| 100 | WNK1 | WNK lysine deficient protein kinase 1 | No results in PubMed | none |
| 101 | BAGE2 | B melanoma antigen family, member 2 | No results in PubMed | none |
| 102 | VWA5A | von Willebrand factor A domain containing 5A | No results in PubMed | none |
| 103 | BAGE3 | B melanoma antigen family, member 3 | No results in PubMed | none |
| 104 | ST8SIA3 | ST8 alpha-N-acetyl-neuraminide alpha-2,8-sialyltransferase 3 | No results in PubMed | none |
| 105 | LRRTM3 | leucine rich repeat transmembrane neuronal 3 | No results in PubMed | none |
| 106 | GSTT2 | glutathione S-transferase theta 2 | No results in PubMed | none |
| 107 | GTF3C3 | general transcription factor IIIC, polypeptide 3, 102kDa | No results in PubMed | none |
| 108 | EEA1 | early endosome antigen 1 | No results in PubMed | none |
| 109 | KIAA2018 | KIAA2018 |  |  |
| 110 | NAP1L5 | nucleosome assembly protein 1-like 5 | No results in PubMed | none |
| 111 | CHD9 | chromodomain helicase DNA binding protein 9 | No results in PubMed | none |
| 112 | CAMK2N1 | calcium/calmodulin-dependent protein kinase II inhibitor 1 | No results in PubMed | none |
| 113 | GBP6 | guanylate binding protein family, member 6 | 1 [29] | GBP6 was highly expressed in human placenta in less muscular women; GBP6 is related to immune system processes and the interferon-γ signaling pathway in particular. Placentas from women with low muscularity are more sensitive to the effects of inflammatory cytokines than those from more muscular women [29]. |
| 114 | IMPAD1 | inositol monophosphatase domain containing 1 | No results in PubMed | none |
| 115 | GOLM1 | golgi membrane protein 1 | No results in PubMed | none |
| 116 | CKS1B | CDC28 protein kinase regulatory subunit 1B | No results in PubMed | none |
| 117 | FMN1 | formin 1 | No results in PubMed | none |
| 118 | TLR5 | toll-like receptor 5 | 11 [selection 30-33] | All placentas expressed transcripts for TLR1-TLR10 [30]. |
|  |  |  |  | Elevated expression of TLR5 and TLR9 in cord blood was associated with decreased doctor diagnosis of atopic dermatitis. Maternal contact with farm animals and cats during pregnancy has a protective effect on the development of atopic dermatitis in early life, which is associated with a lower expression of innate immune receptors at birth [31]. |
|  |  |  |  | Amniotic epithelial cells express functional TLR5, TLR6/2, and TLR4. Activation by TLR5 and TLR6/2 agonists produces IL-6 and IL-8, concomitantly with the activation of NF-κB signaling pathway, matrix metalloproteinase-9 induction, and PTGS2 expression. Specific TLR-mediated functions in human amniotic epithelial cells initiate different immune responses, which ultimately may lead to preterm birth [32]. |
|  |  |  |  | Dysregulated pattern of TLR expression and cytokine production in dendritic cells from preeclamptic patients may limit further activation by TLR engagement [33]. |
| 119 | DCTN4 | dynactin 4 (p62) | No results in PubMed | none |
| 120 | KATNAL2 | katanin p60 subunit A-like 2 | No results in PubMed | none |
| 121 | SLCO1B3 | solute carrier organic anion transporter family, member 1B3 | No results in PubMed | none |
| 122 | RNF216 | ring finger protein 216 | No results in PubMed | none |
| 123 | HECTD2 | HECT domain containing 2 | No results in PubMed | none |
| 124 | GCH1 | GTP cyclohydrolase 1 | 4 [selection 34-35] | GTPCH activity was low in the human placenta and became negligible after the second trimester [34]. |
|  |  |  |  | Prenatal dopaminergic replacement therapy in autosomal recessive guanosine triphosphate cyclohydrolase 1 deficiency without hyperphenylalaninemia was reported [35]. |
| 125 | SATB2 | SATB homeobox 2 | No results in PubMed | none |
| 126 | C14orf105 | chromosome 14 open reading frame 105 | No results in PubMed | none |
| 127 | FAM46A | family with sequence similarity 46, member A | No results in PubMed | none |
| 128 | RNF145 | ring finger protein 145 | No results in PubMed | none |
| 129 | BRWD1 | bromodomain and WD repeat domain containing 1 | No results in PubMed | none |
| 130 | LRRFIP2 | leucine rich repeat (in FLII) interacting protein 2 | No results in PubMed | none |
| 131 | ZFP42 | zinc finger protein 42 homolog (mouse) | No results in PubMed | none |
| 132 | PHF14 | PHD finger protein 14 | 2 [36, 37] | Copy number variations (CNVs) of chromosome 7p21.3 region are associated with Dandy-Walker malformations which may be due to haploinsufficiency or overexpression of NDUFA4 and PHF14 genes [36]. |
|  |  |  |  | The critical region associated with Dandy-Walker malformation is restricted to 7p21.3, including the cerebellar disease associated genes NDUFA4 and PHF14 [37]. |
| 133 | LIMK1 | LIM domain kinase 1 | No results in PubMed | none |
| 134 | PRCP | prolylcarboxypeptidase (angiotensinase C) | 1 [38] | A lysosomal prolylcarboxypeptidase cleaves C-terminal amino acids linked to proline in peptides such as angiotension II, III and des-Arg9-bradykinin regulating blood pressure and electrolyte balance. PRCP polymorphism was shown to be associated with increased risk of preeclampsia. The PRCP transcript levels were lower in preeclamptic women than in non-preeclamptic women [38]. |
| 135 | CLCA2 | chloride channel accessory 2 | No results in PubMed | none |
| 136 | SSR1 | signal sequence receptor, alpha | No results in PubMed | none |
| 137 | C19orf75 | chromosome 19 open reading frame 75 | No results in PubMed | none |
| 138 | PLEKHM3 | pleckstrin homology domain containing, family M, member 3 | No results in PubMed | none |
| 139 | SENP1 | SUMO1/sentrin specific peptidase 1 | 1 [39] | A novel cyclic AMP/Epac1/CaMKI signaling cascade promotes GCM1 desumoylation and placental cell fusion [39]. |
| 140 | DDX46 | DEAD (Asp-Glu-Ala-Asp) box polypeptide 46 | No results in PubMed | none |
| 141 | EIF4G3 | eukaryotic translation initiation factor 4 gamma, 3 | No results in PubMed | none |
| 142 | MKLN1 | muskelin 1, intracellular mediator containing kelch motifs | No results in PubMed | none |
| 143 | PIK3R1 | phosphoinositide-3-kinase, regulatory subunit 1 (alpha) | No results in PubMed | none |
| 144 | FAM129A | family with sequence similarity 129, member A | No results in PubMed | none |
| 145 | ZNF81 | zinc finger protein 81 | No results in PubMed | none |
| 146 | MYCN | v-myc myelocytomatosis viral related oncogene, neuroblastoma derived (avian) | 1 [40] | Associations between premature delivery, very low birthweight and risk of neuroblastoma were identified. A weak association was found for hypertension during pregnancy. Several labour and delivery factors were related to an increased risk of neuroblastoma, including threatened miscarriage, anaesthetic during labour (specifically epidural) and caesarean delivery [40]. |
| 147 | FAM89A | family with sequence similarity 89, member A | No results in PubMed | none |
| 148 | AHCTF1 | AT hook containing transcription factor 1 | No results in PubMed | none |
| 149 | NECAB2 | N-terminal EF-hand calcium binding protein 2 | No results in PubMed | none |
| 150 | GDA | guanine deaminase | No results in PubMed | none |
| 151 | CRCT1 | cysteine-rich C-terminal 1 | No results in PubMed | none |
| 152 | LHX8 | LIM homeobox 8 | No results in PubMed | none |
| 153 | DCDC2 | doublecortin domain containing 2 | No results in PubMed | none |
| 154 | LYRM2 | LYR motif containing 2 | No results in PubMed | none |
| 155 | DDX60 | DEAD (Asp-Glu-Ala-Asp) box polypeptide 60 | No results in PubMed | none |
| 156 | NUFIP2 | nuclear fragile X mental retardation protein interacting protein 2 | No results in PubMed | none |
| 157 | SPTLC2 | serine palmitoyltransferase, long chain base subunit 2 | No results in PubMed | none |
| 158 | SEC23IP | SEC23 interacting protein | No results in PubMed | none |
| 159 | TTPAL | tocopherol (alpha) transfer protein-like | No results in PubMed | none |
| 160 | BDH2 | 3-hydroxybutyrate dehydrogenase, type 2 | No results in PubMed | none |
| 161 | C4orf3 | chromosome 4 open reading frame 3 | No results in PubMed | none |
| 162 | MEF2A | myocyte enhancer factor 2A | No results in PubMed | none |
| 163 | OR7A5 | olfactory receptor, family 7, subfamily A, member 5 | No results in PubMed | none |
| 164 | TFAM | transcription factor A, mitochondrial | 2 [41, 42] | The Down syndrome fetuses were found to have lower TFAM expression than the normal foetuses [41]. |
|  |  |  |  | The fetal developing tissues might differ in the control of mitochondrial biogenesis depending on their energy demand and the age of gestation [42]. |
| 165 | NCAM2 | neural cell adhesion molecule 2 | No results in PubMed | none |
| 166 | CALU | calumenin | 2 [43, 44] | Identification of novel proteins inclusive of CALU that are regulated by peroxynitrite in response to plasma from women with preeclampsia [43]. |
|  |  |  |  | Alteration in the expression of CALU protein in placentas from unexplained recurrent pregnancy loss was observed [44]. |
| 167 | SLC7A11 | solute carrier family 7 (anionic amino acid transporter light chain, xc- system), member 11 | No results in PubMed | none |
| 168 | LUC7L3 | LUC7-like 3 (S. cerevisiae) | No results in PubMed | none |
| 169 | LYSMD3 | LysM, putative peptidoglycan-binding, domain containing 3 | No results in PubMed | none |
| 170 | DCBLD2 | discoidin, CUB and LCCL domain containing 2 | No results in PubMed | none |
| 171 | TMEM123 | transmembrane protein 123 | No results in PubMed | none |
| 172 | SNX18 | sorting nexin 18 | No results in PubMed | none |
| 173 | CMAS | cytidine monophosphate N-acetylneuraminic acid synthetase | No results in PubMed | none |
| 174 | BNIP3L | BCL2/adenovirus E1B 19kDa interacting protein 3-like | No results in PubMed | none |
| 175 | DAB2 | disabled homolog 2, mitogen-responsive phosphoprotein (Drosophila) | No results in PubMed | none |
| 176 | SIN3B | SIN3 homolog B, transcription regulator (yeast) | No results in PubMed | none |
| 177 | RTCD1 | RNA terminal phosphate cyclase domain 1 | No results in PubMed | none |
| 178 | OCLN | occludin | 26 [selection 45, 46] | There was no difference in mRNA expression for occludin in human umbilical vein endothelial cells between normal endothelial cells and those from preeclamptic pregnancies [45]. |
|  |  |  |  | Disorganized tight junction proteins and an altered distribution of occludin in monolayers of endothelial cells cocultured with preeclamptic trophoblast cells were observed [46]. |
| 179 | TYW5 | tRNA-yW synthesizing protein 5 | No results in PubMed | none |
| 180 | CCNB1 | cyclin B1 | 8 [selection 47] | Cyclin B1 was immunolocalized in tissue sections of first trimester pregnancies (weeks 6 and 9-12). Villous cytotrophoblasts were only few stained with anti-cyclinB1. Cyclin B1 was immunolocalized in proximal and distal extravillous cytotrophoblasts of anchoring villi and in EVT which had invaded the upper decidual segments [47]. |
| 181 | C16orf52 | chromosome 16 open reading frame 52 | No results in PubMed | none |
| 182 | C5orf24 | chromosome 5 open reading frame 24 | No results in PubMed | none |
| 183 | KHDRBS1 | KH domain containing, RNA binding, signal transduction associated 1 | No results in PubMed | none |
| 184 | EDNRA | endothelin receptor type A | 5 [selection 48-50] | This gene encodes the receptor for endothelin-1 (ET vasocons-1), a potent trictor peptide produced by vascular endothelial cells. ET-1 appears to play a role in the pathogenesis of some forms of hypertension. ET-1 has been thought to play an important role in the maintenance of blood pressure and some role in the response to injury of endothelial cells. In preeclamptic plasma, an increased concentration of ET-1 has been reported [48, 49]. |
|  |  |  |  | sFLT-1 suppresses the VEGF-induced ET-1 expression [50]. |
| 185 | GORASP1 | golgi reassembly stacking protein 1, 65kDa | No results in PubMed | none |
| 186 | ATXN1 | ataxin 1 | No results in PubMed | none |
| 187 | RNF19A | ring finger protein 19A | No results in PubMed | none |
| 188 | PTAR1 | protein prenyltransferase alpha subunit repeat containing 1 | No results in PubMed | none |
| 189 | RAB11B | RAB11B, member RAS oncogene family | No results in PubMed | none |
| 190 | TMEM135 | transmembrane protein 135 | No results in PubMed | none |
| 191 | RIT1 | Ras-like without CAAX 1 | No results in PubMed | none |
| 192 | NCAM1 | neural cell adhesion molecule 1 | No results in PubMed | none |
| 193 | SLC41A1 | solute carrier family 41, member 1 | 1 [51] | SLC41A1 is the only magnesium responsive gene significantly overexpressed in placentas of preeclamptic women [51]. |
| 194 | F3 | coagulation factor III (thromboplastin, tissue factor) | 1024 [selection 52-55] | There was no difference in tissue factor plasma levels among severe preeclampsia women, normotensive pregnant women and normotensive non-pregnant women [52]. |
|  |  |  |  | The plasma tissue factor level in the pre-eclampsia group was significantly higher than the control group. Higher expression and/or release of tissue factor from the placenta may contribute towards a pathological hypercoagulable state in pre-eclampsia patients [53]. |
|  |  |  |  | The Doppler velocimetry abnormalities were significantly associated with TF expression, which was markedly increased, exclusively, in the endothelial cells within the basal decidua of preeclamptic women [54]. |
|  |  |  |  | Maternal plasma concentrations of tissue factor in patients with pre-eclampsia, but not in those who delivered an SGA neonate, were higher than in women with normal pregnancies [55]. |
| 195 | HIPK2 | homeodomain interacting protein kinase 2 | No results in PubMed | none |
| 196 | ATL1 | atlastin GTPase 1 | No results in PubMed | none |
| 197 | GFER | growth factor, augmenter of liver regeneration | No results in PubMed | none |
| 198 | COPS8 | COP9 constitutive photomorphogenic homolog subunit 8 (Arabidopsis) | No results in PubMed | none |
| 199 | PCDH7 | protocadherin 7 | No results in PubMed | none |
| 200 | ETV1 | ets variant 1 | No results in PubMed | none |
| 201 | SFMBT1 | Scm-like with four mbt domains 1 | No results in PubMed | none |
| 202 | MBNL1 | muscleblind-like (Drosophila) | No results in PubMed | none |
| 203 | TOP2A | topoisomerase (DNA) II alpha 170kDa | No results in PubMed | none |
| 204 | ENC1 | ectodermal-neural cortex 1 (with BTB-like domain) | No results in PubMed | none |
| 205 | KLHL32 | kelch-like 32 (Drosophila) | No results in PubMed | none |
| 206 | PMAIP1 | phorbol-12-myristate-13-acetate-induced protein 1 | No results in PubMed | none |
| 207 | VCL | vinculin | No results in PubMed | none |
| 208 | CNTN3 | contactin 3 (plasmacytoma associated) | No results in PubMed | none |
| 209 | CNR1 | cannabinoid receptor 1 (brain) | 2 [56, 57] | CB1 expression is attenuated in Fallopian tube and decidua of women with ectopic pregnancy [56]. |
|  |  |  |  | The relationship between SNPs in the CNR1 gene and pre-eclampsia risk was identified [57]. |
| 210 | IRX6 | iroquois homeobox 6 | No results in PubMed | none |
| 211 | SMAD2 | SMAD family member 2 | No results in PubMed | none |
| 212 | DPY19L2 | dpy-19-like 2 (C. elegans) | No results in PubMed | none |
| 213 | ITGAV | integrin, alpha V (vitronectin receptor, alpha polypeptide, antigen CD51) | 4 [selection 58] | Distribution pattern of alphaV integrin was detected in cytotrophoblasts and Hofbauer cells in normal and pre-eclamptic placentas. Immunostaining of alphaV integrin was slightly decreased in preeclamptic samples. Decreased immunostaining of integrins in the cytotrophoblasts may consider being a structural basis for decreased placental perfusion in pre-eclampsia [58]. |
| 214 | PAPOLG | poly(A) polymerase gamma | No results in PubMed | none |
| 215 | QRSL1 | glutaminyl-tRNA synthase (glutamine-hydrolyzing)-like 1 | No results in PubMed | none |
| 216 | SDC2 | syndecan 2 | 2 [59, 60] | Syndecan-2 is mostly localized in intracellular compartments, in the extravillous cytotrophoblastic cells and in some fibroblasts of the chorionic plate as well as in the amniotic epithelial cells [59]. |
|  |  |  |  | Syndecan 2 was significantly decreased in FGR placenta samples compared with controls [60]. |
| 217 | PLEKHA1 | pleckstrin homology domain containing, family A (phosphoinositide binding specific) member 1 | No results in PubMed | none |
| 218 | EIF2S1 | eukaryotic translation initiation factor 2, subunit 1 alpha, 35kDa | No results in PubMed | none |
| 219 | POU4F2 | POU class 4 homeobox 2 | No results in PubMed | none |
| 220 | MSL1 | male-specific lethal 1 homolog (Drosophila) | No results in PubMed | none |
| 221 | E2F1 | E2F transcription factor 1 | 4 [61-64] | E2F1 is differentially expressed in peripheral blood mononuclear cells in women with preeclampsia compared to normal controls with false discovery rate (FDR) controlled at 10% [61]. |
|  |  |  |  | E2F1 is down-regulated in the preeclamptic placenta [62]. |
|  |  |  |  | The different expression pattern of E2F-1 in partial and complete moles may be useful in distinguishing these two entities [63]. |
|  |  |  |  | Syncytin-1 modulates placental trophoblast cell proliferation by promoting G1/S transition [64]. |
| 222 | FAM176A | family with sequence similarity 176, member A | No results in PubMed | none |
| 223 | GTF2H5 | general transcription factor IIH, polypeptide 5 | No results in PubMed | none |
| 224 | DOK6 | docking protein 6 | No results in PubMed | none |
| 225 | DYM | dymeclin | No results in PubMed | none |
| 226 | ANKRD28 | ankyrin repeat domain 28 | No results in PubMed | none |
| 227 | CEP57 | centrosomal protein 57kDa | No results in PubMed | none |
| 228 | CELSR3 | cadherin, EGF LAG seven-pass G-type receptor 3 (flamingo homolog, Drosophila) | No results in PubMed | none |
| 229 | SMAD3 | SMAD family member 3 | No results in PubMed | none |
| 230 | CPEB3 | cytoplasmic polyadenylation element binding protein 3 | No results in PubMed | none |
| 231 | BAI2 | brain-specific angiogenesis inhibitor 2 | No results in PubMed | none |
| 232 | SASS6 | spindle assembly 6 homolog (C. elegans) | No results in PubMed | none |
| 233 | TET3 | tet methylcytosine dioxygenase 3 | No results in PubMed | none |
| 234 | MSH3 | mutS homolog 3 (E. coli) | No results in PubMed | none |
| 235 | ABHD5 | abhydrolase domain containing 5 | No results in PubMed | none |
| 236 | RRN3 | RRN3 RNA polymerase I transcription factor homolog (S. cerevisiae) | No results in PubMed | none |
| 237 | KIF26B | kinesin family member 26B | No results in PubMed | none |
| 238 | DCUN1D3 | DCN1, defective in cullin neddylation 1, domain containing 3 (S. cerevisiae) | No results in PubMed | none |
| 239 | ITFG1 | integrin alpha FG-GAP repeat containing 1 | No results in PubMed | none |
| 240 | ST8SIA1 | ST8 alpha-N-acetyl-neuraminide alpha-2,8-sialyltransferase 1 | No results in PubMed | none |
| 241 | DCUN1D5 | DCN1, defective in cullin neddylation 1, domain containing 5 (S. cerevisiae) | No results in PubMed | none |
| 242 | PIKFYVE | phosphoinositide kinase, FYVE finger containing | No results in PubMed | none |
| 243 | PIBF1 | progesterone immunomodulatory binding factor 1 | No results in PubMed | none |
| 244 | NDFIP1 | Nedd4 family interacting protein 1 | No results in PubMed | none |
| 245 | AGGF1 | angiogenic factor with G patch and FHA domains 1 | No results in PubMed | none |
| 246 | IYD | iodotyrosine deiodinase | No results in PubMed | none |
| 247 | ABL2 | v-abl Abelson murine leukemia viral oncogene homolog 2 | No results in PubMed | none |
| 248 | MYO3A | myosin IIIA | No results in PubMed | none |
| 249 | CTSS | cathepsin S | No results in PubMed | none |
| 250 | RHOV | ras homolog gene family, member V | No results in PubMed | none |
| 251 | GTF2IRD2B | GTF2I repeat domain containing 2B | No results in PubMed | none |
| 252 | GNAI3 | guanine nucleotide binding protein (G protein), alpha inhibiting activity polypeptide 3 | No results in PubMed | none |
| 253 | FGF18 | fibroblast growth factor 18 | No results in PubMed | none |
| 254 | SLC35A5 | solute carrier family 35, member A5 | No results in PubMed | none |
| 255 | GLRX | glutaredoxin (thioltransferase) | 7 [selection 65-67] | The microarray data highlighted non-imprinted gene acting in oxidative metabolism (GLRX) as differentially expressed in IUGR vs. non-IUGR placentae [65]. |
|  |  |  |  | Glutaredoxin reducing systems are affected in placenta from pregnancies with preeclampsia and/or growth restriction of fetuses, and that the decrease correlates to the severity of the condition [66]. |
|  |  |  |  | The preeclamptic placentae are exposed to oxidative stress and the protein thiol/disulphide oxidoreductases are adaptively induced in preeclamptic placentae, suggesting possible roles for thioredoxin, glutaredoxin, and protein disulphide isomerase in protecting placental functions against oxidative stress caused by pre-eclampsia [67]. |
| 256 | THAP6 | THAP domain containing 6 | No results in PubMed | none |
| 257 | GIMAP4 | GTPase, IMAP family member 4 | No results in PubMed | none |
| 258 | ZNF140 | zinc finger protein 140 | No results in PubMed | none |
| 259 | ATP6V1C1 | ATPase, H+ transporting, lysosomal 42kDa, V1 subunit C1 | No results in PubMed | none |
| 260 | CHIC1 | cysteine-rich hydrophobic domain 1 | No results in PubMed | none |
| 261 | EFCAB2 | EF-hand calcium binding domain 2 | No results in PubMed | none |
| 262 | RBM41 | RNA binding motif protein 41 | No results in PubMed | none |
| 263 | GTF2IRD2 | GTF2I repeat domain containing 2 | No results in PubMed | none |
| 264 | NAALADL2 | N-acetylated alpha-linked acidic dipeptidase-like 2 | No results in PubMed | none |
| 265 | ZNF99 | zinc finger protein 99 | No results in PubMed | none |
| 266 | RASGRP1 | RAS guanyl releasing protein 1 (calcium and DAG-regulated) | No results in PubMed | none |
| 267 | INSIG2 | insulin induced gene 2 | No results in PubMed | none |
| 268 | CGA | glycoprotein hormones, alpha polypeptide | 48 [selection 68] | The four human glycoprotein hormones chorionic gonadotropin (CG), luteinizing hormone (LH), follicle stimulating hormone (FSH), and thyroid stimulating hormone (TSH) are dimers consisting of alpha and beta subunits that are associated noncovalently. The alpha subunits of these hormones are identical; however, their beta chains are unique and confer biological specificity [68]. |
| 269 | RGS2 | regulator of G-protein signaling 2, 24kDa | 9 [selection 69-70] | Using intra-patient analysis, four candidate genes, phosphoglycerate kinase 1 (PGK1), regulator of G-protein signalling 2 (RGS2), regulator of G-protein signalling 3 (RGS3) and cell division cycle 42 (CDC42) showed a difference between follicular cells from follicles leading to a pregnancy or developmental failure [69]. |
|  |  |  |  | RGS2 rs4606 SNP may affect the risk and progression of preeclampsia [70]. |
| 270 | CDS1 | CDP-diacylglycerol synthase (phosphatidate cytidylyltransferase) 1 | No results in PubMed | none |
| 271 | SMAD7 | SMAD family member 7 | No results in PubMed | none |
| 272 | GNL3L | guanine nucleotide binding protein-like 3 (nucleolar)-like | No results in PubMed | none |
| 273 | ADAMTS5 | ADAM metallopeptidase with thrombospondin type 1 motif, 5 | 2 [71, 72] | ADAMTS5 expression is restricted to decidualized stromal cells of the human endometrium in vivo and is subject to regulation by cytokines in vitro [71]. |
|  |  |  |  | The restricted expression pattern of ADAMTS4 and ADAMTS5 and their increased expression in gestational trophoblastic diseases suggest that these 2 ADAMTS subtypes are associated with a biological phenotype of trophoblasts involved in human placentation and the development of gestational trophoblastic diseases [72]. |
| 274 | CNOT6L | CCR4-NOT transcription complex, subunit 6-like | No results in PubMed | none |
| 275 | NPAS2 | neuronal PAS domain protein 2 | No results in PubMed | none |
| 276 | LRRC10 | leucine rich repeat containing 10 | No results in PubMed | none |
| 277 | CYP4F2 | cytochrome P450, family 4, subfamily F, polypeptide 2 | No results in PubMed | none |
| 278 | SEC24A | SEC24 family, member A (S. cerevisiae) | No results in PubMed | none |
| 279 | KCNE4 | potassium voltage-gated channel, Isk-related family, member 4 | No results in PubMed | none |
| 280 | ZNF697 | zinc finger protein 697 | No results in PubMed | none |
| 281 | PRIM2 | primase, DNA, polypeptide 2 (58kDa) | 1 [73] | Lack of genomic imprinting of DNA primase, polypeptide 2 (PRIM2) in human term placenta and white blood cells was described [73]. |
| 282 | TLN2 | talin 2 | No results in PubMed | none |
| 283 | FXN | frataxin | No results in PubMed | none |
| 284 | CHD2 | chromodomain helicase DNA binding protein 2 | No results in PubMed | none |
| 285 | CCDC83 | coiled-coil domain containing 83 | No results in PubMed | none |
| 286 | ZNF793 | zinc finger protein 793 | No results in PubMed | none |
| 287 | UGP2 | UDP-glucose pyrophosphorylase 2 | No results in PubMed | none |
| 288 | TTYH2 | tweety homolog 2 (Drosophila) | No results in PubMed | none |
| 289 | LOC100506972 | uncharacterized protein C14orf113-like | No results in PubMed | none |
| 290 | CDKN1A | cyclin-dependent kinase inhibitor 1A (p21, Cip1) | 60 [selection 74, 75] | Leukocytes are activated in preeclampsia, they showed up-regulation of Nuclear Factor of Kappa light chain gene enhancer in B cells inhibitor (NFkappaB-1A) and cyclin-dependent kinase inhibitor CDKN1A compared with normotensive pregnant women [74]. |
|  |  |  |  | The p53-CDKN1A and p53-Bax signaling pathways appear to be activated in recurrent spontaneous abortion [75]. |
| 291 | GRM5 | glutamate receptor, metabotropic 5 | No results in PubMed | none |
| 292 | PRKAA2 | protein kinase, AMP-activated, alpha 2 catalytic subunit | No results in PubMed | none |
| 293 | MSL2 | male-specific lethal 2 homolog (Drosophila) | No results in PubMed | none |
| 294 | BACE1 | beta-site APP-cleaving enzyme 1 | No results in PubMed | none |
| 295 | IFNAR1 | interferon (alpha, beta and omega) receptor 1 | No results in PubMed | none |
| 296 | RBM15B | RNA binding motif protein 15B | No results in PubMed | none |
| 297 | UBA6 | ubiquitin-like modifier activating enzyme 6 | No results in PubMed | none |
| 298 | ITPKB | inositol-trisphosphate 3-kinase B | No results in PubMed | none |
| 299 | G2E3 | G2/M-phase specific E3 ubiquitin protein ligase | No results in PubMed | none |
| 300 | RIMKLA | ribosomal modification protein rimK-like family member A | No results in PubMed | none |
| 301 | VPS26A | vacuolar protein sorting 26 homolog A (S. pombe) | No results in PubMed | none |
| 302 | PCK1 | phosphoenolpyruvate carboxykinase 1 (soluble) | 2 [76, 77] | Demonstration that the expression of BCL2L11, PCK1 and NFIB in cumulus cells significantly correlated with embryo potential and successful pregnancy [76]. |
|  |  |  |  | Genes in cumulus samples resulting in a successful pregnancy were predominantly up-regulated, including BCL2L11 and PCK1, respectively involved in apoptosis and gluconeogenesis [77]. |
| 303 | TAF5L | TAF5-like RNA polymerase II, p300/CBP-associated factor (PCAF)-associated factor, 65kDa | No results in PubMed | none |
| 304 | PHACTR4 | phosphatase and actin regulator 4 | No results in PubMed | none |
| 305 | FAM36A | family with sequence similarity 36, member A | No results in PubMed | none |
| 306 | PEX11B | peroxisomal biogenesis factor 11 beta | No results in PubMed | none |
| 307 | CHM | choroideremia (Rab escort protein 1) | No results in PubMed | none |
| 308 | POLR3F | polymerase (RNA) III (DNA directed) polypeptide F, 39 kDa | No results in PubMed | none |
| 309 | CBX5 | chromobox homolog 5 | No results in PubMed | none |
| 310 | DCTN5 | dynactin 5 (p25) | No results in PubMed | none |
| 311 | GPM6B | glycoprotein M6B | No results in PubMed | none |
| 312 | ANGEL2 | angel homolog 2 (Drosophila) | No results in PubMed | none |
| 313 | HOGA1 | 4-hydroxy-2-oxoglutarate aldolase 1 | No results in PubMed | none |
| 314 | ZMAT3 | zinc finger, matrin-type 3 | No results in PubMed | none |
| 315 | TGFB2 | transforming growth factor, beta 2 | 67 [selection 78-80] | Measurement of maternal serum TGF-beta2 levels in preeclampsia may be a useful biomarker for the assessment of the severity of disease and fetal outcome in preeclampsia [78]. |
|  |  |  |  | TGF-beta2 was present in extravillous trophoblast. No changes in expression of either isoform were found in placenta or placental bed in PE or FGR compared with normal pregnancy [79]. |
|  |  |  |  | Extravillous trophoblast within the placental bed expressed TGF-beta2 but not TGF-beta1 or TGF-beta3 while extracellular TGF-beta1 and cytoplasmic TGF-beta2 were detected in decidua. These data suggest that TGF-beta1 and TGF-beta2 but not TGF-beta3 may play a role in trophoblast invasion [80]. |
| 316 | PPP2R5C | protein phosphatase 2, regulatory subunit B', gamma | No results in PubMed | none |
| 317 | ANLN | anillin, actin binding protein | No results in PubMed | none |
| 318 | C9orf5 | chromosome 9 open reading frame 5 | No results in PubMed | none |
| 319 | SEC14L2 | SEC14-like 2 (S. cerevisiae) | No results in PubMed | none |
| 320 | MBTPS2 | membrane-bound transcription factor peptidase, site 2 | No results in PubMed | none |
| 321 | ABRA | actin-binding Rho activating protein | No results in PubMed | none |
| 322 | CLEC2D | C-type lectin domain family 2, member D | No results in PubMed | none |
| 323 | HS6ST3 | heparan sulfate 6-O-sulfotransferase 3 | No results in PubMed | none |
| 324 | KLHL2 | kelch-like 2, Mayven (Drosophila) | No results in PubMed | none |
| 325 | SHE | Src homology 2 domain containing E | No results in PubMed | none |
| 326 | LARP4B | La ribonucleoprotein domain family, member 4B | No results in PubMed | none |
| 327 | PUM2 | pumilio homolog 2 (Drosophila) | No results in PubMed | none |
| 328 | LOC100653112 | heparan-sulfate 6-O-sulfotransferase 3-like | No results in PubMed | none |
| 329 | FBXL3 | F-box and leucine-rich repeat protein 3 | No results in PubMed | none |
| 330 | LIN28B | lin-28 homolog B (C. elegans) | 4 [selection 81] | The potential involvement of LIN28B/Let-7 (de)regulated pathways in the pathophysiology of ectopic pregnancy in humans was observed [81]. |
| 331 | PANX1 | pannexin 1 | No results in PubMed | none |
| 332 | VPS13D | vacuolar protein sorting 13 homolog D (S. cerevisiae) | No results in PubMed | none |
| 333 | SART3 | squamous cell carcinoma antigen recognized by T cells 3 | No results in PubMed | none |
| 334 | GMCL1 | germ cell-less homolog 1 (Drosophila) | No results in PubMed | none |
| 335 | ABCG2 | ATP-binding cassette, sub-family G (WHITE), member 2 | No results in PubMed | none |
| 336 | BRD1 | bromodomain containing 1 | No results in PubMed | none |
| 337 | TMPO | thymopoietin | No results in PubMed | none |
| 338 | CNOT6 | CCR4-NOT transcription complex, subunit 6 | No results in PubMed | none |
| 339 | ABCA1 | ATP-binding cassette, sub-family A (ABC1), member 1 | 27 [selection 82, 83] | LXRA together with ABCA1 are specifically expressed in the human placenta and can be regulated by hypoxia. Deregulation of this system in early preeclampsia might be the result of placental hypoxia and hence might have consequences for maternal-fetal cholesterol transport [82]. |
|  |  |  |  | In gestational disease, there is a specific down-regulation of placental ABCA1 expression at sites of feto-maternal lipid exchange in preeclampsia. At a functional level, the increase in placental lipid concentrations provides indirect evidence of an impaired transport capacity of ABCA1 in this disease [83]. |
| 340 | ABCA5 | ATP-binding cassette, sub-family A (ABC1), member 5 | No results in PubMed | none |
| 341 | UNG | uracil-DNA glycosylase | 17 [selection 84, 85] | The NH2-terminal amino acid sequence of uracil-DNA glycosylase from human placenta was purification and determined [84]. |
|  |  |  |  | Uracil-DNA glycosylase was, surprisingly, approximately 4-fold lower in fetal tissues compared with adult tissues. These findings indicate that several fetal organs may be more sensitive than adult organs to some alkylating agents that are known to occur in the environment [85]. |
| 342 | GRIN3A | glutamate receptor, ionotropic, N-methyl-D-aspartate 3A | No results in PubMed | none |
| 343 | SEPHS1 | selenophosphate synthetase 1 | No results in PubMed | none |
| 344 | NR2C2 | nuclear receptor subfamily 2, group C, member 2 | No results in PubMed | none |
| 345 | MAPK9 | mitogen-activated protein kinase 9 | No results in PubMed | none |
| 346 | C14orf129 | chromosome 14 open reading frame 129 | No results in PubMed | none |
| 347 | DDOST | dolichyl-diphosphooligosaccharide--protein glycosyltransferase | No results in PubMed | none |
| 348 | XPO4 | exportin 4 | No results in PubMed | none |
| 349 | C5orf41 | chromosome 5 open reading frame 41 | No results in PubMed | none |
| 350 | DBX2 | developing brain homeobox 2 | No results in PubMed | none |
| 351 | BCDIN3D | BCDIN3 domain containing | No results in PubMed | none |
| 352 | ZNF670 | zinc finger protein 670 | No results in PubMed | none |
| 353 | MGAT4A | mannosyl (alpha-1,3-)-glycoprotein beta-1,4-N-acetylglucosaminyltransferase, isozyme A | No results in PubMed | none |
| 354 | DSN1 | DSN1, MIND kinetochore complex component, homolog (S. cerevisiae) | No results in PubMed | none |
| 355 | ZC3H12C | zinc finger CCCH-type containing 12C | No results in PubMed | none |
| 356 | ZNF608 | zinc finger protein 608 | No results in PubMed | none |
| 357 | AHRR | aryl-hydrocarbon receptor repressor | 5 [selection 86] | Differential DNA methylation at epigenome-wide statistical significance for 26 CpGs mapped to 10 genes including CpGs in AHRR at in a U.S. birth cohort was identified. AHRR and CYP1A1 play a key role in the aryl hydrocarbon receptor signaling pathway, which mediates the detoxification of the components of tobacco smoke [86]. |
| 358 | SHQ1 | SHQ1 homolog (S. cerevisiae) | No results in PubMed | none |
| 359 | DSEL | dermatan sulfate epimerase-like | No results in PubMed | none |
| 360 | GLCE | glucuronic acid epimerase | No results in PubMed | none |
| 361 | ZBTB1 | zinc finger and BTB domain containing 1 | No results in PubMed | none |
| 362 | MFSD9 | major facilitator superfamily domain containing 9 | No results in PubMed | none |
| 363 | BTAF1 | BTAF1 RNA polymerase II, B-TFIID transcription factor-associated, 170kDa (Mot1 homolog, S. cerevisiae) | No results in PubMed | none |
| 364 | DDX6 | DEAD (Asp-Glu-Ala-Asp) box polypeptide 6 | No results in PubMed | none |
| 365 | GPCPD1 | glycerophosphocholine phosphodiesterase GDE1 homolog (S. cerevisiae) | No results in PubMed | none |
| 366 | GOLGA1 | golgin A1 | No results in PubMed | none |
| 367 | CDKN2B | cyclin-dependent kinase inhibitor 2B (p15, inhibits CDK4) | 2 [87, 88] | Several risk alleles of type 2 diabetes were associated with gestational diabetes mellitus in pregnant Chinese women [87]. |
|  |  |  |  | Some of the type 2 diabetes-associated genetic variants that were discovered in the recent GWA studies are also associated with gestational diabetes mellitus in Koreans [88]. |
| 368 | ELL2 | elongation factor, RNA polymerase II, 2 | No results in PubMed | none |
| 369 | CDC42BPB | CDC42 binding protein kinase beta (DMPK-like) | No results in PubMed | none |
| 370 | USP18 | ubiquitin specific peptidase 18 | No results in PubMed | none |
| 371 | TBL1X | transducin (beta)-like 1X-linked | No results in PubMed | none |
| 372 | ITPRIPL2 | inositol 1,4,5-trisphosphate receptor interacting protein-like 2 | No results in PubMed | none |
| 373 | ADAMTSL3 | ADAMTS-like 3 | No results in PubMed | none |
| 374 | MARCKS | myristoylated alanine-rich protein kinase C substrate | No results in PubMed | none |
| 375 | C8orf37 | chromosome 8 open reading frame 37 | No results in PubMed | none |
| 376 | LINGO1 | leucine rich repeat and Ig domain containing 1 | No results in PubMed | none |
| 377 | ICK | intestinal cell (MAK-like) kinase | No results in PubMed | none |
| 378 | FBXO39 | F-box protein 39 | No results in PubMed | none |
| 379 | POU3F2 | POU class 3 homeobox 2 | No results in PubMed | none |
| 380 | SHANK2 | SH3 and multiple ankyrin repeat domains 2 | No results in PubMed | none |
| 381 | PCDHAC2 | protocadherin alpha subfamily C, 2 | No results in PubMed | none |
| 382 | ZNF564 | zinc finger protein 564 | No results in PubMed | none |
| 383 | FAM22G | family with sequence similarity 22, member G | No results in PubMed | none |
| 384 | SSFA2 | sperm specific antigen 2 | No results in PubMed | none |
| 385 | ABCC9 | ATP-binding cassette, sub-family C (CFTR/MRP), member 9 | No results in PubMed | none |
| 386 | MFF | mitochondrial fission factor | No results in PubMed | none |
| 387 | KLHL15 | kelch-like 15 (Drosophila) | No results in PubMed | none |
| 388 | FBXL2 | F-box and leucine-rich repeat protein 2 | No results in PubMed | none |
| 389 | SLK | STE20-like kinase | 1 [89] | Mammalian Ste20-like protein kinase 3 mediates trophoblast apoptosis in spontaneous delivery [89]. |
| 390 | RHOA | ras homolog gene family, member A | No results in PubMed | none |
| 391 | FAM178A | family with sequence similarity 178, member A | No results in PubMed | none |
| 392 | PCNP | PEST proteolytic signal containing nuclear protein | No results in PubMed | none |
| 393 | SYT1 | synaptotagmin I | No results in PubMed | none |
| 394 | UTP14C | UTP14, U3 small nucleolar ribonucleoprotein, homolog C (yeast) | No results in PubMed | none |
| 395 | CLSPN | claspin | No results in PubMed | none |
| 396 | OLR1 | oxidized low density lipoprotein (lectin-like) receptor 1 | 23 [selection 90, 91] | The LOX-1 protein expression levels in the placenta tissues of the early-onset preeclampsia were significantly higher than those of the normal pregnant women [90]. |
|  |  |  |  | Increased levels of oxLDL in plasma may induce excess expression of LOX-1 in placenta, which may be involved in the pathophysiological processes of pre-eclampsia [91]. |
| 397 | INPP5F | inositol polyphosphate-5-phosphatase F | No results in PubMed | none |
| 398 | NOD2 | nucleotide-binding oligomerization domain containing 2 | 11 [selection 92-94] | An association of common TLR4 and NOD2 gene variants, and pro-inflammatory phenotype with a history of early-onset preeclampsia and HELLP syndrome was presented. These findings suggest involvement of the maternal innate immune system in severe hypertensive disorders of pregnancy [92]. |
|  |  |  |  | NOD1 and NOD2 are increased in labouring fetal membranes and myometrium and with bacterial infection [93]. |
|  |  |  |  | The overall influence of the investigated polymorphisms in NOD2 on the development of preterm delivery seems moderate [94]. |
| 399 | OSBPL3 | oxysterol binding protein-like 3 | No results in PubMed | none |
| 400 | KRT6C | keratin 6C | No results in PubMed | none |
| 401 | APBB2 | amyloid beta (A4) precursor protein-binding, family B, member 2 | No results in PubMed | none |
| 402 | PKHD1 | polycystic kidney and hepatic disease 1 (autosomal recessive) | 138 [selection 95] | Autosomal recessive polycystic kidney disease (ARPKD), caused by the PKHD1 gene mutations, is one of the most common hereditary nephropathies in childhood. Prenatal diagnosis of autosomal recessive polycystic kidney disease by molecular genetic analysis is available [95]. |
| 403 | TRIM13 | tripartite motif containing 13 | No results in PubMed | none |
| 404 | LRP8 | low density lipoprotein receptor-related protein 8, apolipoprotein e receptor | 1 [96] | Polymorphism in maternal LRP8 gene is associated with fetal growth [96]. |
| 405 | RSL24D1 | ribosomal L24 domain containing 1 | No results in PubMed | none |
| 406 | QKI | QKI, KH domain containing, RNA binding | No results in PubMed | none |
| 407 | MBNL3 | muscleblind-like 3 (Drosophila) | No results in PubMed | none |
| 408 | NPAS3 | neuronal PAS domain protein 3 | No results in PubMed | none |
| 409 | S1PR3 | sphingosine-1-phosphate receptor 3 | 4 [selection 97-99] | S1P-induced vasoconstriction in human placental arteries is mediated by increased Ca(2+)-sensitization through activation of Rho-associated kinases, and this vasoconstriction also is modulated by nitric oxide. Identification of these actions of S1P in the placental vasculature is important for understanding both normal and potentially abnormal vascular adaptations with pregnancy [97, 98]. |
|  |  |  |  | S1PR3 receptors are highly expressed in human endometrial stromal cells [99]. |
| 410 | ACP1 | acid phosphatase 1, soluble | 26 [selection 100, 101] | When the mother to fetus ACP1 S isoform concentration ratio is in favour of the mother, the probability of survival of the fetus is greater than in the opposite situation [100]. |
|  |  |  |  | Women with high-ACP1 activity show the lowest susceptibility to repeated spontaneous abortion [101]. |
| 411 | RIMBP2 | RIMS binding protein 2 | No results in PubMed | none |
| 412 | ZNF84 | zinc finger protein 84 | No results in PubMed | none |
| 413 | CCDC75 | coiled-coil domain containing 75 | No results in PubMed | none |
| 414 | KIAA1324 | KIAA1324 | No results in PubMed | none |
| 415 | PLOD2 | procollagen-lysine, 2-oxoglutarate 5-dioxygenase 2 | No results in PubMed | none |
| 416 | EPDR1 | ependymin related protein 1 (zebrafish) | No results in PubMed | none |
| 417 | COLEC10 | collectin sub-family member 10 (C-type lectin) | No results in PubMed | none |
| 418 | UBE2W | ubiquitin-conjugating enzyme E2W (putative) | No results in PubMed | none |
| 419 | BCOR | BCL6 corepressor | 1 [102] | Prenatal diagnosis of X-linked recessive Lenz microphthalmia syndrome caused by BCOR mutations is available [102]. |
| 420 | SIGLEC11 | sialic acid binding Ig-like lectin 11 | No results in PubMed | none |
| 421 | RAB39B | RAB39B, member RAS oncogene family | No results in PubMed | none |
| 422 | PKN2 | protein kinase N2 | No results in PubMed | none |
| 423 | C19orf42 | chromosome 19 open reading frame 42 | No results in PubMed | none |
| 424 | KLF10 | Kruppel-like factor 10 | No results in PubMed | none |
| 425 | ACBD5 | acyl-CoA binding domain containing 5 | No results in PubMed | none |
| 426 | LBR | lamin B receptor | No results in PubMed | none |
| 427 | C1orf212 | chromosome 1 open reading frame 212 | No results in PubMed | none |
| 428 | PPP2R3A | protein phosphatase 2, regulatory subunit B'', alpha | No results in PubMed | none |
| 429 | ENTPD4 | ectonucleoside triphosphate diphosphohydrolase 4 | No results in PubMed | none |
| 430 | RBPMS | RNA binding protein with multiple splicing | No results in PubMed | none |
| 431 | FAM177A1 | family with sequence similarity 177, member A1 | No results in PubMed | none |
| 432 | PAFAH1B2 | platelet-activating factor acetylhydrolase 1b, catalytic subunit 2 (30kDa) | 40 [selection 103, 104] | This gene encodes the beta subunit of PAFAH; the other subunits are alpha and gamma. Platelet-activating factor acetylhydrolase (PAF-AH) inactivates platelet-activating factor (PAF) into acetate and LYSO-PAF. Increased PAF-AH activity in trophoblasts in the preeclamptic placenta was detected [103]. |
|  |  |  |  | The neonates of women with severe PE had significantly higher plasma PAF-AH, L-PAF-AH activities, and ratio of L-PAF-AH to H-PAF-AH activities than the neonates of women with normal pregnancies [104]. |
| 433 | PTK2 | PTK2 protein tyrosine kinase 2 | No results in PubMed | none |
| 434 | OSBPL10 | oxysterol binding protein-like 10 | No results in PubMed | none |
| 435 | IRS1 | insulin receptor substrate 1 | 35 [selection 105-109] | This gene encodes a protein which is phosphorylated by insulin receptor tyrosine kinase. Type 1 or type 2 diabetes is risk factors for preeclampsia [105-107]. |
|  |  |  |  | Insulin resistance in human preeclamptic placenta is mediated by serine phosphorylation of insulin receptor substrate-1 and -2 [108]. Mutations in this gene are associated with type II diabetes and susceptibility to insulin resistance [109]. |
| 436 | ADAM22 | ADAM metallopeptidase domain 22 | No results in PubMed | none |
| 437 | TRIM22 | tripartite motif containing 22 | No results in PubMed | none |
| 438 | DBT | dihydrolipoamide branched chain transacylase E2 | No results in PubMed | none |
| 439 | OCRL | oculocerebrorenal syndrome of Lowe | No results in PubMed | none |
| 440 | DCC | deleted in colorectal carcinoma | No results in PubMed | none |
| 441 | CEP164 | centrosomal protein 164kDa | No results in PubMed | none |
| 442 | ZFP91 | zinc finger protein 91 homolog (mouse) | No results in PubMed | none |
| 443 | SRGAP2 | SLIT-ROBO Rho GTPase activating protein 2 | No results in PubMed | none |
| 444 | TMOD2 | tropomodulin 2 (neuronal) | No results in PubMed | none |
| 445 | GRIA3 | glutamate receptor, ionotrophic, AMPA 3 | No results in PubMed | none |
| 446 | FBXO33 | F-box protein 33 | No results in PubMed | none |
| 447 | PTGFRN | prostaglandin F2 receptor negative regulator | No results in PubMed | none |
| 448 | PSD3 | pleckstrin and Sec7 domain containing 3 | No results in PubMed | none |
| 449 | FAM20B | family with sequence similarity 20, member B | No results in PubMed | none |
| 450 | TM9SF3 | transmembrane 9 superfamily member 3 | No results in PubMed | none |
| 451 | ARMC1 | armadillo repeat containing 1 | No results in PubMed | none |
| 452 | THRA | thyroid hormone receptor, alpha | No results in PubMed | none |
| 453 | GOSR1 | golgi SNAP receptor complex member 1 | No results in PubMed | none |
| 454 | TOMM22 | translocase of outer mitochondrial membrane 22 homolog (yeast) | No results in PubMed | none |
| 455 | PAH | phenylalanine hydroxylase | 67 [selection 110] | Phenylketonuria is the most prevalent disorder caused by an inborn error in aminoacid metabolism. It results from mutations in the phenylalanine hydroxylase gene [110]. |
| 456 | FIBIN | fin bud initiation factor homolog (zebrafish) | No results in PubMed | none |
| 457 | ZBTB34 | zinc finger and BTB domain containing 34 | No results in PubMed | none |
| 458 | CMPK1 | cytidine monophosphate (UMP-CMP) kinase 1, cytosolic | No results in PubMed | none |
| 459 | ZNF704 | zinc finger protein 704 | No results in PubMed | none |
| 460 | PTPN13 | protein tyrosine phosphatase, non-receptor type 13 (APO-1/CD95 (Fas)-associated phosphatase) | No results in PubMed | none |
| 461 | DGKE | diacylglycerol kinase, epsilon 64kDa | No results in PubMed | none |
| 462 | C7orf23 | chromosome 7 open reading frame 23 | No results in PubMed | none |
| 463 | DIAPH2 | diaphanous homolog 2 (Drosophila) | No results in PubMed | none |
| 464 | DDX27 | DEAD (Asp-Glu-Ala-Asp) box polypeptide 27 | No results in PubMed | none |
| 465 | DENND1B | DENN/MADD domain containing 1B | No results in PubMed | none |
| 466 | FCF1 | FCF1 small subunit (SSU) processome component homolog (S. cerevisiae) | No results in PubMed | none |
| 467 | TBX18 | T-box 18 | No results in PubMed | none |
| 468 | CSMD3 | CUB and Sushi multiple domains 3 | No results in PubMed | none |
| 469 | ZNF365 | zinc finger protein 365 | No results in PubMed | none |
| 470 | ZNF587 | zinc finger protein 587 | No results in PubMed | none |
| 471 | GRIP1 | glutamate receptor interacting protein 1 | 1 [111] | Mutations in GRIP1 cause Fraser syndrome [111]. |
| 472 | C11orf88 | chromosome 11 open reading frame 88 | No results in PubMed | none |
| 473 | SETD4 | SET domain containing 4 | No results in PubMed | none |
| 474 | RAB22A | RAB22A, member RAS oncogene family | No results in PubMed | none |
| 475 | EHD3 | EH-domain containing 3 | No results in PubMed | none |
| 476 | TNN | tenascin N | No results in PubMed | none |
| 477 | DMD | dystrophin | 138 [selection 112] | The mutated DMD gene causes [Duchenne muscular dystrophy](http://en.wikipedia.org/wiki/Duchenne_muscular_dystrophy) and [Becker's muscular dystrophy](http://en.wikipedia.org/wiki/Becker%27s_muscular_dystrophy). Accurate and reliable method for detecting deletional/duplicational mutations of DMD gene as well as for prenatal diagnosis and detection of female carriers is reported [112]. |
| 478 | DMP1 | dentin matrix acidic phosphoprotein 1 | No results in PubMed | none |
| 479 | GPLD1 | glycosylphosphatidylinositol specific phospholipase D1 | 1 [113] | Equivalent amounts of a proteolytically-cleaved 50 kDa GPI-PLD protein is detected in both normal and preeclamptic placentae [113]. |
| 480 | LOC100130357 | uncharacterized LOC100130357 | No results in PubMed | none |
| 481 | NTRK2 | neurotrophic tyrosine kinase, receptor, type 2 | 1 [114] | Differential endothelial expression of NTRK2 in IUGR versus preterm and term placental samples [114]. |
| 482 | ZBTB37 | zinc finger and BTB domain containing 37 | No results in PubMed | none |
| 483 | ZNF200 | zinc finger protein 200 | No results in PubMed | none |
| 484 | FURIN | furin (paired basic amino acid cleaving enzyme) | 21 [selection 115, 116] | The proprotein convertase furin in human trophoblast may play a role in promoting trophoblast cell migration and invasion [115]. |
|  |  |  |  | The proprotein convertase furin is required for trophoblast syncytialization [116]. |
| 485 | LOC100287482 | uncharacterized LOC100287482 | No results in PubMed | none |
| 486 | SEPT8 | septin 8 | No results in PubMed | none |
| 487 | GZF1 | GDNF-inducible zinc finger protein 1 | No results in PubMed | none |
| 488 | ANO6 | anoctamin 6 | No results in PubMed | none |
| 489 | CLEC16A | C-type lectin domain family 16, member A | No results in PubMed | none |
| 490 | ZBTB6 | zinc finger and BTB domain containing 6 | No results in PubMed | none |
| 491 | HOOK3 | hook homolog 3 (Drosophila) | No results in PubMed | none |
| **492** | **ANGPT1** | **angiopoietin 1** | **79 (selection 117, 118)** | **Ang-1/Ang-2 ratio in first trimester sera is associated with most adverse pregnancy outcomes** **(small for gestational age, preterm birth, preeclampsia, miscarriage >10 weeks, and stillbirth), but do not predict outcomes any better than clinical and maternal risk factor information [117].** |
|  |  |  |  | The ANGPT1 rs2507800 polymorphism may have a potential role in screening women to predict the risk of preeclampsia, SGA babies and spontaneous preterm birth [118]. |
| 493 | CCDC108 | coiled-coil domain containing 108 | No results in PubMed | none |
| 494 | ZNF229 | zinc finger protein 229 | No results in PubMed | none |
| 495 | RCAN3 | RCAN family member 3 | No results in PubMed | none |
| 496 | TBX1 | T-box 1 | 14 [selection 119] | TBX1 encodes a DNA binding transcription factor that is commonly deleted in human with DiGeorge syndrome and plays an important role in heart development [119]. |
| 497 | TMEM136 | transmembrane protein 136 | No results in PubMed | none |
| 498 | ZNF621 | zinc finger protein 621 | No results in PubMed | none |
| 499 | IPO5 | importin 5 | No results in PubMed | none |
| 500 | MAGI3 | membrane associated guanylate kinase, WW and PDZ domain containing 3 | No results in PubMed | none |
| 501 | MCM9 | minichromosome maintenance complex component 9 | No results in PubMed | none |
| 502 | KIAA1377 | KIAA1377 | No results in PubMed | none |
| 503 | OPALIN | oligodendrocytic myelin paranodal and inner loop protein | No results in PubMed | none |
| 504 | ADAM18 | ADAM metallopeptidase domain 18 | No results in PubMed | none |
| 505 | HEPH | hephaestin | No results in PubMed | none |
| 506 | PTBP3 | polypyrimidine tract binding protein 3 | No results in PubMed | none |
| 507 | PITPNA | phosphatidylinositol transfer protein, alpha | No results in PubMed | none |
| 508 | PTPN1 | protein tyrosine phosphatase, non-receptor type 1 | 1 [120] | PTPIP51 and PTP1B play a role in differentiation and apoptosis of the cytotrophoblast and syncytiotrophoblast, respectively. Moreover, PTPIP51 may also serve as a cellular signalling partner in angiogenesis and vascular remodelling [120]. |
| 509 | CLEC5A | C-type lectin dom | No results in PubMed | none |

References

1. Lapaire O, Grill S, Lalevee S, Kolla V, Hösli I, Hahn S (2012) Microarray screening for novel preeclampsia biomarker candidates. [Fetal Diagn Ther](http://www.ncbi.nlm.nih.gov/pubmed/22472943) 31: 147-53.
2. Calicchio R, Buffat C, Mathieu JR, Ben Salem N, Mehats C, et al. (2013) Preeclamptic plasma induces transcription modifications involving the AP-1 transcriptional regulator JDP2 in endothelial cells. Am J Pathol 183: 1993-2006.
3. Zhao YH, Wang DP, Zhang LL, Zhang F, Wang DM, Zhang WY (2011) Genomic expression profiles of blood and placenta reveal significant immune-related pathways and categories in Chinese women with gestational diabetes mellitus. Diabet Med 28: 237-46.
4. Shen X, Hu Y, Jiang Y, Liu H, Zhu L, et al. (2013) Krüppel-like factor 12 negatively regulates human endometrial stromal cell decidualization. Biochem Biophys Res Commun 433: 11-7.
5. Milewicz DM (1998) Molecular genetics of Marfan syndrome and Ehlers-Danlos type IV. Curr Opin Cardiol 13: 198-204.
6. Anum EA, Hill LD, Pandya A, Strauss JF 3rd (2009) Connective tissue and related disorders and preterm birth: clues to genes contributing to prematurity. Placenta 30: 207-15.
7. Costa AM, Maximiano EB, Avvad-Portari E, Jésus NR, Levy RA, Porto LC (2006) Contractile cells and fibrillin-1 distribution is disturbed in terminal villi of placentae from patients with preeclampsia and systemic lupus erythematosus. Placenta 27: 234-43.
8. Pulizzi N, Lyssenko V, Jonsson A, Osmond C, Laakso M, et al. (2009) Interaction between prenatal growth and high-risk genotypes in the development of type 2 diabetes. Diabetologia 52: 825-9.
9. Qi L, Liang J (2010) Interactions between genetic factors that predict diabetes and dietary factors that ultimately impact on risk of diabetes. Curr Opin Lipidol 21: 31-7.
10. [Stuebe AM](http://www.ncbi.nlm.nih.gov/pubmed?term=Stuebe%20AM%5BAuthor%5D&cauthor=true&cauthor_uid=23456907), [Wise A](http://www.ncbi.nlm.nih.gov/pubmed?term=Wise%20A%5BAuthor%5D&cauthor=true&cauthor_uid=23456907), [Nguyen T](http://www.ncbi.nlm.nih.gov/pubmed?term=Nguyen%20T%5BAuthor%5D&cauthor=true&cauthor_uid=23456907), [Herring A](http://www.ncbi.nlm.nih.gov/pubmed?term=Herring%20A%5BAuthor%5D&cauthor=true&cauthor_uid=23456907), [North KE](http://www.ncbi.nlm.nih.gov/pubmed?term=North%20KE%5BAuthor%5D&cauthor=true&cauthor_uid=23456907), [Siega-Riz AM](http://www.ncbi.nlm.nih.gov/pubmed?term=Siega-Riz%20AM%5BAuthor%5D&cauthor=true&cauthor_uid=23456907) (2014) [Maternal genotype and gestational diabetes.](http://www.ncbi.nlm.nih.gov/pubmed/23456907) [Am J Perinatol](http://www.ncbi.nlm.nih.gov/pubmed/23456907) 31: 69-76.
11. Jia RZ, Zhang X, Hu P, Liu XM, Hua XD, Wang X, Ding HJ (2012) Screening for differential methylation status in human placenta in preeclampsia using a CpG island plus promoter microarray. Int J Mol Med 30: 133-41.
12. Clarson LH, Roberts VH, Hamark B, Elliott AC, Powell T (2003) Store-operated Ca2+ entry in first trimester and term human placenta. J Physiol 550: 515-28.
13. Li J, Tan Z, Li MT, Liu YL, Liu Q, et al. (2006) Study of altered expression of annexin IV and human endometrial receptivity. Zhonghua Fu Chan Ke Za Zhi 41: 803-5.
14. [Masuda J](http://www.ncbi.nlm.nih.gov/pubmed?term=Masuda%20J%5BAuthor%5D&cauthor=true&cauthor_uid=15175799), [Takayama E](http://www.ncbi.nlm.nih.gov/pubmed?term=Takayama%20E%5BAuthor%5D&cauthor=true&cauthor_uid=15175799), [Satoh A](http://www.ncbi.nlm.nih.gov/pubmed?term=Satoh%20A%5BAuthor%5D&cauthor=true&cauthor_uid=15175799), [Ida M](http://www.ncbi.nlm.nih.gov/pubmed?term=Ida%20M%5BAuthor%5D&cauthor=true&cauthor_uid=15175799), [Shinohara T](http://www.ncbi.nlm.nih.gov/pubmed?term=Shinohara%20T%5BAuthor%5D&cauthor=true&cauthor_uid=15175799), et al. (2004) Levels of annexin IV and V in the plasma of pregnant and postpartum women. [Thromb Haemost](http://www.ncbi.nlm.nih.gov/pubmed/?term=Levels+of+annexin+IV+and+V+in+the+plasma+of+pregnant+and+postpartum+women.) 91: 1129-36.
15. Ulander VM, Stefanovic V, Masuda J, Suzuki K, Hiilesmaa V, Kaaja R (2007) Plasma levels of annexins IV and V in relation to antiphospholipid antibody status in women with a history of recurrent miscarriage. Thromb Res 120: 865-70.
16. Allegra A, Marino A, Peregrin PC, Lama A, García-Segovia A, et al. (2012) Endometrial expression of selected genes in patients achieving pregnancy spontaneously or after ICSI and patients failing at least two ICSI cycles. Reprod Biomed Online 25: 481-91.
17. Curtis S, Jones CJ, Garrod A, Hulme CH, Heazell AE (2013) Identification of autophagic vacuoles and regulators of autophagy in villous trophoblast from normal term pregnancies and in fetal growth restriction. J Matern Fetal Neonatal Med 26: 339-46.
18. [Tal R](http://www.ncbi.nlm.nih.gov/pubmed?term=Tal%20R%5BAuthor%5D&cauthor=true&cauthor_uid=23034156) (2012) The role of hypoxia and hypoxia-inducible factor-1alpha in preeclampsia pathogenesis. [Biol Reprod](http://www.ncbi.nlm.nih.gov/pubmed/23034156) 87: 134.
19. [Sezer SD](http://www.ncbi.nlm.nih.gov/pubmed?term=Sezer%20SD%5BAuthor%5D&cauthor=true&cauthor_uid=23767832), [Küçük M](http://www.ncbi.nlm.nih.gov/pubmed?term=K%C3%BC%C3%A7%C3%BCk%20M%5BAuthor%5D&cauthor=true&cauthor_uid=23767832), [Döger FK](http://www.ncbi.nlm.nih.gov/pubmed?term=D%C3%B6ger%20FK%5BAuthor%5D&cauthor=true&cauthor_uid=23767832), [Yüksel H](http://www.ncbi.nlm.nih.gov/pubmed?term=Y%C3%BCksel%20H%5BAuthor%5D&cauthor=true&cauthor_uid=23767832), [Odabaşi AR](http://www.ncbi.nlm.nih.gov/pubmed?term=Odaba%C5%9Fi%20AR%5BAuthor%5D&cauthor=true&cauthor_uid=23767832), et al. (2013) VEGF, PIGF and HIF-1α in placentas of early- and late-onset pre-eclamptic patients. [Gynecol Endocrinol](http://www.ncbi.nlm.nih.gov/pubmed/23767832) 29: 797-800.
20. Kim YS, Hori M, Yasuda K, Ozaki H (2005) Differences in the gestational pattern of mRNA expression of the Rnd family in rat and human myometria. Comp Biochem Physiol A Mol Integr Physiol 142: 410-5.
21. Lartey J, Gampel A, Pawade J, Mellor H, Bernal AL (2006) Expression of RND proteins in human myometrium. Biol Reprod 75: 452-61.
22. Johnson MP, Brennecke SP, East CE, Dyer TD, Roten LT, et al. (2013) Genetic dissection of the pre-eclampsia susceptibility locus on chromosome 2q22 reveals shared novel risk factors for cardiovascular disease. Mol Hum Reprod 19: 423-37.
23. Kitroser E1, Pomeranz M, Epstein Shochet G, Fishman A, Drucker L, et al. (2012) The involvement of eukaryotic translation initiation factor 4E in extravillous trophoblast cell function. Placenta 33: 717-24.
24. Pérez-Pérez A, Maymó J, Gambino Y, Duen~as JL, Goberna R, et al. (2009) Leptin stimulates protein synthesis-activating translation machinery in human trophoblastic cells. Biol Reprod 81: 826-32.
25. Zhao YJ, Zou QY, Li Y, Li HH, Wu YM, et al. (2014) Expression of G-protein subunit α-14 is increased in human placentas from preeclamptic pregnancies. J Histochem Cytochem 62: 347-54.
26. Moore F, Da Silva C, Wilde JI, Smarason A, Watson SP, López Bernal A (2000) Up-regulation of p21- and RhoA-activated protein kinases in human pregnant myometrium. Biochem Biophys Res Commun 269: 322-6.
27. Siu MK, Yeung MC, Zhang H, Kong DS, Ho JW, et al. (2010) p21-Activated kinase-1 promotes aggressive phenotype, cell proliferation, and invasion in gestational trophoblastic disease. Am J Pathol 176: 3015-22.
28. Gilli F, Lindberg RL, Valentino P, Marnetto F, Malucchi S, et al. (2010) Learning from nature: pregnancy changes the expression of inflammation-related genes in patients with multiple sclerosis. PLoS One 5: e8962.
29. O'Tierney PF, Lewis RM, McWeeney SK, Hanson MA, Inskip HM, et al. (2012) Immune response gene profiles in the term placenta depend upon maternal muscle mass. Reprod Sci 19: 1041-56.
30. Patni S, Wynen LP, Seager AL, Morgan G, White JO, Thornton CA (2009) Expression and activity of Toll-like receptors 1-9 in the human term placenta and changes associated with labor at term. Biol Reprod 80: 243-8.
31. Roduit C, Wohlgensinger J, Frei R, Bitter S, Bieli C, et al. (2011) Prenatal animal contact and gene expression of innate immunity receptors at birth are associated with atopic dermatitis. J Allergy Clin Immunol 127: 179-85.
32. Gillaux C, Méhats C, Vaiman D, Cabrol D, Breuiller-Fouché M (2011) Functional screening of TLRs in human amniotic epithelial cells. J Immunol 187: 2766-74.
33. [Panda B](http://www.ncbi.nlm.nih.gov/pubmed?term=Panda%20B%5BAuthor%5D&cauthor=true&cauthor_uid=22440523), [Panda A](http://www.ncbi.nlm.nih.gov/pubmed?term=Panda%20A%5BAuthor%5D&cauthor=true&cauthor_uid=22440523), [Ueda I](http://www.ncbi.nlm.nih.gov/pubmed?term=Ueda%20I%5BAuthor%5D&cauthor=true&cauthor_uid=22440523), [Abrahams VM](http://www.ncbi.nlm.nih.gov/pubmed?term=Abrahams%20VM%5BAuthor%5D&cauthor=true&cauthor_uid=22440523), [Norwitz ER](http://www.ncbi.nlm.nih.gov/pubmed?term=Norwitz%20ER%5BAuthor%5D&cauthor=true&cauthor_uid=22440523), et al. (2012) Dendritic cells in the circulation of women with preeclampsia demonstrate a pro-inflammatory bias secondary to dysregulation of TLR receptors. [J Reprod Immunol](http://www.ncbi.nlm.nih.gov/pubmed/?term=34.%09Dendritic+cells+in+the+circulation+of+women+with+preeclampsia+demonstrate+a+pro-inflammatory+bias+secondary+to+dysregulation) 94: 210-5.
34. Iwanaga N, Yamamasu S, Tachibana D, Nishio J, Nakai Y, et al. (2004) Activity of synthetic enzymes of tetrahydrobiopterin in the human placenta. Int J Mol Med 13: 117-20.
35. [Brüggemann N](http://www.ncbi.nlm.nih.gov/pubmed?term=Br%C3%BCggemann%20N%5BAuthor%5D&cauthor=true&cauthor_uid=22473768), [Spiegler J](http://www.ncbi.nlm.nih.gov/pubmed?term=Spiegler%20J%5BAuthor%5D&cauthor=true&cauthor_uid=22473768), [Hellenbroich Y](http://www.ncbi.nlm.nih.gov/pubmed?term=Hellenbroich%20Y%5BAuthor%5D&cauthor=true&cauthor_uid=22473768), [Opladen T](http://www.ncbi.nlm.nih.gov/pubmed?term=Opladen%20T%5BAuthor%5D&cauthor=true&cauthor_uid=22473768), [Schneider SA](http://www.ncbi.nlm.nih.gov/pubmed?term=Schneider%20SA%5BAuthor%5D&cauthor=true&cauthor_uid=22473768), et al. (2012) Beneficial prenatal levodopa therapy in autosomal recessive guanosine triphosphate cyclohydrolase 1 deficiency. [Arch Neurol](http://www.ncbi.nlm.nih.gov/pubmed/?term=36.%09Beneficial+prenatal+levodopa+therapy+in+autosomal+recessive+guanosine+triphosphate+cyclohydrolase+1deficiency) 69: 1071-5.
36. Liao C, Fu F, Li R, Pan M, Yang X, et al. (2012) Dandy-walker syndrome and microdeletions on chromosome 7. Zhonghua Yi Xue Yi Chuan Xue Za Zhi 29: 48-51.
37. Liao C, Fu F, Li R, Yang X, Xu Q, Li DZ (2012) Prenatal diagnosis and molecular characterization of a novel locus for Dandy-Walker malformation on chromosome 7p21.3. Eur J Med Genet 55: 472-5.
38. Wang L, Feng Y, Zhang Y, Zhou H, Jiang S, et al. (2006) Prolylcarboxypeptidase gene, chronic hypertension, and risk of preeclampsia. Am J Obstet Gynecol 195: 162-71.
39. Chang CW, Chang GD, Chen H (2011) A novel cyclic AMP/Epac1/CaMKI signaling cascade promotes GCM1 desumoylation and placental cell fusion. Mol Cell Biol 31: 3820-31.
40. Hamrick SE, Olshan AF, Neglia JP, Pollock BH (2001) Association of pregnancy history and birth characteristics with neuroblastoma: a report from the Children's Cancer Group and the Pediatric Oncology Group. Paediatr Perinat Epidemiol 15: 328-37.
41. Lee SH, Lee S, Jun HS, Jeong HJ, Cha WT, et al. (2003) Expression of the mitochondrial ATPase6 gene and Tfam in Down syndrome. Mol Cells 15: 181-5.
42. Pejznochova M, Tesarova M, Hansikova H, Magner M, Honzik T, et al. (2010) Mitochondrial DNA content and expression of genes involved in mtDNA transcription, regulation and maintenance during human fetal development. Mitochondrion 10: 321-9.
43. Sankaralingam S, Lalu MM, Xu Y, Davidge ST (2010) Effect of peroxynitrite scavenging on endothelial cells stimulated by plasma from women with preeclampsia: a proteomic approach. Hypertens Pregnancy 29: 419-28.
44. [Gharesi-Fard B](http://www.ncbi.nlm.nih.gov/pubmed?term=Gharesi-Fard%20B%5BAuthor%5D&cauthor=true&cauthor_uid=24621454), [Zolghadri J](http://www.ncbi.nlm.nih.gov/pubmed?term=Zolghadri%20J%5BAuthor%5D&cauthor=true&cauthor_uid=24621454), [Kamali-Sarvestani E](http://www.ncbi.nlm.nih.gov/pubmed?term=Kamali-Sarvestani%20E%5BAuthor%5D&cauthor=true&cauthor_uid=24621454) (2014) Alteration in the expression of proteins in unexplained recurrent pregnancy loss compared with in the normal placenta. [J Reprod Dev](http://www.ncbi.nlm.nih.gov/pubmed/?term=45.%09Alteration+in+the+expression+of+proteins+in+unexplained+recurrent+pregnancy+loss+compared+with+in+the+normal+placenta) 60: 261-7.
45. Wang Y, Gu Y, Granger DN, Roberts JM, Alexander JS (2002) Endothelial junctional protein redistribution and increased monolayer permeability in human umbilical vein endothelial cells isolated during preeclampsia. Am J Obstet Gynecol 186: 214-20.
46. [Wang Y](http://www.ncbi.nlm.nih.gov/pubmed?term=Wang%20Y%5BAuthor%5D&cauthor=true&cauthor_uid=15126573), [Lewis DF](http://www.ncbi.nlm.nih.gov/pubmed?term=Lewis%20DF%5BAuthor%5D&cauthor=true&cauthor_uid=15126573), [Gu Y](http://www.ncbi.nlm.nih.gov/pubmed?term=Gu%20Y%5BAuthor%5D&cauthor=true&cauthor_uid=15126573), [Zhang Y](http://www.ncbi.nlm.nih.gov/pubmed?term=Zhang%20Y%5BAuthor%5D&cauthor=true&cauthor_uid=15126573), [Alexander JS](http://www.ncbi.nlm.nih.gov/pubmed?term=Alexander%20JS%5BAuthor%5D&cauthor=true&cauthor_uid=15126573), [Granger DN](http://www.ncbi.nlm.nih.gov/pubmed?term=Granger%20DN%5BAuthor%5D&cauthor=true&cauthor_uid=15126573) (2004) Placental trophoblast-derived factors diminish endothelial barrier function. [J Clin Endocrinol Metab](http://www.ncbi.nlm.nih.gov/pubmed/?term=47.%09Placental+trophoblast-derived+factors+diminish+endothelial+barrier+function.) 89: 2421-8.
47. Korgun ET, Celik-Ozenci C, Acar N, Cayli S, Desoye G, Demir R (2006) Location of cell cycle regulators cyclin B1, cyclin A, PCNA, Ki67 and cell cycle inhibitors p21, p27 and p57 in human first trimester placenta and deciduas. Histochem Cell Biol 125: 615-24.
48. Taylor RN, Varma M, Teng NN, Roberts JM (1990) Women with preeclampsia have higher plasma endothelin levels than women with normal pregnancies. J Clin Endocrinol Metab 71: 1675-7.
49. Nishikawa S, Miyamoto A, Yamamoto H, Ohshika H, Kudo R (2000) The relationship between serum nitrate and endothelin-1 concentrations in preeclampsia. Life Sci 67: 1447–54.
50. [Yi KW](http://www.ncbi.nlm.nih.gov/pubmed?term=Yi%20KW%5BAuthor%5D&cauthor=true&cauthor_uid=24231447), [Jung SH](http://www.ncbi.nlm.nih.gov/pubmed?term=Jung%20SH%5BAuthor%5D&cauthor=true&cauthor_uid=24231447), [Cho GJ](http://www.ncbi.nlm.nih.gov/pubmed?term=Cho%20GJ%5BAuthor%5D&cauthor=true&cauthor_uid=24231447), [Seol HJ](http://www.ncbi.nlm.nih.gov/pubmed?term=Seol%20HJ%5BAuthor%5D&cauthor=true&cauthor_uid=24231447), [Hong SC](http://www.ncbi.nlm.nih.gov/pubmed?term=Hong%20SC%5BAuthor%5D&cauthor=true&cauthor_uid=24231447), et al. (2014) Effects of sFlt-1 and alpha 2-macroglobulin on vascular endothelial growth factor-induced endothelin-1 upregulation in human microvascular endothelial cells. [Placenta](http://www.ncbi.nlm.nih.gov/pubmed/24231447) 35: 64-9.
51. Kolisek M, Galaviz-Hernández C, Vázquez-Alaniz F, Sponder G, Javaid S, et al. (2013) SLC41A1 is the only magnesium responsive gene significantly overexpressed in placentas of preeclamptic women. Hypertens Pregnancy 32: 378-89.
52. Godoi LC, Gomes KB, Alpoim PN, Carvalho Md, Lwaleed BA, Sant'Ana Dusse LM (2012) Preeclampsia: the role of tissue factor and tissue factor pathway inhibitor. J Thromb Thrombolysis 34: 1-6.
53. Teng Y, Jiang R, Lin Q, Ding C, Ye Z (2010) The relationship between plasma and placental tissue factor, and tissue factor pathway inhibitors in severe pre-eclampsia patients. Thromb Res 126: e41-5.
54. Di Paolo S, Volpe P, Grandaliano G, Stallone G, Schena A, et al. (2003) Increased placental expression of tissue factor is associated with abnormal uterine and umbilical Doppler waveforms in severe preeclampsia with fetal growth restriction. J Nephrol 16: 650-7.
55. Erez O, Romero R, Hoppensteadt D, Than NG, Fareed J, et al. (2008) Tissue factor and its natural inhibitor in pre-eclampsia and SGA. J Matern Fetal Neonatal Med 21: 855-69.
56. Horne AW, Phillips JA 3rd, Kane N, Lourenco PC, McDonald SE, et al. (2008) CB1 expression is attenuated in Fallopian tube and decidua of women with ectopic pregnancy. PLoS One 3: e3969.
57. Bienertova-Vasku J, Bienert P, Dostalova Z, Chovanec J, Vasku A, Vasku V (2011) A common variation in the cannabinoid 1 receptor (CNR1) gene is associated with pre-eclampsia in the Central European population. Eur J Obstet Gynecol Reprod Biol 155: 19-22.
58. Vatansever HS, Inan VS, Lacin S, Koyuncu F (2003) Immunolocalization of alphaV, alpha3 and beta1 integrins in the human placenta with pre-eclampsia. Acta Histochem 105: 253-60.
59. Lorenzi T, Turi A, Crescimanno C, Morroni M, Castellucci M, et al. (2010) Syndecan expressions in the human amnion and chorionic plate. Eur J Histochem 54: e42.
60. Chui A, Zainuddin N, Rajaraman G, Murthi P, Brennecke SP, et al. (2012) Placental syndecan expression is altered in human idiopathic fetal growth restriction. Am J Pathol 180: 693-702.
61. Rajakumar A, Chu T, Handley DE, Bunce KD, Burke B, et al. (2011) Maternal gene expression profiling during pregnancy and preeclampsia in human peripheral blood mononuclear cells. Placenta 32: 70-8.
62. Vaiman D, Calicchio R, Miralles F (2013) Landscape of transcriptional deregulations in the preeclamptic placenta. PLoS One 8: e65498.
63. Olvera M, Harris S, Amezcua CA, McCourty A, Rezk S, et al. (2001) Immunohistochemical expression of cell cycle proteins E2F-1, Cdk-2, Cyclin E, p27(kip1), and Ki-67 in normal placenta and gestational trophoblastic disease. Mod Pathol 14: 1036-42.
64. [Huang Q](http://www.ncbi.nlm.nih.gov/pubmed?term=Huang%20Q%5BAuthor%5D&cauthor=true&cauthor_uid=23333240), [Li J](http://www.ncbi.nlm.nih.gov/pubmed?term=Li%20J%5BAuthor%5D&cauthor=true&cauthor_uid=23333240), [Wang F](http://www.ncbi.nlm.nih.gov/pubmed?term=Wang%20F%5BAuthor%5D&cauthor=true&cauthor_uid=23333240), [Oliver MT](http://www.ncbi.nlm.nih.gov/pubmed?term=Oliver%20MT%5BAuthor%5D&cauthor=true&cauthor_uid=23333240), [Tipton T](http://www.ncbi.nlm.nih.gov/pubmed?term=Tipton%20T%5BAuthor%5D&cauthor=true&cauthor_uid=23333240), et al. (2013) Syncytin-1 modulates placental trophoblast cell proliferation by promoting G1/S transition. [Cell Signal](http://www.ncbi.nlm.nih.gov/pubmed/?term=Syncytin-1+modulates+placental+trophoblast+cell+proliferation+by+promoting+G1%2FS+transition.) 25: 1027-35.
65. McMinn J, Wei M, Schupf N, Cusmai J, Johnson EB, et al. (2006) Unbalanced placental expression of imprinted genes in human intrauterine growth restriction. Placenta 27: 540-9.
66. Sahlin L, Ostlund E, Wang H, Holmgren A, Fried G (2000) Decreased expression of thioredoxin and glutaredoxin in placentae from pregnancies with pre-eclampsia and intrauterine growth restriction. Placenta 21: 603-9.
67. Shibata E, Ejima K, Nanri H, Toki N, Koyama C, et al. (2001) Enhanced protein levels of protein thiol/disulphide oxidoreductases in placentae from pre-eclamptic subjects. Placenta 22: 566-72.
68. Wang X (2008) miRDB: a microRNA target prediction and functional annotation database with a wiki interface. RNA 14(6):1012-1017

<http://mirdb.org/cgi-bin/search.cgi>

1. Hamel M, Dufort I, Robert C, Léveillé MC, Leader A, Sirard M (2010) Genomic assessment of follicular marker genes as pregnancy predictors for human IVF. Mol Hum Reprod 16: 87-96.
2. Kvehaugen AS, Melien O, Holmen OL, Laivuori H, Oian P, et al. (2013) Single nucleotide polymorphisms in G protein signaling pathway genes in preeclampsia. Hypertension 61: 655-61.
3. Zhu H, Leung PC, MacCalman CD (2007) Expression of ADAMTS-5/implantin in human decidual stromal cells: regulatory effects of cytokines. Hum Reprod 22: 63-74.
4. Lee SY, Lee HS, Gil M, Kim CJ, Lee YH, et al. (2014) Differential expression patterns of a disintegrin and metalloproteinase with thrombospondin motifs (ADAMTS) -1, -4, -5, and -14 in human placenta and gestational trophoblastic diseases. Arch Pathol Lab Med 138: 643-50.
5. Chung J, Tsai S, James AH, Thames BH, Shytle S, Piedrahita JA (2012) Lack of genomic imprinting of DNA primase, polypeptide 2 (PRIM2) in human term placenta and white blood cells. Epigenetics 7: 429-31.
6. Lok CA, Jebbink J, Nieuwland R, Faas MM, Boer K, et al. (2009) Leukocyte activation and circulating leukocyte-derived microparticles in preeclampsia. Am J Reprod Immunol 61: 346-59.
7. [Shang W](http://www.ncbi.nlm.nih.gov/pubmed?term=Shang%20W%5BAuthor%5D&cauthor=true&cauthor_uid=24379070), [Shu MM](http://www.ncbi.nlm.nih.gov/pubmed?term=Shu%20MM%5BAuthor%5D&cauthor=true&cauthor_uid=24379070), [Liu M](http://www.ncbi.nlm.nih.gov/pubmed?term=Liu%20M%5BAuthor%5D&cauthor=true&cauthor_uid=24379070), [Wang AM](http://www.ncbi.nlm.nih.gov/pubmed?term=Wang%20AM%5BAuthor%5D&cauthor=true&cauthor_uid=24379070), [Lv LB](http://www.ncbi.nlm.nih.gov/pubmed?term=Lv%20LB%5BAuthor%5D&cauthor=true&cauthor_uid=24379070), et al. (2013) Elevated expressions of p53, CDKNA1, and Bax in placental villi from patients with recurrent spontaneous abortion. [Eur Rev Med Pharmacol Sci](http://www.ncbi.nlm.nih.gov/pubmed/?term=Elevated+expressions+of+p53%2C+CDKNA1%2C+and+Bax+in+placental+villi+from+patients+with+recurrent+spontaneous+abortion) 17: 3376-80.
8. Assou S, Haouzi D, Mahmoud K, Aouacheria A, Guillemin Y, et al. (2008) A non-invasive test for assessing embryo potential by gene expression profiles of human cumulus cells: a proof of concept study. Mol Hum Reprod 14: 711-9.
9. Hamamah S1, Fallet C (2010) Gene expression profile of human cumulus cells: clinical applications for IVF. J Gynecol Obstet Biol Reprod (Paris) 39: 5-7.
10. Shaarawy M, El Meleigy M, Rasheed K (2001) Maternal serum transforming growth factor beta-2 in preeclampsia and eclampsia, a potential biomarker for the assessment of disease severity and fetal outcome. J Soc Gynecol Investig 8: 27-31.
11. Lyall F, Simpson H, Bulmer JN, Barber A, Robson SC (2001) Transforming growth factor-beta expression in human placenta and placental bed in third trimester normal pregnancy, preeclampsia, and fetal growth restriction. Am J Pathol 159: 1827-38.
12. Simpson H, Robson SC, Bulmer JN, Barber A, Lyall F (2002) Transforming growth factor beta expression in human placenta and placental bed during early pregnancy. Placenta 23: 44-58.
13. Lozoya T, Domínguez F, Romero-Ruiz A, Steffani L, Martínez S, et al. (2014) The Lin28/Let-7 system in early human embryonic tissue and ectopic pregnancy. PLoS One 9: e87698.
14. Plösch T, Gellhaus A, van Straten EM, Wolf N, Huijkman NC, et al. (2010) The liver X receptor (LXR) and its target gene ABCA1 are regulated upon low oxygen in human trophoblast cells: a reason for alterations in preeclampsia? Placenta 31: 910-8.
15. Baumann M, Körner M, Huang X, Wenger F, Surbek D, Albrecht C (2013) Placental ABCA1 and ABCG1 expression in gestational disease: Pre-eclampsia affects ABCA1 levels in syncytiotrophoblasts. Placenta 34: 1079-86.
16. Wittwer CU, Bauw G, Krokan HE (1989) Purification and determination of the NH2-terminal amino acid sequence of uracil-DNA glycosylase from human placenta. Biochemistry 28: 780-4.
17. Krokan H, Haugen A, Myrnes B, Guddal PH (1983) Repair of premutagenic DNA lesions in human fetal tissues: evidence for low levels of O6-methylguanine-DNA methyltransferase and uracil-DNA glycosylase activity in some tissues. Carcinogenesis 4: 1559-64.
18. Joubert BR, Haberg SE, Nilsen RM, Wang X, Vollset SE, et al. (2012) 450K epigenome-wide scan identifies differential DNA methylation in newborns related to maternal smoking during pregnancy. Environ Health Perspect 120: 1425-31.
19. Wang Y, Nie M, Li W, Ping F, Hu Y, et al. (2011) Association of six single nucleotide polymorphisms with gestational diabetes mellitus in a Chinese population. PLoS One 6: e26953.
20. [Cho YM](http://www.ncbi.nlm.nih.gov/pubmed?term=Cho%20YM%5BAuthor%5D&cauthor=true&cauthor_uid=19002430), [Kim TH](http://www.ncbi.nlm.nih.gov/pubmed?term=Kim%20TH%5BAuthor%5D&cauthor=true&cauthor_uid=19002430), [Lim S](http://www.ncbi.nlm.nih.gov/pubmed?term=Lim%20S%5BAuthor%5D&cauthor=true&cauthor_uid=19002430), [Choi SH](http://www.ncbi.nlm.nih.gov/pubmed?term=Choi%20SH%5BAuthor%5D&cauthor=true&cauthor_uid=19002430), [Shin HD](http://www.ncbi.nlm.nih.gov/pubmed?term=Shin%20HD%5BAuthor%5D&cauthor=true&cauthor_uid=19002430), et al. (2009) Type 2 diabetes-associated genetic variants discovered in the recent genome-wide association studies are related to gestational diabetes mellitus in the Korean population. [Diabetologia](http://www.ncbi.nlm.nih.gov/pubmed/?term=Type+2+diabetes-associated+genetic+variants+discovered+in+the+recent+genome-wide+association+studies+are+related+to+gestational+diabetes+mellitus+in+the+Korean+population) 252: 253-61.
21. Wu HY, Lin CY, Lin TY, Chen TC, Yuan CJ (2008) Mammalian Ste20-like protein kinase 3 mediates trophoblast apoptosis in spontaneous delivery. Apoptosis 13: 283-94.
22. Meng T, Chen HY, Li J, Shang T (2008) Expression of lectin-liked oxidized low density lipoprotein receptor-1 and apoptosis associated genes in placenta and the relationship thereof with morbility of early-onset preeclampsia. Zhonghua Yi Xue Za Zhi 88: 2633-5.
23. Zhang Y, Ye YH, Peng W, Zhan Y (2009) Correlation of oxidized low-density lipoprotein and lectin-like oxidized low-density lipoprotein receptor-1 with pre-eclampsia. Zhonghua Fu Chan Ke Za Zhi 44: 94-8.
24. van Rijn BB, Franx A, Steegers EA, de Groot CJ, Bertina RM, et al. (2008) Maternal TLR4 and NOD2 gene variants, pro-inflammatory phenotype and susceptibility to early-onset preeclampsia and HELLP syndrome. PLoS One 3: e1865.
25. Lappas M (2013) NOD1 and NOD2 regulate proinflammatory and prolabor mediators in human fetal membranes and myometrium via nuclear factor-kappa B. Biol Reprod 89: 14.
26. Härtel Ch, Finas D, Ahrens P, Kattner E, Schaible T, et al. (2004) Polymorphisms of genes involved in innate immunity: association with preterm delivery. Mol Hum Reprod 10: 911-5.
27. [Jang DG](http://www.ncbi.nlm.nih.gov/pubmed?term=Jang%20DG%5BAuthor%5D&cauthor=true&cauthor_uid=21790888), [Chae H](http://www.ncbi.nlm.nih.gov/pubmed?term=Chae%20H%5BAuthor%5D&cauthor=true&cauthor_uid=21790888), [Shin JC](http://www.ncbi.nlm.nih.gov/pubmed?term=Shin%20JC%5BAuthor%5D&cauthor=true&cauthor_uid=21790888), [Park IY](http://www.ncbi.nlm.nih.gov/pubmed?term=Park%20IY%5BAuthor%5D&cauthor=true&cauthor_uid=21790888), [Kim M](http://www.ncbi.nlm.nih.gov/pubmed?term=Kim%20M%5BAuthor%5D&cauthor=true&cauthor_uid=21790888), [Kim Y](http://www.ncbi.nlm.nih.gov/pubmed?term=Kim%20Y%5BAuthor%5D&cauthor=true&cauthor_uid=21790888) (2011) Prenatal diagnosis of autosomal recessive polycystic kidney disease by molecular genetic analysis. [J Obstet Gynaecol Res](http://www.ncbi.nlm.nih.gov/pubmed/21790888) 37: 1744-7.
28. Wang L, Wang X, Laird N, Zuckerman B, Stubblefield P, Xu X (2006) Polymorphism in maternal LRP8 gene is associated with fetal growth. Am J Hum Genet 78: 770-7.
29. Hemmings DG, Hudson NK, Halliday D, O'Hara M, Baker PN, et al. (2006) Sphingosine-1-phosphate acts via rho-associated kinase and nitric oxide to regulate human placental vascular tone. Biol Reprod 74: 88-94.
30. Hudson NK, O'Hara M, Lacey HA, Corcoran J, Hemmings DG, et al. (2007) Modulation of human arterial tone during pregnancy: the effect of the bioactive metabolite sphingosine-1-phosphate. Biol Reprod 77: 45-52.
31. [Brünnert D](http://www.ncbi.nlm.nih.gov/pubmed?term=Br%C3%BCnnert%20D%5BAuthor%5D&cauthor=true&cauthor_uid=24994816), [Sztachelska M](http://www.ncbi.nlm.nih.gov/pubmed?term=Sztachelska%20M%5BAuthor%5D&cauthor=true&cauthor_uid=24994816), [Bornkessel F](http://www.ncbi.nlm.nih.gov/pubmed?term=Bornkessel%20F%5BAuthor%5D&cauthor=true&cauthor_uid=24994816), [Treder N](http://www.ncbi.nlm.nih.gov/pubmed?term=Treder%20N%5BAuthor%5D&cauthor=true&cauthor_uid=24994816), [Wolczynski S](http://www.ncbi.nlm.nih.gov/pubmed?term=Wolczynski%20S%5BAuthor%5D&cauthor=true&cauthor_uid=24994816), et al. (2014) Lysophosphatidic acid and sphingosine 1-phosphate metabolic pathways and their receptors aredifferentially regulated during decidualization of human endometrial stromal cells. [Mol Hum Reprod](http://www.ncbi.nlm.nih.gov/pubmed/?term=Lysophosphatidic+acid+and+sphingosine+1-phosphate+metabolic+pathways+and+their+receptors+are+differentially+regulated+during+decidualization+of+human+endometrial+stromal+cells) 20: 1016-25.
32. Gloria-Bottini F, Meloni GF, Nicotra M, Saccucci P, Stampone L, et al. (2008) Feto-maternal ACP1 activity ratio and intrauterine survival. Eur J Obstet Gynecol Reprod Biol 140: 12-6.
33. Nicotra M, Bottini N, La Torre M, Amante A, Bottini E, Gloria-Bottini F (2007) Repeated spontaneous abortion. Cooperative effects of ADA and ACP1 genetic polymorphisms. Am J Reprod Immunol 58: 1-10.
34. Suzumori N, Kaname T, Muramatsu Y, Yanagi K, Kumagai K, et al. (2013) Prenatal diagnosis of X-linked recessive Lenz microphthalmia syndrome. J Obstet Gynaecol Res 39: 1545-7.
35. Gu Y, Burlison SA, Wang Y (2006) [PAF levels and PAF-AH activities in placentas from normal and preeclamptic pregnancies.](http://www.ncbi.nlm.nih.gov/pubmed/16122793) Placenta 27: 744-9.
36. [Fan P](http://www.ncbi.nlm.nih.gov/pubmed?term=Fan%20P%5BAuthor%5D&cauthor=true&cauthor_uid=22797139), [Liu XH](http://www.ncbi.nlm.nih.gov/pubmed?term=Liu%20XH%5BAuthor%5D&cauthor=true&cauthor_uid=22797139), [He GL](http://www.ncbi.nlm.nih.gov/pubmed?term=He%20GL%5BAuthor%5D&cauthor=true&cauthor_uid=22797139), [Zhang S](http://www.ncbi.nlm.nih.gov/pubmed?term=Zhang%20S%5BAuthor%5D&cauthor=true&cauthor_uid=22797139), [Zhang JX](http://www.ncbi.nlm.nih.gov/pubmed?term=Zhang%20JX%5BAuthor%5D&cauthor=true&cauthor_uid=22797139), [Bai H](http://www.ncbi.nlm.nih.gov/pubmed?term=Bai%20H%5BAuthor%5D&cauthor=true&cauthor_uid=22797139) (2012) Maternal and fetal plasma platelet-activating factor acetylhydrolase activity and distribution in pre-eclampsia. [Pediatr Res](http://www.ncbi.nlm.nih.gov/pubmed/?term=Maternal+and+fetal+plasma+platelet-activating+factor+acetylhydrolase+activity+and+distribution+in+pre-eclampsia.) 72: 426-31.
37. [Poon LC](http://www.ncbi.nlm.nih.gov/pubmed?term=Poon%20LC%5BAuthor%5D&cauthor=true&cauthor_uid=24764257), [Nicolaides KH](http://www.ncbi.nlm.nih.gov/pubmed?term=Nicolaides%20KH%5BAuthor%5D&cauthor=true&cauthor_uid=24764257) (2014) First-trimester maternal factors and biomarker screening for preeclampsia. [Prenat Diagn](http://www.ncbi.nlm.nih.gov/pubmed/24764257) 34: 618-27.
38. National Collaborating Centre for Women's and Children's Health (UK). Hypertension in Pregnancy: the Management of Hypertensive Disorders During Pregnancy. London: RCOG Press, 2010.
39. World Health Organization, Dept. of Reproductive Health and Research, Dept. of Maternal, Newborn, Child and Adolescent Health,Dept. of Nutrition for Health and Development. WHO Recommendations for Prevention and Treatment of Pre-eclampsia and Eclampsia. Switzerland: World Health Organization, 2011.
40. Scioscia M, Gumaa K, Kunjara S, Paine MA, Selvaggi LE, et al. (2006) Insulin resistance in human preeclamptic placenta is mediated by serine phosphorylation of insulin receptor substrate-1 and -2. J Clin Endocrinol Metab 91: 709-17.
41. Machorro-Lazo MV, Sanchez-Corona J, Martínez-Abundis E, González-Ortiz M, Galaviz-Hernandez C, et al. (2009) Analysis of the association of preeclampsia with polymorphisms of the INS, INSR and IRS1 genes in Mexican women. Gynecol Obstet Invest 67: 14-9.
42. [Blau N](http://www.ncbi.nlm.nih.gov/pubmed?term=Blau%20N%5BAuthor%5D&cauthor=true&cauthor_uid=20971365), [van Spronsen FJ](http://www.ncbi.nlm.nih.gov/pubmed?term=van%20Spronsen%20FJ%5BAuthor%5D&cauthor=true&cauthor_uid=20971365), [Levy HL](http://www.ncbi.nlm.nih.gov/pubmed?term=Levy%20HL%5BAuthor%5D&cauthor=true&cauthor_uid=20971365) (2010) Phenylketonuria. [Lancet](http://www.ncbi.nlm.nih.gov/pubmed/?term=Blau+N%2C+van+Spronsen+FJ%2C+Levy+HL.++Phenylketonuria.+Lancet+2010%3B+376%3A+1417%E2%80%9327) 376: 1417-27.
43. Vogel MJ, van Zon P, Brueton L, Gijzen M, van Tuil MC, et al. (2012) Mutations in GRIP1 cause Fraser syndrome. J Med Genet 49: 303-6.
44. [Wang WJ](http://www.ncbi.nlm.nih.gov/pubmed?term=Wang%20WJ%5BAuthor%5D&cauthor=true&cauthor_uid=23450478), [Zhu HY](http://www.ncbi.nlm.nih.gov/pubmed?term=Zhu%20HY%5BAuthor%5D&cauthor=true&cauthor_uid=23450478), [Zhu RF](http://www.ncbi.nlm.nih.gov/pubmed?term=Zhu%20RF%5BAuthor%5D&cauthor=true&cauthor_uid=23450478), [Yang Y](http://www.ncbi.nlm.nih.gov/pubmed?term=Yang%20Y%5BAuthor%5D&cauthor=true&cauthor_uid=23450478), [Zhu XY](http://www.ncbi.nlm.nih.gov/pubmed?term=Zhu%20XY%5BAuthor%5D&cauthor=true&cauthor_uid=23450478), et al. (2013) Mutation analysis and prenatal diagnosis of families affected with Duchenne and Becker muscular dystrophy. [Zhonghua Yi Xue Yi Chuan Xue Za Zhi](http://www.ncbi.nlm.nih.gov/pubmed/23450478) 30: 45-8.
45. Deborde S, Schofield JN, Rademacher TW (2003) Placental GPI-PLD is of maternal origin and its GPI substrate is absent from placentae of pregnancies associated with pre-eclampsia. J Reprod Immunol 59: 277-94.
46. Dunk CE, Roggensack AM, Cox B, Perkins JE, Asenius F, et al. (2012) A distinct microvascular endothelial gene expression profile in severe IUGR placentas. Placenta 33: 285-93.
47. Zhou Z, Shen T, Zhang BH, Lv XY, Lin HY, et al. (2009) The proprotein convertase furin in human trophoblast: Possible role in promoting trophoblast cell migration and invasion. Placenta 30: 929-38.
48. Zhou Z, Zhang Q, Lu X, Wang R, Wang H, et al. (2013) The proprotein convertase furin is required for trophoblast syncytialization. Cell Death Dis 4: e593.
49. [Schneuer FJ](http://www.ncbi.nlm.nih.gov/pubmed?term=Schneuer%20FJ%5BAuthor%5D&cauthor=true&cauthor_uid=24215861), [Roberts CL](http://www.ncbi.nlm.nih.gov/pubmed?term=Roberts%20CL%5BAuthor%5D&cauthor=true&cauthor_uid=24215861), [Ashton AW](http://www.ncbi.nlm.nih.gov/pubmed?term=Ashton%20AW%5BAuthor%5D&cauthor=true&cauthor_uid=24215861), [Guilbert C](http://www.ncbi.nlm.nih.gov/pubmed?term=Guilbert%20C%5BAuthor%5D&cauthor=true&cauthor_uid=24215861), [Tasevski V](http://www.ncbi.nlm.nih.gov/pubmed?term=Tasevski%20V%5BAuthor%5D&cauthor=true&cauthor_uid=24215861), et al. (2014) Angiopoietin 1 and 2 serum concentrations in first trimester of pregnancy as biomarkers of adverse pregnancy outcomes. [Am J Obstet Gynecol](http://www.ncbi.nlm.nih.gov/pubmed/?term=Angiopoietin+1+and+2+serum+concentrations+in+first+trimester+of+pregnancy+as+biomarkers+of+adverse+pregnancyoutcomes) 210: 345.e1-9.
50. [Andraweera PH](http://www.ncbi.nlm.nih.gov/pubmed?term=Andraweera%20PH%5BAuthor%5D&cauthor=true&cauthor_uid=22205728), [Dekker GA](http://www.ncbi.nlm.nih.gov/pubmed?term=Dekker%20GA%5BAuthor%5D&cauthor=true&cauthor_uid=22205728), [Thompson SD](http://www.ncbi.nlm.nih.gov/pubmed?term=Thompson%20SD%5BAuthor%5D&cauthor=true&cauthor_uid=22205728), [North RA](http://www.ncbi.nlm.nih.gov/pubmed?term=North%20RA%5BAuthor%5D&cauthor=true&cauthor_uid=22205728), [McCowan LM](http://www.ncbi.nlm.nih.gov/pubmed?term=McCowan%20LM%5BAuthor%5D&cauthor=true&cauthor_uid=22205728), et al. (2012) A functional variant in ANGPT1 and the risk of pregnancies with hypertensive disorders and small-for-gestational-age infants. [Mol Hum Reprod](http://www.ncbi.nlm.nih.gov/pubmed/?term=A+functional+variant+in+ANGPT1+and+the+risk+of+pregnancies+with+hypertensive+disorders+and+small-for-gestational) 18: 325-32.
51. [Stoller JZ](http://www.ncbi.nlm.nih.gov/pubmed?term=Stoller%20JZ%5BAuthor%5D&cauthor=true&cauthor_uid=20463296), [Huang L](http://www.ncbi.nlm.nih.gov/pubmed?term=Huang%20L%5BAuthor%5D&cauthor=true&cauthor_uid=20463296), [Tan CC](http://www.ncbi.nlm.nih.gov/pubmed?term=Tan%20CC%5BAuthor%5D&cauthor=true&cauthor_uid=20463296), [Huang F](http://www.ncbi.nlm.nih.gov/pubmed?term=Huang%20F%5BAuthor%5D&cauthor=true&cauthor_uid=20463296), [Zhou DD](http://www.ncbi.nlm.nih.gov/pubmed?term=Zhou%20DD%5BAuthor%5D&cauthor=true&cauthor_uid=20463296), et al. (2010) Ash2l interacts with Tbx1 and is required during early embryogenesis. [Exp Biol Med (Maywood)](http://www.ncbi.nlm.nih.gov/pubmed/?term=Ash2l+interacts+with+Tbx1+and+is+required+during+early+embryogenesis.) 235: 569-76.
52. Stenzinger A, Märker D, Koch P, Hoffmann J, Baal N, et al. (2009) Protein tyrosine phosphatase interacting protein 51 (PTPIP51) mRNA expression and localization and its in vitro interacting partner protein tyrosine phosphatase 1B (PTP1B) in human placenta of the first, second, and third trimester. J Histochem Cytochem 57: 143-53.
